# Supplementary material for: The effects of supervision on three different exercises modalities (supervised vs. home vs. supervised+home) in older adults: Randomized controlled trial protocol
Source: PLoS One. 2021 Nov 15;16(11):e0259827. doi: 10.1371/journal.pone.0259827 (PMC8592418; doi:10.1371/journal.pone.0259827)
Supplement: S1 File — (PDF) [file pone.0259827.s001.pdf]

Projeto de Pesquisa

SEARCH DESCRIPTION

**Title**	Comparison of a multicomponent exercise program in center and/or home modality on neuromuscular function, muscle composition and muscle architecture of older adults
**Principal Researcher**	Paulo Cesar Barauce Bento
**Collaborators**	Jaciara do Carmo Frasão<br>Maria Luiza Tavares da Silva<br>Sabrine Nayara Costa
**Place**	Universidade Federal do Paraná - Departamento de Educação Física (DEF-UFPR)
**Search Period**	01/2020 – 12/2021

1. Research Objective

1.1 General Objective

This study aims to compare the effects of a multicomponent physical exercise program in different application modalities (face-to-face x home x mixed) and to verify neuromuscular adaptations in the community older adults.

1.1.2 Specific Objectives

- Compare the effects of an exercise program with home and group sessions (mixed) to the effects of a face-to-face and home program on musculoskeletal function (neuromuscular function, composition, and muscle architecture) of older adults in the community.
- Compare the effects of an exercise program with home and group sessions (mixed) to the effects of a face-to-face and home program on the parameters of time-space of gait and cost of the double task of older adults in the community.
- Compare the effects of an exercise program with home and group sessions (mixed) to the effects of a face-to-face and home program on the performance of a set of functional tests and quality of life of older adults in the community.
- Compare the speed of movement execution and intensity of training in face-to-face and home sessions in the three modalities of the multicomponent exercise program.
- Verify the effects of an exercise program with home and group sessions on the effects of a strictly face-to-face and home program after eight weeks of detraining in the community's older adults.

2. Social Relevance

Aging is a natural process that can be accompanied by physical, physiological, and psychological changes. Together these changes may result in the impairment of the functionality and quality of life of the older adults. In this sense, the regular practice of

physical exercises has been a strategy to counter the effects of aging, which can prevent or delay the development of chronic diseases and reduce the speed at which physical and physiological declines occur. Studies have shown that supervised and group-based physical exercise programs can mitigate the physical and functional decline, prevent frailty and its evolution, thus avoiding consequences such as the loss of independence of the older adults. However, face-to-face programs are associated with difficulties to the participant's attack, due to the lack of access to these types of programs near the residence, difficulties in locomotion, and dependence of family members or third parties to move the older adults to physical training places. Alternatively, the literature supports home exercise programs, which reduce these barriers and, once patients are committed to them, have long-term benefits. On the other hand, in this exercise modality, supervision is provided indirectly by phone calls or home visits, which may result in limitations regarding the quality of exercise, such as performing exercises with a lower range of motion, less vigorous intensity, and longer pauses, which results in a lower intensity of exercise when compared to face-to-face training. Thus, a possibility of a physical exercise program model for the older adults population is the combination of an exercise program with weekly face-to-face and home sessions. The face-to-face session would provide an increase in the social interaction of the older adults and greater supervision of the exercise since the main limitation of the home exercise is the lack of control of the frequency, intensity, and progression of the exercise.

3. Hypotheses to Be Tested

A twelve-week program with home and group (mixed) exercise sessions will improve neuromuscular function, gait, functionality, and quality of life of older adults in the community in a similar way to a face-to-face physical exercise program and will have a greater effect than the home program. In this way, the general hypotheses will be tested.

H0 – There will be no differences in the responses to physical training in the different modalities of application of the multicomponent exercise program.

H1 – The program with home and group (mixed) exercise sessions will present increased musculoskeletal function (neuromuscular function, composition, and muscle architecture) similar to those observed in the classroom physical exercise program and superior to the household group.

H2 – The program with home and group (mixed) exercise sessions will improve the time-space parameters of gait and cost of the double task similar to those observed in the exercise program strictly in-person and higher than the strictly home group.

H3 – The program with home and group (mixed) exercise sessions will show improvement in the performance of a set of functional tests and quality of life similar to those observed in the exercise program strictly in-person and superior to the strictly home group.

H4 – The speed of movement execution and training intensity will be higher in the sessions of the face-to-face group and the mixed group when purchased from the strictly home group.

H5 - The effects of an exercise program with home and group sessions will last in a similar way to those observed in the face-to-face exercise program and will be higher than the household group.

4. Scientific Background

Population aging is a worldwide phenomenon and it is estimated that in the coming decades the number of people over 60 years of age should reach 2 billion (UNITED NATIONS, 2013). The increase in the older adults population results from changes in some health indicators, such as increased life expectancy due to improved health conditions, a fall in fertility and mortality levels, and increased socioeconomic development (VERAS, 2009). In Brazil, the number of older adults grew by 40.3% between 2002 and 2012, reaching 20.5 million older adults, approximately 39 older adults for each group of 100 young people (IBGE, 2010). Projections estimate that for 2040 the Brazilian older

adults population will be 23.8%, a proportion of almost 153 older adults per 100 young people (MIRANDA; Mendes, MENDES, SILVA, 2016).

Aging is a process characterized by morphological, physiological, biochemical, and psychological changes that lead to a decrease in the individual's ability to adapt to the environment (SPIRDUSO, 2005). These changes lead to declines in physical function, increased risk of physical disability, and loss of functional independence (BRADY; STRAIGHT; EVANS, 2014), which together lead the older adults to develop a higher risk of hospitalization, institutionalization, falls and death (TIELAND; TROUWBORST; CLARK, 2018).

Some factors can be pointed out as responsible for the decrease in the physical function of the older adults. With aging, there are changes in muscle quantity and quality, translated by increased non-contractile tissue and decreased skeletal muscle mass (LIM et al., 2019; MITCHELL et al., 2012). The decrease in the amount of muscle mass, known as sarcopenia, is a consequence of the decrease in the number of muscle fibers and reduction in the size of the sarcomeres (LEXELL, 2000), and maybe accompanied by the loss of muscle strength, known as dinapenia (CLARK; MANINI, 2010).

In addition to morphological changes, changes in the neuromuscular system also contribute to the decrease in the physical performance of the older adults (TIELAND; TROUWBORST; CLARK, 2018), affecting contractile capacity, increasing the co-activation of antagonist's muscles, and reducing the recruitment and activation timing of motor units (CLARK; MANINI, 2010; NARICI; MAGANARIS, 2006). Together, these factors influence contraction and electrical conduction (FRAGALA; KENNY, KENNY, KENNY, KENNY. KUCHEL, 2015) and can cause reduced mobility, difficulties in performing activities of daily living such as going up and down stairs, sitting and getting up from a chair and, consequently, raising the risk of accidental falls (MANINI, 2012).

In this sense, the regular practice of physical exercises is a strategy to counter the effects of aging. It can prevent or delay the development of chronic diseases and reduce the speed at which physical and physiological changes occur (AMERICAN COLLEGE OF SPORTS MEDICE, 2009; MCPHEE et al., 2016). In addition, physical exercise offers one of the greatest opportunities to prolong years of independent active life, reduce disabilities and improve the quality of life of the older adults (BAUMAN et al., 2016; CRESS et al., 2005).

Physical exercise programs are effective in increasing muscle mass, reducing intramuscular fat infiltration (WU; PARK; MCCORMICK, 2017), increased electrical conduction of muscle, increased strength and muscle power (CADORE et al., 2014; RUBENSTEIN, 2006), speed of gait (RUBENSTEIN, 2006), physical functionality quality of life (LOK; LOK; CANBAZ, 2017). These programs have been applied both in the face-to-face (group) and in the home (individual) modality.

When comparing the two modalities of application, group face-to-face exercise programs seem to be more effective in muscle function, quality of life, and functionality than home programs (LACROIX et al., 2017). In addition, face-to-face exercise programs may offer greater cognitive challenges, due to the need for transportation planning, travel to the place of practice (physical and cognitive demand) and social interaction with the other participants, stimuli that are absent when the exercise is performed at home (STATHI; MCKENNA; FOX, 2010). However, face-to-face programs are associated with difficulties regarding participant's added, due to the lack of access to these types of programs near the residence, difficulties in locomotion, and dependence of family members or third parties for the displacement of the older adults to physical training sites (LACROIX et al., 2017; STATHI; MCKENNA; FOX, 2010).

Alternatively, the literature supports home exercise programs, which reduce these barriers and, once patients are committed to them, have long-term benefits (GILL et al., 2002). In addition, home training can help the individual to improve independent training, being able to sustain him for longer periods and facilitate participation in exercise programs, because they do not need to leave their homes and can adapt the training to their routine (BYRNE et al., 2016; LACROIX et al., 2017). On the other hand, in this exercise modality, supervision is provided indirectly by phone calls or home visits, which may result in limitations regarding the quality of exercise, such as performing exercises with a lower range of motion, less vigorous intensity, and longer pauses, which results in a lower intensity of exercise when compared to face-to-face training (LACROIX et al., 2017).

In this sense, a recent meta-analysis on exercise the effectiveness of home exercise programs found that the greater effectiveness of face-to-face programs could be nullified or decreased with the addition of greater supervision to home programs (LACROIX et al., 2017). Thus, a physical exercise program that combines face-to-face and home sessions

is a valid alternative, because the face-to-face session provides an increase in the social interaction of the older adults and greater supervision of the exercise since the main limitation of home exercise is the lack of control of the frequency, intensity and progression of the exercise (COSTA; Vieira; BENTO, 2019, *IN PRESS*).

A previous study developed in our laboratory tested this model of combined physical exercise program of face-to-face and home sessions in pre-frail older adults women and found that the gains in muscle strength of the lower limbs were similar among those who perform a strictly face-to-face training program (COSTA; Vieira; BENTO, 2019, *IN PRESS*). However, it was not verified which mechanisms were responsible for the improvement of muscle function after the training program. It is known that the mechanisms responsible for increasing muscle strength can be attributed to a combination of neural and morphological factors, such as the increased amount of muscle mass, decreased fat infiltration, increased number of motor units improved firing rate, and decreased co-activation of antagonist muscles (TIELAND; TROUWBORST; CLARK, 2018). As far as we know, no study has verified whether there are differences in neuromuscular and physiological mechanisms responsible for increased muscle strength and functionality in the older adults in different physical exercise modalities (home and/or face-to-face). Knowing the effective contribution of morphological and neural mechanisms on muscle strength and functionality after exercise programs applied in different ways (domicile/face-to-face) is essential for the planning of effective and viable programs for the older adults population, besides expanding therapeutic strategies for the older adults who have difficulty attending the training site.

Thus, it identifies the need to verify the contribution of the neuromuscular system in adaptations to physical exercise in its different forms of application: strictly home, strictly face-to-face, and mixed (face-to-face and home sessions) and the contributions of these adaptations to improve the functionality of the older adults. Thus, this study aims to compare the effects of a multicomponent physical exercise program with different forms of application (face-to-face x household x mixed) and to verify the contribution of the neuromuscular system in adaptations to physical exercise in its different forms of application. The study hypothesizes that the addition of a face-to-face session in a home training program will result in improvements in muscle function, quality, and composition,

gait, functionality, and quality of life of the older adults like a strictly face-to-face physical exercise program and will have a superior effect than the strictly home program.

5. Casuistry

Population aging is a widespread and unprecedented phenomenon in almost all countries, where it is estimated that in the coming decades the number of people over 60 years should reach 2 billion (UNITED NATIONS, 2013). Currently, the older adults corresponds to approximately 30.2 million people, and represent a significant portion of the Brazilian population. In the last five years, the older adults population in the country has increased by 4.8 million, which corresponds to an increase of 18%. In this group, women are a significant majority with 16.9 million (56% of the older adults), while older adults men are 13.3 million (44% of the group).

In the state of Paraná, the older adults population has increased 15.92% in the last five years, totaling 1,717,889 million people (BRAZILIAN INSTITUTE OF GEOGRAPHY AND STATISTICS, 2017). In 2017, Curitiba registered 268,700 people aged over 60 years. In the aging projection scale, the number of older adults in the city of Curitiba is expected to rise to 307.3 thousand in 2020; 432,500 in 2030, and 544,500 in 2040.

6. Material and Methods

6.1 STUDY CHARACTERISTICS

This is an experimental, randomized, simple blind study. In experimental research, the researcher determines an object of study, selects the variables capable of influencing this object, and defines forms of control, observing the effects that the variable produces on the object of study (NELSON; THOMAS, 2012). This study will be approved by the Ethics Committee on Research of the Health Sciences Sector of the Federal University of Paraná.

GPower calculator (MAYR et al., 2007)was used for the sample calculation. An a priori analysis was performed with the following input parameters: effect size (0.25) (COSTA; Vieira; BENTO, 2019, *IN PRESS*), type I error(0.05), type II error (0.80), number

of groups (3), number of measurements (3) and correlation between group (0.5). In addition, a rate of possible losses of 15% was considered. Thus, our analysis revealed a sample size of 42 participants, which will be divided into 3 groups of 14 participants.

6.2 PROCEDURES

The older adults interested in participating in the project will receive detailed information about the objectives and procedures of the research in a meeting scheduled by telephone. The older adults who agree to participate will attend the Center for The Study of Motor Behavior (CECOM) at the Federal University of Paraná for an interview. After this stage, the older adults who meet the inclusion criteria will sign the Free and Informed Consent Form, according to the criteria of the Ethics Committee of the Health Sciences Sector of the Federal University of Paraná, meeting CNS resolution 466/2012 and complementary. Soon after the participants will answer a questionnaire regarding the socio-demographic characteristics, economic classification, cognitive status, risk of fall, and perform a battery of physical evaluations.

After this stage, the older adults will be randomized into three experimental groups: face-to-face exercise group (GP, n=14), home exercise group (GD, n=14), and exercise group with face-to-face and home session (GPD, n=14). Participants in the experimental groups will undergo 12 weeks of training with physical exercise and after the end of the 12 weeks, the groups will be reevaluated and undergo an 8-week follow-up. During this follow-up period, participants will be instructed to maintain their usual activities. The follow-up period will be named the follow-up period. At the end of this period, participants will be reevaluated. Details about the experimental model are presented in Figure 1.

FIGURE 1 - FLOWCHART OF THE PROPOSED EXPERIMENTAL MODEL

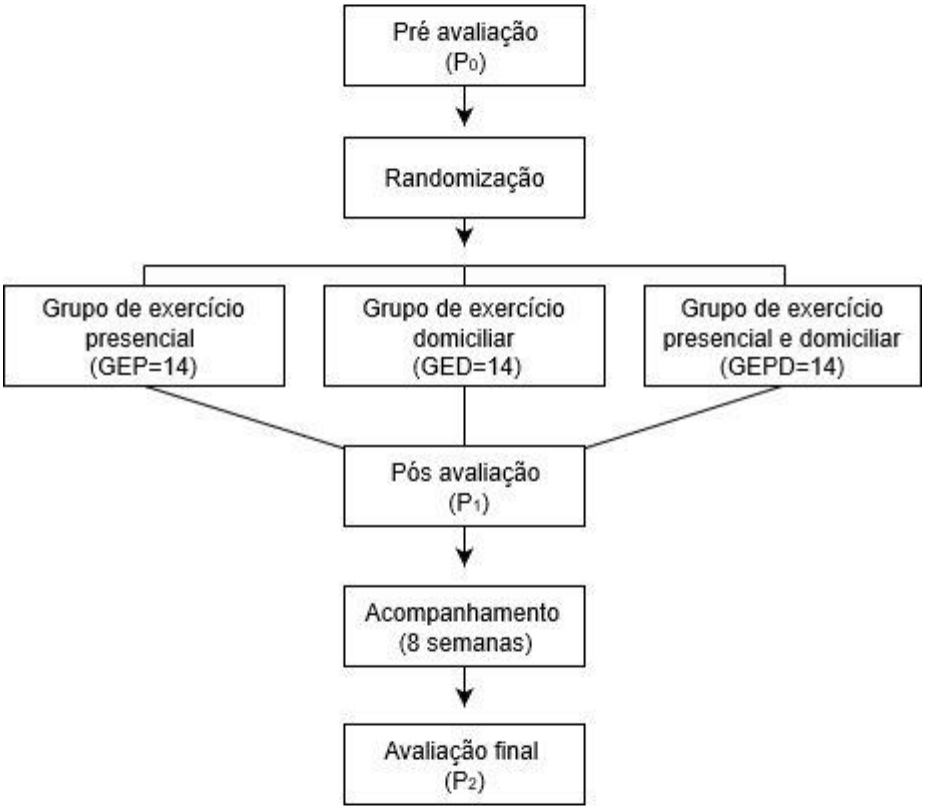

FONTE: Autor (2019).

6.3 EVALUATION PROTOCOLS

The evaluation sessions will be developed in the biomechanics laboratory of the Center for Motor Behavior Studies (CECOM), in the Department of Physical Education of the Federal University of Paraná (UFPR). All evaluations and training protocols will be applied by a team of previously trained evaluators with experience in the protocols used. The evaluators will not participate in the application of the physical exercise program and the applicators of the physical exercise program will not participate in the evaluation, characterizing a simple blind study.

In the pre-evaluation (P0) participants will be submitted to a battery of tests that will be applied in two days, respecting a minimum interval of 48 hours between evaluations. On the first day, participants will respond to a clinical anamnesis, followed by anthropometric evaluation, evaluation of the phenotype of frailty, functional tests of muscle strength, flexibility, and gait speed, and an evaluation of the time-space parameters of gait with and

without a double task. This first evaluation will also be delivered an accelerometer with guidelines for use. On the second day, the participant will be submitted to the evaluation of dynamic balance, flexion strength test, and extension of the hip, knee, and ankle in the isokinetic chair and evaluation of muscle electrical activation and evaluation of muscle architecture by ultrasound.

The primary variables of the study will be neuromuscular function, composed of the evaluation of muscle strength, architecture, and electrical activation of the muscle. The secondary variables will be dynamic balance, functionality, evaluation of the space-time parameters of the usual gait and with double task and quality of life.

All applied protocols will be described in detail below.

6.3.1 Sample characterization

The characterization of the volunteers will be performed through clinical anamnesis, anthropometric evaluation, mental status, and frailty.

The anamnesis will consist of objective questions about schooling, marital status, profession, income, housing, ethnicity, health conditions (vision and hearing), history of surgeries, use of medications, life habits (smoking, alcohol consumption, and history of physical activity), associated diseases and history of falls (APPENDIX 1). To evaluate the history of falls, the volunteer will be asked about the occurrence of falls in the last year through the question: "O/a Sr(a). has had any fall episodes in the last twelve months?" If the response was positive, aspects related to the place of fall, frequency, and its consequences will be questioned.

Anthropometric evaluation will consist of an evaluation of height, body mass, abdominal circumference, and body mass index (BMI).

To assess height, a fixed-wall stadiometer with a 1 mm scale will be used. The volunteer will be instructed to be barefoot and anatomically positioned, with the head and trunk positioned as upright as possible, head oriented parallel to the ground, and with body weight distributed equally on both feet. After proper positioning, the highest point of the head will be used as a reference (GUEDES, 2006).

Body mass will be measured by weighing on a digital scale. The volunteer will be positioned on top of the scale in anatomical position, barefoot and with as light clothes as

possible, with the face directed forward and weight distributed on both feet (GUEDES, 2006).

The measurement of abdominal circumference will be performed with an anthropometric tape with an accuracy of 0.1 cm and with the volunteer in an orthostatic position. The tape will surround the individual at the midpoint between the last rib and the iliac crest (GUEDES, 2006). Three measurements will be performed and the average value will be used.

To obtain BMI, the following calculation will be performed: BMI = body mass (kg)/height(m)², to verify whether they present adequate weight, overweight, or obesity. For the BMI classification, the cut-off points recommended by the Pan American Health Organization (Chart 1), in the Health, Well-being, and Aging (SABE) project that researched the profile of the older adults in Latin American countries (LEBRÃO) will be used. DUARTE, 2003).

TABLE 1 - CLASSIFICATION OF BODY MASS INDEX FOR THE OLDER ADULTS

**ÍNDICE DE MASSA CORPORAL**	**STATUS DO PESO**
> 23,0	Abaixo do peso
23,0 – 27,9	Peso normal
28,0 – 30,0	Pré-obesidade
≥ 30,0	Obesidade

FONTE: ORGANIZAÇÃO PAN-AMERICANA DE SAÚDE (2003).

The cognitive mental status of the older adults will be assessed using the Mini-Mental State Examination (MMSE) (FOLSTEIN; FOLSTEIN; MCHUGH, 1975) (ANNEX 2). The EMME is an instrument divided into two sections. The first requires only vocal responses and covers guidance, memory, and attention with a maximum score of 21 points. The second part evaluates the ability to name, follow verbal and written commands, write a sentence spontaneously, and copy a complex polygon, with the maximum score being 9 points. The maximum total score is 30 points and its cut-off point is defined by the level of education, being 20 points for illiterate, 25 points for 1-4 years of schooling, 26.5 points for 5- 8 years of schooling, 28 points for 9-11 years of schooling and 29 points for individuals with schooling higher than 11 years (BERTOLUCCI et al., 1994). The older

adults who present scores below the cutoff point defined by schooling will be excluded from the study, being considered with cognitive impairment.

To evaluate the phenotype frailty will be used the criteria proposed by Fried et al (2001), these being weight loss, weakness, exhaustion, decreased gait speed, and level of physical activity (APPENDIX 3). Older adults who fall within one or two of the above criteria will be considered pre-frail and those who fall into three or more will be considered frail. Details on the following criteria.

The weight loss criterion is scored when the older adults self reports unintentional weight loss of 4.5 kg or ≥ 5% of the weight in the previous year. The older adults person will be asked the question: "In the last year, you have lost more than 4.5 kg involuntarily (i.e., not due to diet or exercise) " (FRIED et al., 2001).

The weakness criterion will be scored by those who have handgrip strength below 20% of the baseline, adjusted by body mass index and stratified by sex, being the cutoff point presented in Chart 2. The strength of the upper limbs will be evaluated using the SH handgrip dynamometer with the same JAMAR specifications, with a measurement scale ranging from 0 to 100 kg strength (kgf). The test will be performed with the older adults in a sitting position, with the shoulder slightly adduced, elbow positioned at 90° flexion, and forearm and wrist in the neutral position. After positioning, three maximum grip movements will be performed, with one minute of recovery interval, using the mean of the three attempts (FERNANDES; MARINS, 2011).

QUADRO 2 – CUTTING POINT HANDGRIP FORCE

Grip strength, stratified by sex and body mass index (BMI)			
**WOMEN**	**Ponto de corte**	**MEN**	**Cut-off point**
BMI ≤ 23	Grip strength ≤ 17	IMC ≤ 24	Grip strength ≤ 29
BMI 23.1-26	Grip strength ≤ 17.3	IMC 24.1-26	Grip strength ≤ 30
BMI 26.1-29	Grip strength ≤ 18	IMC 26.1-29	Grip strength ≤ 30
BMI > 29	Grip strength ≤ 21	IMC > 29	Grip strength ≤ 32

FONTE: Adapted (FRIED et al., 2001).

The exhaustion criterion will be evaluated using two questions taken from the depression scale of the Center for Epidemiological Studies (RADLOFF, 1977). Volunteers will be asked about exhaustion in the last month with the questions: "Did you feel that you

had to make an effort to do regular tasks?" and "Can you take your things further?". The following answer options will be given: (a) rarely or never, (b) sometimes (1-2 days) or, (c) most of the time or always (3 to 4 days). The volunteer who responds to alternatives b and c (FRIED et al., 2001)will be scored in the criterion.

The decrease in gait speed will be verified through the time spent in seconds to travel a distance of 4 meters. The volunteer will travel, in a straight line, a course of 8 meters, with cones demarcating the starting and final point. The time traveled in the intermediate four meters will be timed. The volunteer will be instructed to walk from one cone to another at his usual speed, "as if he were walking normally on the street" (GURALNIK; et al., 1994). For the criterion score, the cut-off point adjusted for gender and height will be adopted. Women: height ≤ 159 cm ≥ 7 seconds and height > 159 cm ≥ 6 seconds and men height ≤ 173 cm ≥ 7 seconds and height > 173 cm ≥ 6 seconds (FRIED et al., 2001).

The level of physical activity will be verified through the Minnesota Leisure Time Activity Questionnaire (TAYLOR et al., 1978). The Minnesota Leisure Time Activity Questionnaire is an instrument that assesses the level of physical activity, sports, and leisure according to energy expenditure, being adapted and validated for the Brazilian population (LUSTOSA et al., 2011). The weekly energy expenditure will be calculated by obtaining the average energy expenditure of the last two weeks. The criterion will be scored if the one who had the energy expenditure per week lower than 383 kcal for men and 270 for women.

6.4.2 Evaluation of Neuromuscular Function

The evaluation of neuromuscular function will be composed of the evaluation of muscle function, electrical activity, and muscle quality.

6.4.2.1 Muscle Function Assessment

Muscle function will be evaluated using the Biodex Multi-joint System (Biodex Medical Systems) dynamometer. Inc. Shirley. NY. USES). Three maxims of concentric torques of the extensor muscle groups and knee and hip flexors and dorsi and ankle plantiflexors of the dominant limb will be acquired at speeds of 60 and 180°/s. The dominant member will

be defined by asking the participants, "if you were to kick a ball, which foot would you kick?" (PETERS, 1988).

Each test will be performed twice, in the same evaluation session, differentiated by the verbal command of the evaluator: first with submaximally series for familiarization and after, with maximum series for registration.

For familiarization, the evaluator will explain the test and demonstrate the movement to be performed passively and in sequence, flexion and active extension movements will be requested. The protocol for recording will consist of three maximum repetitions of concentric torques of the muscle groups of the knee and hip extensors and flexors and plantiflexors and dorsiflexion of the ankle of the dominant limb at speeds of 60 and 180°/s, with an interval of 1 minute between tests. These muscle groups were chosen for their activation in usual functional activities, such as walking, going up and down stairs, and sitting and getting up from the chair (BYRNE et al., 2016). The velocities of 60 and 180°/s were chosen because they analyzed the strength and power of the lower limbs, respectively. During the test, the evaluators will verbally encourage the older adults to perform as much force as possible, with the phrases "faster" and "stronger".

Participants will be positioned according to Biodex factory specifications, which are described by Symons et al (2004) and will be presented below.

For the evaluation of the knee, the volunteer will be positioned comfortably in the chair of the equipment, with the backrest tilted to 85º and stabilized by belts on the trunk, crossing the hip, around one of the thighs, and around the ankle of the limb to be tested just above the medial maleolo. The chair will be positioned so that the lateral epicondyle of the evaluated knee is aligned with the axis of rotation of the equipment arm (ROAD, 2000). Measurements of chair height, backrest tilt, dynamometer height, rotation of the chair and dynamometer, positioning of the chair and dynamometer, and length of the resistance arm will be noted. These measures will be noted to standardize the test position of each participant individually and to ensure the same position in the revaluation (APPENDIX 4).

For the evaluation of the hip, the volunteer will be positioned comfortably in the chair of the equipment, with the backrest inclined to 0º and stabilized by belts on the trunk, crossing the hip and around the thigh of the limb to be tested just above the knee. The chair will be positioned in such a way that the femur trochanter of the evaluated limb is aligned with the rotation axis of the equipment arm (ROAD, 2000).

For the evaluation of the ankle, the participants will be positioned with the knee in flexion of 30° and with the back of the seat inclined at an angle of 40 °. An arm will be fixed under the thigh for stabilization. The stabilization of the limb will be performed by a band at the waist and thigh. The participant's foot will be positioned so that the ankle rotation axis is aligned with the rotation axis of the dynamometer. The foot will be fixed on a platform with two loops, one distal to the ankle and the other on the metatarsus bones.

Signal acquisition is performed at a frequency of 1000 Hz and all tests will be analyzed using Biodex system 3 advantage software, version 3.2. The following variables will be analyzed:

- Torque Peak (PT), obtained by the highest value of extension torque and flexion of a given curve, expressed in N.m;
- Torque peak normalized by body mass (PT/MC), obtained by dividing the peak torque by body mass, expressed as % of body mass;
- Average power (POT), which represents the speed at which the extensor and flexor muscles of the knee can perform work, expressed in Watts;
- Total work (TT), which consists of calculating the area of extension and flexion curves, is expressed in Joules.
- Maximum repetition of total work (MRTT), which represents the repetition with the greatest amount of work, is expressed in foot-pounds.

6.4.2.2 Neuromuscular function and level of voluntary activation

To evaluate the neuromuscular function, the noninvasive surface electromyography technique (EMGs) will be used, which uses electrodes placed on the skin to record muscle electrical stimuli. Electromyography is an excellent noninvasive method for the evaluation of neuromuscular function, as it can verify changes in muscle, spinal and supraspinal levels (HERMENS et al., 2000). In addition, this method is widely used to verify the effect of physical training on older adults (KNIGHT; KAMEN, 2001; WEI; NG, 2018)

To determine the level of voluntary activation (RS), the twitch interpolation technique (ITT) will be used. In it, the extra torque induced by stimulating the nerve is compared to a control contraction produced by identical nerve stimulation with the relaxed muscle. Thus, the SVAL provides an index of the ability of the central nervous system to

activate the muscles to the maximum at work. The effect induced by electrical stimulation reflects incomplete recruitment of motor units and/or a submaximal firing frequency of motor units and, therefore, a deficit in voluntary activation (ROZAND et al., 2015)

Electrical stimulation consists of inducing an electrical stimulus in a motor nerve to evoke a muscular response. The mechanical response (torque) and the electrophysiological response (electromyographic activity) are recorded simultaneously. The protocol of the present study will stimulate the posterior tibial nerve, which activates plantar flexor muscles (sural triceps and gastrocnemius) (ROZAND et al., 2015). The choice of this protocol is justified by the fact that these muscles are involved in locomotion (BYRNE et al., 2016).

The direct electrophysiological response - M wave, and the indirect response - H-reflex - will be analyzed. The mechanical response - contraction torque, will be quantified by the isokinetic Biodex multi-joint system dynamometer. The M wave and contraction torque reflect neuromuscular transmission and the relationship between muscle excitation-contraction, while the H reflex provides an excitability index at the spinal level. Emg activity and mechanical responses (over imposed contraction) will be recorded during maximal voluntary contractions to assess the level of voluntary activation (SNA). The SVAL provides an index of the ability of the central nervous system to activate the active muscles to the maximum.

The Trigno Wireless electromyograph, Delsys, USA, will be used to acquire EMG signals; using a neuro IOM - neurosoft ® with AMBU Neuroline 715 surface electrodes. The muscle analyzed will be the sural triceps of the dominant limb, as it is a muscle widely requested in daily activities. The dominant member will be defined by asking the participants, "if you were to kick a ball, which foot would you kick?" (PETERS, 1988). Bipolar electrodes with metallic surfaces responsible for acquiring electromyographic data will be used. Before placing the electrode, the skin will be scraped, lightly rubbed with abrasive gel, and cleaned with cotton soaked in alcohol, as a way to avoid possible interference sonographic signal (HERMENS et al., 2000). After cleaning, the electrodes will be fixed to the skin using disposable double-sided adhesive tape, to minimize possible displacement of the electrodes. For the oil muscle, the electrode will be positioned 2/3 of the line between the medial condyle of the femur and the medial condyle of the maleole; for the medial gastrocnemius, in the most prominent bulge of the muscle; for the lateral

gastrocnemius, 1/3 of the distance along a line between the head of the fibula and the heel; and for the anterior tibial, 1/3 of the distance along a line between the tip of the fibula and the tip of the medial maleolo, towards the muscle fibers according to the recommendations proposed by SENIAM (Surface EMG for the Non-Invasive Assessment of Muscles). A reference electrode will also be fixed in a central position on the same leg (between the stimulation and recording sites).

As the mechanical response induced by tibial nerve stimulation is generated by plantar flexors and eventually reduced by a concomitant activation of the anterior tibial muscle (AT), electromyographic activity of the antagonist's muscle and palpation of the muscle/tendon will allow the exclusion of THE activation.

In addition, an electrode will be positioned for electrical stimulation on the patellar tendon. The best site for posterior tibial nerve stimulation will be defined after a series of tests until a maximum value is reached, where the threshold will be positioned in the popliteal fossa. The EMG activity of the anterior tibial will be recorded to ensure that the fibular nerve is not activated to avoid the influence of antagonists. The width of the pulse will be 1 msec to provide an optimal activation of nerve fibers, especially aferening fibers.

After placing the electrodes, the participant will be positioned in the Biodex System isokinetic dynamometer, according to the standardization suggested by the manufacturer for the evaluation of the ankle, which has been described previously (for more information check item 4.4.1.1). The participant's foot will be securely fastened and will exert pressure on a platform attached to the ergometer to record the torque of the plantar flexor.

The sampling rate for torque and EMG measurements will be set to 2-5 kHz. The EMG signal will be recorded using an analog-to-digital (AD) conversion system. The signal will be displayed on a monitor with a data acquisition system, which will instantly provide values of various parameters (maximum value, peak amplitude to peak, duration). EMG signals will be differentially amplified with a gain of 2000 and a bandwidth of 10-500 Hz to -3 dB. The actual EMG activity will be recorded with a sampling frequency of 2000 Hz. A window of 500 msec will be used to calculate the values of the mean square of the EMG root (RMS), around the time of maximum isometric flexion of the ankle.

The collection will be performed during maximum voluntary isometric contraction. The protocol of maximum voluntary isometric contraction (MVIC) will consist of three sets with five seconds each duration. The rest interval between each series will be 120 seconds.

Participants will be instructed to perform the contraction with "the greatest amount of force and as soon as possible". If an initial counter-movement is observed (identified by the visual drop in the torque signal), the attempt is discarded and a retry is performed. During all MVIC series, participants will be able to view their torque curves on the dynamometer monitor as visual feedback, in the same way, that they will be verbally motivated to try to get their best performance (DOTAN; MITCHELL, 2013). The highest force value achieved in the three maximum efforts will be taken as the MVIC. The variability of 10% between the measurements will be determined and the highest value will be used for analysis.

To verify the level of voluntary activation, two supramaximal doublets (two electrical stimuli with 200 μs pulse width, the inter-stimulus interval of 10 ms, and a maximum output of 1000 mA) will be applied pericutaneamente to the femoral nerve during a maximum voluntary isometric contraction (MVIC), using an electrical stimulator model Neuro IOM - Neurosoft® with surface electrodes AMBU Neuroline 715.

The supramaximal stimulus will be administered at 350-500 milliseconds after the beginning of the MVIC plateau (overlap/overlap contraction) and again 3-5 seconds later, in rest condition (enhanced contraction). The stimulus intensity will be determined by the administration of progressively increasing electrical stimuli. The stimuli will be performed with participants at rest, from 50 mA, increasing 5 mA until reaching maximum twitch torque. Then, an additional 30% will be used to ensure maximum stimulation.

The variables analyzed will be the peak of twitch torque (PT); contraction time (CT); torque development rate (RTD = PT/CT); half-relaxation time and the VAL. The % VAL will be calculated using the equation: [1 – (superimposed doublet torque/rest doublet torque)] x 100.

MVIC attempts will be rejected and repeated when the torque line does not exhibit a light plateau before over imposed stimulation and when the stimulus is released before the MVIC.

6.4.2.3 Muscle Architecture Assessment

The muscle architecture of the vastus lateralis (VL) and medial gastrocnemius (MG) muscles will be evaluated through ultrasound equipment (Konica Minolta Medical Imaging Inc Newark-Pompton Turnpike, Wayne, NJ, USA) in mode B with linear arrangement

transducer (4 cm height x 2 cm length, 10 MHz), with collection depth adjusted to 5 cm for VL and 4 cm for MG. The dominant limb will be evaluated, and the transducer will be coated with water-based transmission gel with sufficient quantity to ensure that clear images of the muscle are obtained, without the need to compress the muscles during collection.

Participants will be instructed not to perform any type of physical exercise of lower limbs 48 hours before the imaging procedure. Before collection, participants should rest for 20 min in the supine position, with the evaluated limb extended and relaxed, to allow accommodation of body fluids (BERG; TEDNER; TESCH, 1993). During the measures, participants will be instructed to relax the member as much as possible.

To evaluate the vastus lateralis muscle, the images will be obtained at 39% of the leg length, defined from the distance between the patella and the iliac crest (Blazevich et al. 2007). The proximal insertion of the VL muscle will be identified and marked, with axial sections marked at intervals of 30. The transducer will be oriented in the axial plane, aligned perpendicularly to the VL muscle, and moved from the center to the lateral position along with a demarcated jig over the skin.

To evaluate the medial gastrocnemius muscle, the participant will be lying in the ventral decubitus on a stretcher, with the feet hanging on the edge and an angle of the ankle joint will be fixed at 15º dorsiflexion. The ultrasound transducer will be positioned in the 30% part between the lateral maleolo of the fibula and the lateral condyle of the tibia. These specifications were chosen for the evaluation because previous studies have raised minimal curvature of the fascicle in this place when the participants were lying in this position (SELVA RAJ; Bird; SHIELD, 2017). The transducer will be oriented in the axial plane, aligned perpendicularly to the muscle, and moved from the center to the lateral position along with a demarcated jig over the skin.

The images will be reconstructed using PowerPoint (Microsoft, Redmond, WA, USA), following the descriptor procedures by Reeves et al (2004) (REEVES; MAGANARIS; NARICI, 2004). Cada image will be positioned manually until the fascia of the muscle is reconstructed. The VL cross-section area will be measured using computerized planimetry, where it will be used with the aid of the mouse.

The images will be analyzed using ImageJ software (version 1.46). The software will be calibrated from a known distance of 1cm in the images taken by the ultrasound equipment

measurement tool. The intensity of the echo, the length of the fascicle, the angle of pity, and muscle thickness will be taken into account. These variables were chosen because the length of the muscle fascicle plays a role in the generation of strength during contractions, while the angle of pity of the fascicle and muscle thickness are important factors for the generation of global strength (BLAZEVICH; SHARP, 2005).

The thickness of the vastus lateral is to be defined as the distance between superficial aponeurosis and the femur. The thickness of the medial gastrocnemius muscle will be defined as the distance between superficial and deep aponeurosis. The angle of pity will be measured as the angle between the fascicles of the muscle and deep aponeurosis. The length of the fascicle will be measured as the length of a fascicle between its insertions in superficial and deep aponeurosis. In cases where the fascicles are length beyond the recorded image, the length of the fascicle, the thickness, and the angle of plucking will be estimated using the following equation (E5B; KUMAGAI; BRECHUE, 2000):

$$\text{Fascicle length} = \text{thickness of sin muscle } \theta^{-1}$$

where θ is the angle of pity of the muscular fascicle determined by ultrasound.

The echo intensity was determined by the analysis of the grayscale, using the standard histogram function in the Software Imagem-J (National Institute of Health, USA, version 1.37). The pixels within the area of interest will be rendered with the Fourier quick transform, resulting in a distribution of 256 shades of gray, being 0 = black and 255 = white. Lighter pixels (hyperalcoholic) may indicate the presence of infiltrated fat and non-contractile elements (YOUNG, HUI; JENKINS, NATHAN T.; ZHAO QUN, 2015). The echo intensity will be calculated as the mean and median of the values within the cross-section area, and the higher the mean and median, the greater the presence of infiltrated fat and non-contractile elements in the muscle.

The reproducibility of the measurements (mean and median) will be determined by calculating the values of the coefficient of variation (CV), intraclass correlation coefficient (ICC), and typical measurement error (TMS) between the images collected on 2 different days, with at least 48 hours of the interval between them. As suggested by Hopkins (2000), The TMS is the result of the ratio between the standard deviation of the difference

of repeated measurements on two consecutive days (day 1 and day 2) and the square root of two (HOPKINS, 2000).

It is known that the ideal CV should be the lowest possible, preferably equal to or below 10%, the ICC values greater than 0.9 represent high reliability and between 0.7 to 0.8, reasonable, the TMS uses the unit of measurement of the analyzed variable and its minimum value (closer to zero) represents a small variability between the measurements (ATKINSON; NEVILL, 1998).

6.4.2 Gati analysis

The gait will be evaluated using the ProtoKinetics Zeno walkway (ProtoKinetics LLC, Havertown, Pennsylvania) carpet, which consists of an instrumented walkway with 16 levels of pressure sensors distributed over 6.09 m long and 0.61 meters wide. The walkway has three layers: protection base, pressure sensors, and protective cover, which detects and collects pressure data in gait evaluations. The instrumented walkway is connected to a laptop by a serial interface cable to process and store data using ProtoKinetics Movement Analysis Software. The Zeno walkway has been used to investigate space-time characteristics of gait in older adults (DALY et al., 2015; MCBEAN et al., 2016) and has excellent competing validity (VALLABHAJOSULA et al., 2017).

The evaluation protocol will consist of four different conditions: walking at the usual speed, maximum speed, usual speed with the double task, and maximum speed with the double task. Volunteers will travel a distance of 10 meters, where the first and last two will be considered acceleration and deceleration time. For the usual speed, participants will be instructed to walk at the speed they walk in the street, through the command: "You will make the route at the speed you normally walk on the street". For maximum speed, participants will be instructed to walk as fast as possible, through the command: "You will walk as fast as possible as if you were missing the bus". In addition, the maximum speed will be considered to be those with at least a 10% decrease in time to the usual gait.

The double task gait will be evaluated using an arithmetic cognitive task (countdown from 50). This task was chosen because it is the most used in studies with older adults and because an arithmetic task depends essentially on operational or working memory, temporary storage, an information processing system, which is directly related to executive

functions (GOMES et al., 2016). For the evaluation, participants will be instructed to walk and perform the countdown aloud from number 50. The importance of walking and counting at the same time, at the best of your ability, will be emphasized, without prioritizing any task. Possible counting errors will not be corrected (BEAUCHET et al., 2009).

The following sand-time parameters will be analyzed:

- Gait speed: speed of the participant in the direction of displacement (m/s);
- Cadence: number of steps per unit of time (steps/min);
- Time of the stride: the time of duration of a stride(s);
- Stride length: distance between the initial contact of the analyzed foot and the second contact of the same foot, projected on the displacement axis of the participant (m);
- Stride width: lateral distance between the heels of the consecutive contacts of the two feet (m);
- Simple support time: the time which only one foot is in contact with the ground(s);
- Double support time: the time in which the two feet are in contact with the ground(s);
- Balance time: the time when the foot is in the air for the advancement of the limb(s).

6.4.3 Avaliação do equilíbrio dinâmico

The evaluation of dynamic balance will be performed by performing a video game, with an analysis of the displacement of the center of mass of the participant. For this, two force platforms will be used, the three-dimensional motion analysis system (VICON) and Microsoft Kinect®.

Vicon's motion analysis system® allows the capture of movements in three planes (x, y, z) through eleven infrared radiation cameras, model Vicon® Bonita B10. The cameras have a resolution of one megapixel and an accuracy of up to 0.5 mm, at a volume of 4m x 4m. These cameras record only the reflective spherical markers positioned on specific anatomical points according to the biomechanical model used. The biomechanical model composed of 29 reflective markers will be used, which allows the reconstruction of 9 body segments, which are necessary for the subsequent calculation of the center of mass (CM).

The power platforms that will be used are AMTI® model OR6-7-1000, and will be affixed to the ground 5 mm apart and leveled in height with a wooden table. The platforms

will be centered within the capture area of the Vicon system cameras® and a projection screen of the Microsoft Kinect ® equipment will be positioned 2 m away.

Before the beginning of the collection, 29 reflective markers of 14 mm diameter will be placed bilaterally positioned on the following anatomical points: the anterior face of the head, the anterosuperior face of the head, acromial, lateral epicondyle of the humerus, syroid process of the ulna, the distal phalanx of the middle finger, jugular notch, anterosuperior iliac spine, sacrum, larger femoral circumference, lateral epicondyle of the femur, tibial circumference, lateral maleolo, calcaneus and head of the 2nd metatarhomeus.

After placing the markers, volunteers will be led to the capture area of the Vicon system cameras® and positioned on the power platforms.

The game used for the collection is called by the acronym SIRMeC, which stands for Interactive System of Motor and Cognitive Rehabilitation. This system was developed in conjunction with engineers, physiotherapists, and a student of the Digital Game Development Technology course at PUCPR, based on physiotherapeutic exercises of neurological rehabilitation, often used in clinical practice (LASKOS, 2014). Serious Game SIRMeC is designed so that different game parameters are customized according to the particular characteristics of each individual. To define the limits of the challenges launched on the screen, the system performs the calibration of the effective playing area, which allows recording the maximum amplitude measurements of the movements involved in each game, previously. This is done through Microsoft Kinect equipment® (Microsoft, Redmond, USA).

The game held in the collection will be skiing. The game consists of a winter sports environment, in which the volunteer must pass inside flags contained in the track, controlling the direction of his ski by performing the trunk rotation movements. The challenges present in the game are random and on both sides of the track, favoring the training of rotation and trunk dissociation. The game has different levels of speed, being moderate, fast, and very fast. The volunteer will remain at each game level for 60 seconds, totaling 180 seconds per game.

After processing and analysis of the data, the following variables will be used: support base area, stability margin, the distance between the center of mass and the boundaries of the support base, and weight discharge symmetry.

6.4.4 Funcionalidade

The functionality of the participants will be evaluated through the short physical performance battery test battery (SPPB) and the Rick and Jones test battery (APPENDIX 5), to verify muscle strength, agility, and dynamic and static balance.

The Short Physical Performance Battery (SPPB) is composed of a balance test, 4-meter walk, and chair lift and sit test five times. For the balance test, the participant must be able to remain in each of the three positions for 10 seconds: standing with his feet together, standing with his feet in a semi-tandem position, and standing in a tandem position. In the first two positions, the participant receives grade 1 if he can stay in position for 10 seconds, and note 0 if he cannot hold on for 10 seconds. In the third position, the individual receives a score of 2 if he/she can maintain the position for 10 seconds; note 1 if you hold the position for 3 to 9.99 seconds and note 0 for the time less than 3 seconds or if you do not perform the test (GURALNIK; et al., 1994).

In the gait speed test, the participant must walk in a usual step a distance of 4 meters, demarcated by tapes fixed to the ground. Note 0 is assigned to the participant who cannot complete the test, note 1 if the time is longer than 8.7 seconds, note 2 the time is 6.21 to 8.7 seconds, note 3 if the time is 4.82 to 6.2 seconds, and note 4 if the time is less than 4.82 seconds (GURALNIK; et al., 1994).

For the test of getting up and sitting in the chair, the patient is asked to start the test the sitting position, with his arms crossed on the trunk and, at the sign of the evaluator, should get up and sit in the chair as soon as possible, five times. If the participant cannot stand up 5 times or complete the test in a time greater than 60 seconds, 0 points are assigned; if the test time is 16.7 sec or more, 1 point is assigned; if the test time is from 13.7 to 16.69 sec, 2 points are awarded; if the test time is 11.2 to 13, 69 sec, 3 points are awarded; and finally, test time less than 11.19 sec: 4 points (GURALNIK; et al., 1994).

The Final Score of the SPPB is given by the sum of the three tests, with a maximum score of 12 points. You will be given the following rating according to the score: 0 to 3 points: disability or poor capacity; 4 to 6 points: low capacity; 7 to 9 points: moderate capacity and 10 to 12 points: good capacity (GURALNIK; et al., 1994).

Rikli and Jones's Senior Fitness Test (SFT) battery (2001) consists of the chair lift and sit tests, forearm flexion test, sit-and-reach test, Timed-up-and-Go test, back-to-back reach test, and 6-minute walk.

The forearm flexion test assesses the strength and strength of the upper limb. The participant starts the test in the sitting position and holds a halter (2kg for women and 4 kg for men) with the arm extended near the chair and perpendicular to the floor. At the signal, the participant turns his palm up while flexing the arm in a full range of motion and then returns the arm to a fully extended position. In the initial position, the weight should return to the handshake grip position. The evaluated is encouraged to perform as many repetitions as possible in 30 seconds. The total number of correct push-ups performed within 30 seconds will be noted (RIKLI; JONES, 2011).

The sitting and reaching test assesses the flexibility of the lower limbs. The participant will be instructed to touch the chair only the gluteus, keep a flexed leg, with the foot on the ground, the knees parallel, facing forward. The other loss should be extended to the front of the hip, with the heel on the ground and plantar dorsiflexion at approximately 90º. With the leg extended, the participant should slowly lean forward, keeping the spine as upright as possible and the head aligned to the spine. The participant will try to touch the toes by slipping the hands, one on top of the other, with the tips of the middle fingers, on the extended leg. For the score, the distance (cm) to the toes or the distance that can be reached beyond the toes (RIKLI) will be recorded; JONES, 2011).

The Timed Up-and-Go (TUG) test assesses functional mobility and dynamic balance. The protocol consists of the volunteer getting up from a chair (approximately 46 cm high), walking to a line on the floor 2.44 meters away, turning, returning along the same route, and sitting in the chair again, at a comfortable and safe place. The subject should start the test with the trunk resting on the back of the chair and at the end, pull over again, and the time is timed from the verbal command "already" until the moment the evaluated one re-supports the trunk in the chair. A familiarization will be performed and after the time in seconds spent to complete the test will be recorded from the verbal command "already" and finalized when the volunteer supports the trunk in the chair (PODSIADLO; RICHARDSON, 1991).

The test reaching behind the back assesses the flexibility of the upper limbs. Standing, the participant places the hand preferably on the same shoulder, with the palm open and

fingers extended, reaching the middle of the back as much as possible. The hand of the other arm will be placed behind the back and will attempt to touch or overlap the outstretched middle fingers of both hands. For the score, the distance from the overlap will be recorded, or the distance between the tips of the middle fingers. Negative results (-) represent the shortest distance between the middle fingers; positive results (+) represent the measure of middle-toe overlap (RIKLI; JONES, 2011).

Finally, a 6-minute walk test will be performed that assesses the cardiorespiratory capacity of the individual. The participant will be instructed to walk, for 6 minutes, the longest possible distance, on flat terrain, at the usual speed. The 6-minute walk test uses a 45.7 m course measured within 4.57 m segments. At the end of 6 minutes, the evaluator measured the total distance traveled (AMERICAN THORACIC SOCIETY, 2002; RIKLI; JONES, 2011).

6.4.5 Quality of life

To assess the quality of life of the participants, the Medical Outcomes Study 36 – Item Short-Form Health Survey (SF-36) questionnaire (APPENDIX 6) will be used to assess the quality of life of the participants (APPENDIX 6). The SF-36 is a generic instrument for assessing the quality of life, easy administration, and understanding. It was developed by Ware and Sherbourne (1992) and translated and validated into Portuguese by Ciconelli (1999), and can be self-applied, applied via computer, or applied by a previously trained coach. The instrument is multidimensional consisting of 36 items, encompassed in 8 components: functional capacity, physical aspects, pain, general health status, vitality, social aspects, emotional aspects, mental health, and a comparative question about the current perception of health. Each component receives a score, ranging from 0 to 100, with 0 worse perceptions of quality of life and 100 better perception of quality of life (CICONELLI et al., 1999; WARE; GANDEK, 1998).

6.4.6 Assessment of physical activity level

To assess the level of initial physical activity and during the exercise program will be used the accelerometer of the brand *Actigraph,* GT3X MODEL. The accelerometer will be used to certify the performance of home training, given the limitations in controlling the performance of sessions at home only by the exercise guide calendar. In addition, it will be used to compare the percentage of time spent in sedentary, mild, moderate, vigorous, and very vigorous activities in strictly face-to-face and strictly home sessions.

Participants will be instructed to use the appliance for 7 consecutive days, all day, removing only for sleep and for carrying out aquatic activities, including bathing. The appliance will be attached to an elastic strap and positioned at the ankle of the dominant leg, just above the malleolus. The use of the accelerometer for at least 4 days will be considered as valid data, one of which is the weekend. The day will be considered valid when a minimum of 10 hours of recording is recorded. The data will be collected at a frequency of 60 Hz. The *process of downloading and analyzing* registered data will be carried out *by the Software* Actilife (HENDELMAN et al., 2000).

In the software, *the count's values* are translated into minutes of physical activity. The intensity of physical activity practice will be analyzed from the classification established by the software *that* uses the Freedson equation, being: sedentary activity (0 - 99 counts/min), mild (100 - 1951 counts/min), moderate (1952 - 5724 counts/min), vigorous (5725 – 9498 counts/min) and very vigorous (>9499 counts/min) (HENDELMAN et al., 2000).

The following variables will be analyzed: average calories per day, time in sedentary activities, light activities, moderate activities, vigorous and very vigorous activities.

6.5 EXERCISE PORGRAM

Volunteers will participate in a multicomponent exercise program, combining group activities in person and at home over a period of 12 weeks, with three weekly sessions and a duration of 60 minutes, totaling 180 minutes per week. The face-to-face group (GEP) will hold all training sessions at DEF/UFPR, the homegroup (GED) will hold all training sessions at home and the program group with face-to-face and home sessions (GEPD) will hold a weekly session at DEF/UFPR and two weekly sessions at home.

The exercise program will consist of functional exercises of muscle strength, balance, flexibility, and gait, based on the recommendations of Singh (2002) and ACSM (2009), as shown in Chart 3. Each session will be divided into 05 minutes of dynamic and joint warm-up, with large-amplitude exercises such as abduction, adduction and rotation of the upper limbs, lateral rotation of the trunk, abduction, and adduction of the lower limbs, flexion, and extension of the hip; followed by 10 minutes of specific exercise of dynamic and static balance, with manipulation of visual information (open and closed eyes), reduction of the support base, stationary and displacement gait; followed by 25 minutes of muscle-strengthening exercises of the lower limbs; 10 minutes for specific gait exercises; and 10 minutes of flexibility activities, with relaxation and stretching of the large muscle groups of the upper, lower limbs and trunk.

QUADRO 3 – *PLANNING THE FISCAL YEAR PROGRAM*

	**Balance training**	**Strength training**	**Gait training**	**Flexibility training**
**Volume**	1–2 sets<br>4 to 5 exercises	3 sets<br>8-12 repetitions<br>5-6 exercises involving the largest muscle groups	1 set<br>5 repetitions<br>6 exercises involving gait and dual-task	Main muscle groups<br>Sustain the 20s each
**Intensity**	Progressive difficulty with increased compressibility reduced visual information and manipulation of objects	15–17 on the Borg Scale<br>1 min rest between sets	15–17 on the Borg Scale<br>1 min rest between sets	Progressive neuromuscular facilitation technique

FONTE: O Autor (2019).

Balance exercises will be composed of different exercises emphasizing dynamic postures, with daily movements or that cause balance disorders. Static exercises will consist of unipodal position, semi tandem position, tandem position (luxus of the foot that is behind touching the calcaneus of the front foot), stand on the heels. The maximum time in each static position will be 30 seconds. Dynamic exercises will consist of forward, backward and lateral shifts in planti and dorsi bending and tandem position, transfers from one chair to another, going over objects, climbing up and down steps slowly, and rotating.

The intensity will be increased with the reduction of the support base (feet); reduction of sensory information (visual and vestibular); or disturbance of the center of mass (for example, holding a heavy object on one side while maintaining balance, standing on one leg while lifting the other leg behind the body, or leaning as much as possible without falling or moving the feet). More information on balance exercises is in Chart 4.

CHART 4 - PLANEJAMENTO SEMANAL DOS EXERCÍCIOS DE EQUILÍBRIO

**Semana**	**Semana 1-3**	**Semana 4-6**	**Semana 7-9**	**Semana 10-12**
**Volume**	1–2 séries<br>4 a 5 exercícios enfatizando posturas estáticas e dinâmicas			
**Intensidade**	Com redução da base de apoio	Superfície instável (colchonete)	Redução da informação sensorial	Manipulando objetos
**Exercícios**	Exercícios estáticos: posição unipodal; semi tandem; posição tandem e ficar de pé sobre os calcanhares + manipulando objeto (ex: bola – jogando para cima, trocando de mão, jogar e bater palma).<br><br>Exercícios dinâmicos: caminhada para trás; caminhada formato do número 8; caminhada lateral; caminhada dorsiflexão; caminhada plantiflexão; sentar e levantar da cadeira; transferências de uma cadeira para outra; passar por cima de objetos; subir e descer degraus devagar; girar; em pé em uma perna enquanto levanta a outra perna atrás do corpo, ou inclinando-se o máximo possível sem cair ou mover os pés; deslocamento lateral com agachamento/adução e abdução de quadril.			

FONTE: O Autor (2019).

Muscle strength exercises will be composed of exercises that involve the main muscle groups required to perform daily activities, such as walking, lifting and sitting, climbing stairs. Exercises of the dorsiflexion and ankle plantiflexors, knee and hip flexors and

extensors, exercises for glutes, adductors, and hip abductors will be developed. Exercises for the abdomen and upper limbs will complete the training. From 2 to 3 sets from 08 to 12 repetitions will be performed in each exercise with an interval of 1 minute, with intensity controlled by the subjective perception of exertion from the Borg scale (6-20) (APPENDIX 7). The planning of the strength exercise program is presented in Chart 5.

The intensity of the sessions will be measured by the perceived effort rate from the Borg scale (6-20), at different moments of the session in the strength exercises, to characterize the intensity and not as monitoring. The scale will be explained, followed by familiarization. The overload of strength exercises will be determined by performing 8 to 10 repetitions of the exercise, with resistance material (shin), of good quality before fatigue.

QUADRO 5 - PLANEJAMENTO SEMANAL DOS EXERCÍCIOS DE FORÇA

<table>
<tr><th>Semana</th><th>Semana 1-3</th><th>Semana 4-6</th><th>Semana 7-9</th><th>Semana 10-12</th></tr>
<tr><td>Volume</td><td>3 sets of 12 repetitions, 1-minute interval</td><td>3 sets of 10 repetitions, 30-second interval</td><td>3 sets of 8 repetitions, 1-minute interval</td><td>3 sets of 8 repetitions, 30-second interval</td></tr>
<tr><td>Sobrecarga</td><td>Familiarization without overload and onset of load progression</td><td colspan="3">Overload progression: good quality execution before fatigue</td></tr>
<tr><td>Intensidade (PSE)</td><td>(13-15)</td><td>(15–17)</td><td>(15–17)</td><td>(15–17)</td></tr>
<tr><td>Exercícios</td><td colspan="4">Division of sessions by muscle grouping:
Monday with emphasis on the muscles of the quadriceps and calf;
Wednesday with emphasis on the upper limbs and abdomen;
Friday with emphasis on posterior muscles and ductors/abductors of the thigh.
Division of sessions by muscle grouping:
Monday with emphasis on the muscles of the quadriceps and calf;
Wednesday with emphasis on the upper limbs and abdomen;
Friday with emphasis on posterior muscles and ductors/abductors of the thigh.</td></tr>
</table>

FONTE: O autor (2019).

The gait exercises will be composed of displacements at the usual and maximum speed, stationary gait with and without elevation of knees, and activities involving an agility ladder. The agility ladder consists of 10 squares (25 cm each) arranged in a straight line. Volunteers will be instructed to walk from one end of the scale to the other, according to a pattern provided (Figure 2). When volunteers reach the end of the ladder, they will be instructed to return to their starting positions, walking normally off the ladder, then start the next pattern. Forward, backward, lateral, and diagonal step patterns will be included. The progression of the exercise will be carried out through the difficulty of the patterns; by decreasing the support base, such as walking on your toes or with your heels; and performing cognitive tasks simultaneously, such as naming animals and counting down. Each pattern will be repeated 4 to 10 times per session.

Volunteers will be encouraged to focus on successfully performing each pattern of steps. The step cadence will not be determined and can be performed at the preferred rate for each participant. The agility scale exercises were based on the study by Shigematsu et al (2008).

FIGURE 2 - WALKING EXERCISES ON THE AGILITY LADDER

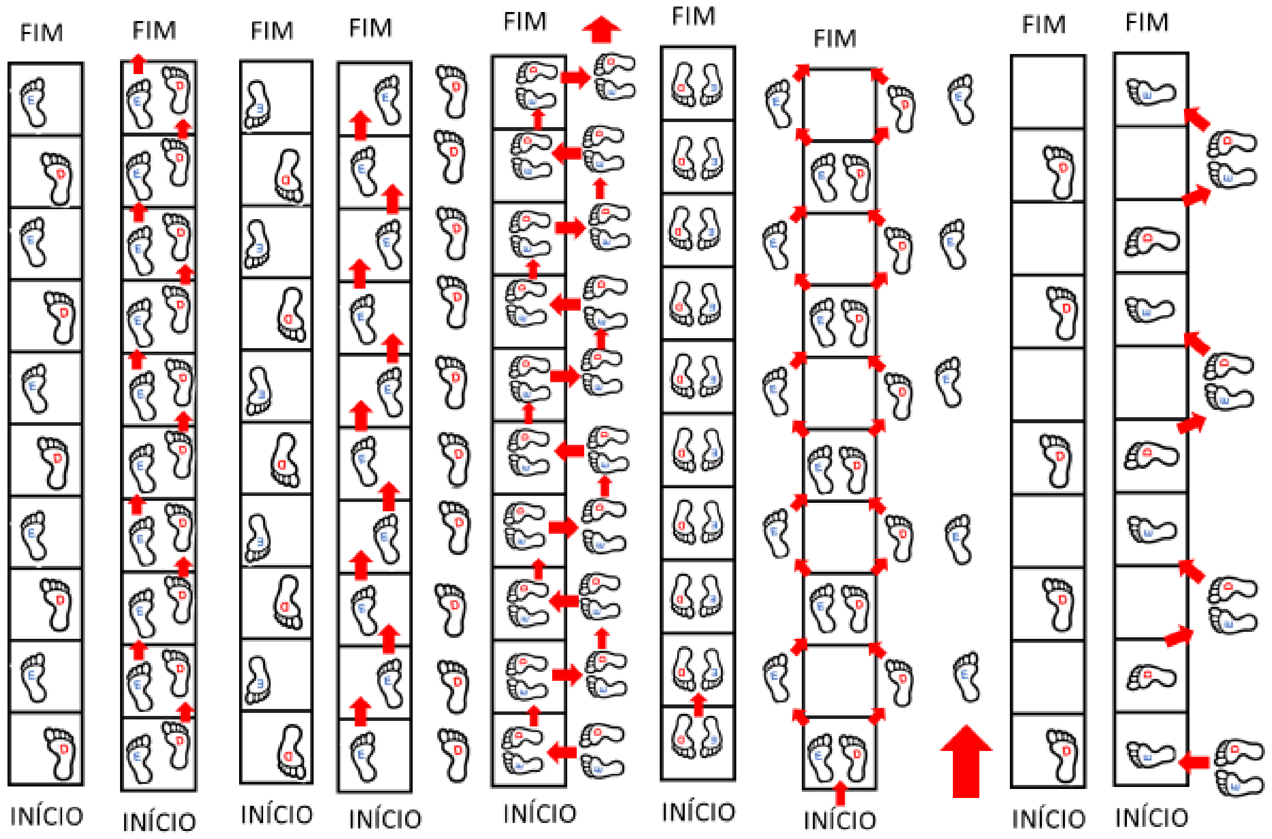

FONTE: O Autor (2019).

Flexibility exercises will be composed of static stretching of the main muscle groups, such as flexion and extension of hip and trunk, abduction, adduction of the upper and lower limbs. Each position will be held for at least 20 seconds. The intensity will be increased by the progressive difficulty of the exercise, as tolerated by the participant and using the principle of the technique of progressive neuromuscular facilitation. Proprioceptive neuromuscular facilitation involves as much muscle stretching as possible, followed by relaxation and an attempt at further stretching, to finally maintain the maximum stretching position for at least 20 seconds (SINGH, 2002).

During all training sessions, the execution of the movements will be prioritized and corrected individually. Volunteers will be encouraged at all times to perform the exercises at the highest possible speed. The intensity of the exercises will be gradually increased as the participants adapt to the stimulus. The exercise program will be divided into four difficulty levels: week 1-3 phase, with a slow introduction of resistance material and balance exercises, were performed with reduction of the support base; week 4-6, with the increase of the load of the resistance material and the balance exercises, were performed

with unipodal support; week 7-9, with increased load and series and balance exercises, were performed with support on unstable surfaces, such as mattresses; week 10-12, with increased load and series and balance exercises, were performed with the reduction of sensory information (visual and vestibular).

Volunteers who will perform home sessions (GEPD group and GED) will receive an initial visit at their home in which they will be informed all the necessary guidelines for carrying out the program safely and a manual with instructions for each exercise, with space for recording the sessions performed and possible occurrences (APPENDIX 8). In addition, they received a kit of materials containing three pairs of shin players (1, 2 and 4 kg) to provide resistance to strengthening exercises and an agility scale for gait exercises. To control adherence to the program, participants will record the days that the program was performed and receive messages with incentives to perform the exercises at home.

6.5 STATISTICS

Descriptive, mean, and standard deviation statistics will be used to analyze the collected data. The Shapiro-Wilk test will be applied to verify the normality of the data and the Levene test for sample homogeneity. For the normal data, the comparison between groups and between the periods (pre and post-training) will be used through the ANOVA of a mixed model with posthoc Bonferroni. When differences were observed between the groups in the pre-training period, a covariance analysis (ANCOVA) will be applied, with the initial values as a covariate, and the data in the post-tests were compared, disregarding the initial differences. The coefficient of $p<0.05$ will be adopted to determine the significance of the data. The tests will be performed using IBM SPSS statistics version 25 software.

7. Expected Results

With the present study, it is expected to find, after the physical exercise program, a better understanding of the conduct of the physical exercise in the older adults population, such as the identification of the proposal to conduct the most effective exercise program to increase muscle strength, functionality, and quality of life of the older adults; the

development of an exercise program that can be performed both at home and in groups, facilitating barriers such as dependence on the older adults to get around the physical training site; the promotion and maintenance of independence in the performance of physical activity, as well as the performance of basic activities of daily living; maintaining an active lifestyle after the completion of the physical exercise program. Additionally, it is expected to identify how long there is a reduction in the effects of exercises on the clinical, functional, and quality of life aspects of participants who stopped exercising after the intervention.

In addition, the project will contribute to the training of researchers, as it will be part of the doctoral thesis of student Sabrine Nayara Costa (PPGED/UFPR) and a research project for scientific initiation of the Physical Education course at UFPR. Additionally, it is expected to contribute to the knowledge of health professionals about the evaluation and prescription of physical exercises for older adults, so that they can continue projects like this in professional practice.

The researchers ensure that the results data will be disclosed individually to the research participants.

8. Critical Risk and Benefit Analysis

8.1 What are the direct or indirect benefits for the population and society?

The direct benefits of this research for the population studied involve participation in a planned and controlled physical exercise program, which can be performed both at home and in groups, and which can promote positive effects on maintaining and increasing physical function, increasing lower limbs muscle strength and power, improvements in balance and walking pattern, consequently reducing the risk of falls and increasing the quality of life of participants. Indirectly, the present study will contribute to the training of professionals and academics involved in the prescription of exercises for older adults and their effects. At the end of the research, the data and results will be disseminated to all participants and collaborators, as well as in congresses and scientific publications, contributing to scientific knowledge in the area.

8.2 What are the inherent or arising risks of the research?

Participants can experience some discomfort in certain assessments and exercises, such as the risk of late-onset muscle pain (up to 48 hours after practice) that is common when participating in a physical exercise program. As the body adapts to exercise these pains should no longer occur. There are also the possibilities of muscle injury during practice, which will be minimized by a warm-up period at the beginning of the session and relaxation at the end of the session. In addition, it is possible that the participant feels constrained when answering the questionnaires, but if this occurs, the participant may interrupt the interview or choose not to answer, without causing any damage to their participation in the project.

8.3 What is the possibility of the occurrence?

Muscle pain may occur when loading adjustment and/or start of a new series of exercises.

8.4 What are the measures for its minimization and protection of the research participant?

The exercise sessions will be preceded by warm-up periods and stretching and relaxation exercises will be applied at the end of each session. In addition, there will be a period of adaptation to the exercise program and the loads will be individualized and will undergo progressive increase throughout the program.

If the participant is embarrassed when answering any of the questionnaires, the participant may interrupt the interview or choose not to answer.

All the actions mentioned above will contribute to reducing the risk of any negative event during the study period, however, if it occurs, appropriate measures will be taken to make sure that the participant is attended to and treated immediately.

9. Duração Total da Pesquisa | Cronograma

The survey starts from the approval of the CEP/SD and ends in December 2021. The recruitment and performance of physical exercise programs will be carried out for two years, according to the schedule below.

MÊS/ANOS	AÇÕES
**Janeiro/2020**	Divulgação do projeto
**Fevereiro/2020**	Mapeamento e recrutamento de participantes
**Março a Abril/2020**	Avaliações iniciais
**Maio a Julho/2020**	Intervenção com programa de exercício
**Agosto a Setembro/2020**	Avaliações finais
**Outubro/2020**	Avaliação de follow-up
**Novembro a Dezembro/2020**	Apresentação de resultados aos participantes
**Janeiro/2021**	Divulgação do projeto
**Fevereiro/2021**	Mapeamento e recrutamento de participantes
**Março a Abril/2021**	Avaliações iniciais
**Maio a Julho/2021**	Intervenção com programa de exercício
**Agosto a Setembro/2021**	Avaliações finais
**Outubro/2021**	Avaliação de follow-up
**Novembro/2021**	Apresentação de resultados aos participantes
**Dezembro/ 2021**	Análise de dados total

10. Criteria to Suspend or End the Search

Not reaching the number of volunteers needed to carry out the study.

11. Location of Research

Pre- and post-intervention evaluations will be carried out at the Center for Motor Behavior Studies (CECOM) at UFPR and exercise programs will be conducted in the rhythmic activities room of the Department of Physical Education (DEF/UFPR). In the case of the group of home exercises, the activities will be carried out at home and will be monitored by telephone contact and home visits performed by the researchers.

11. Statement of Infrastructure Existence

The evaluations and the intervention program will be carried out in the premises of the Department of Physical Education (UFPR). The evaluation instruments and materials for carrying out the exercise program belong to the Center for Motor Behavior Studies (CECOM) of DEF/UFPR.

12. Ownership of Information

The information regarding the data collected during the research will be about the custody and confidentiality of the responsible researcher, Prof. Dr. Paulo Cesar Barauce Bento.

13. Information Relating to the Survey Participant and Characteristics of the Population to be Studied

Men and women aged 60 years or older, living in the community, without distinction in terms of color, race, ethnicity, and social class, will participate in the study.

14. Vulnerable Groups

It doesn't apply.

15. Research Material Sources

All material listed will be used for this research:

FINALIDADE	MATERIAL	OBTENÇÃO
Avaliações	*Notebook*	DEF/UFPR
Avaliações	Dinamômetro isocinético Biodex System (Biodex Medical Systems Inc., Shirley, NY, USA)	DEF/UFPR
Avaliações	Tapete Zeno Walkway e software ProtoKinetics Movement Analysis (PKMAS)	DEF/UFPR
Avaliações	Sistema de análise tridimensional do movimento (VICON)	DEF/UFPR
Avaliações	Plataforma de força (AMTI, modelo OR-06, USA)	DEF/UFPR
Avaliações	Microsoft Kinect	DEF/UFPR
Avaliações	Equipamento de ultrassom (Konica Minolta Medical Imaging Inc Newark-Pompton Turnpike, Wayne, NJ, USA)	DEF/UFPR
Avaliações	Eletromiógrafo Trigno Wireless, Delsys, USA;	DEF/UFPR
Avaliações	Estimulador elétrico modelo Neuro IOM – Neurosoft®	DEF/UFPR
Avaliações	Cadeira	DEF/UFPR
Avaliações	Cone	DEF/UFPR
Avaliações	Dinamômetro de preensão manual	DEF/UFPR
Avaliações	Sala de rítmicas	DEF/UFPR
Avaliações	Caneleiras	DEF/UFPR

16. Plans for Research Participant Recruitment

Participants will be recruited from the dissemination of the project by UFPR media, social media, and local newspapers. Interested parties will be able to contact the

researchers for information about the research and will be invited to participate in an initial meeting where the objectives and procedures will be presented. Those who voluntarily agree to participate will sign the Free and Informed Consent Form.

17. Inclusion and Exclusion Criteria

The study will include men and women aged 60 years or older, who are independent in performing basic daily activities, without cognitive impairment determined through the Mini-Mental State Examination (MMSE) with a score stratified by schooling, who are not participating in any training program or regular and systematized physical exercise for at least 6 months and who do not fit any exclusion criteria.

Older adults with physical or motor limitations that make it impossible to perform functional tests, with a recent history of stroke, heart failure, who use pacemakers, who do not control unstable medical conditions (e.g., diabetes mellitus and hypertension), with neurological disease, with severe osteoporosis with a history of two or more fractures, who do not walk independently and who make use of orthosis. After selecting the participants according to the above criteria, the older adults will be asked for a medical certificate with the release to perform physical activity, and thus, the older adults who present absolute contraindications for participation in the proposed exercise protocol will be excluded.

18. Measures to Protect or Minimize Any Possible Risk

During the tests or participation in the classes, the participant may experience some discomfort related to muscle or joint pain, which are common when practicing physical activity, especially at the beginning, when his body is not yet accustomed to the new activity. However, as the body adapts to exercises these pains should no longer occur. When participating in physical training the participant runs the risk of at some point getting hurt or feeling body pain due to effort. Possible muscle pain or discomfort will be minimized by performing an adequate warm-up period and individual exercise load dosage based on initial assessments. To ensure adaptation to exercise, loads will be gradually increased every 3 weeks of exercise. The subjective perception of effort (Borg scale) will

be used as an auxiliary way to monitor adaptation to the program. If necessary, the medical service of the agreement with Plus Santé will be activated.

19. Forecast of Reimbursement of Costs to Research Participants

The expenses necessary for the conduct of the research are not the responsibility of the participants, including the displacement of the participant, and they will not receive any cash amount.

20. Reference

ABE, T.; KUMAGAI, K.; BRECHUE, W. F. Fascicle length of leg muscles is greater in sprinters than distance runners. **Applied Sciences**, v. 1, n. 14, p. 1125–1129, 2000.

AMERICAN COLLEGE OF SPORTS MEDICE. Exercise and physical activity for older adults. **Medicine and Science in Sports and Exercise**, v. 41, n. 7, p. 1510–1530, 2009.

AMERICAN THORACIC SOCIETY. ATS statement: Guidelines for the six-minute walk test. **American Journal of Respiratory and Critical Care Medicine**, v. 166, n. 1, p. 111–117, 2002.

ARRIETA, H. et al. A multicomponent exercise program improves physical function in long-term nursing home residents: A randomized controlled trial. **Experimental Gerontology**, v. 103, n. October 2017, p. 94–100, 2018.

BAUMAN, A. et al. Updating the Evidence for Physical Activity: Summative Reviews of the Epidemiological Evidence, Prevalence, and Interventions to Promote “active Aging”. **Gerontologist**, v. 56, n. April, p. S268–S280, 2016.

BEAUCHET, O. et al. Stops walking when talking: A predictor of falls in older adults? **European Journal of Neurology**, v. 16, n. 7, p. 786–795, 2009.

BERTOLUCCI, P. H. et al. O Mini-Exame do Estado Mental em uma população geral. **Arquivos de Neuro-Psiquiatria**, v. 52, n. 1, p. 1–7, 1994.

BLAZEVICH, A. J.; SHARP, N. C. C. Understanding muscle architectural adaptation: Macro- and micro-level research. **Cells Tissues Organs**, v. 181, n. 1, p. 1–10, 2005.

BRADY, A. O.; STRAIGHT, C. R.; EVANS, E. M. Body composition, muscle capacity, and physical function in older adults: An integrated conceptual model. **Journal of Aging and**

Physical Activity, v. 22, n. 3, p. 441–452, 2014.

BYRNE, C. et al. Ageing , Muscle Power and Physical Function : A Systematic Review and Implications for Pragmatic Training Interventions. **Sports Medicine**, 2016.

CADORE, E. L. et al. Multicomponent exercises including muscle power training enhance muscle mass, power output, and functional outcomes in institutionalized frail nonagenarians. **Age**, v. 36, n. 2, p. 773–785, 2014.

CICONELLI, R. et al. Tradução para a língua portuguesa e validação do questionário genérico de avaliação de qualidade de vida SF-36 (Brasil SF-36). **Revista Brasileira de Reumatologia**, v. 39, n. 3, p. 8, 1999.

CLARK, B. C.; MANINI, T. M. Functional consequences of sarcopenia and dynapenia in the elderly. **Curr Opin Clin Nutr Metab Care**, v. 13, n. 3, p. 271–276, 2010.

COSTA, S. N.; VIEIRA, E. R.; BENTO, P. C. B. Effects of Home- and Center-Based Exercise Programs on the Strength , Function , and Gait of Prefrail Older Women : A Randomized Control Trial. **Journal of Aging and Physical Activity**, p. 1–10, 2019.

CRESS, M. E. et al. Best Practices for Physical Activity Programs and Behavior Counseling in Older Adult Populations. **Journal of Aging and Physical Activity**, v. 13, n. 1, p. 61–74, 2005.

DALY, R. M. et al. Effectiveness of dual-task functional power training for preventing falls in older people: study protocol for a cluster randomised controlled trial. **Trials**, v. 16, n. 1, p. 1–15, 2015.

FERNANDES, A. DE A.; MARINS, J. C. B. Test of hand grip strength: a methodological analysis and normative data in athletes. **Fisioter Mov**, v. 24, n. 3, p. 567–578, 2011.

FOLSTEIN, M. F.; FOLSTEIN, S. E.; MCHUGH, P. R. “Mini-mental state”. A practical method for grading the cognitive state of patients for the clinician. **Journal of Psychiatric Research**, v. 12, n. 3, p. 189–198, 1975.

FRAGALA, M. S.; KENNY, A. M.; KUCHEL, G. A. Muscle Quality in Aging: a Multi-Dimensional Approach to Muscle Functioning with Applications for Treatment. **Sports Medicine**, v. 45, n. 5, p. 641–658, 2015.

FRIED, L. P. et al. Frailty in older adults: Evidence for a phenotype. **Journals of Gerontology: Medical Sciences**, v. 56, n. 3, p. M146–M156, 2001.

GILL, T. M. et al. A program to prevent functional decline in physically frail, elderly persons who live at home. **The New England journal of medicine**, v. 347, n. 14, p. 1068–74,

2002.

GOMES, G. DE C. et al. Gait performance of the elderly under dual-task conditions: Review of instruments employed and kinematic parameters. **Revista brasileira de g**, v. 1, n. 1, p. 165–182, 2016.

GUEDES, D. P. **Manual Prático para avaliação em Educação Física**. 1. ed. São Paulo: 1, 2006.

GURALNIK; et al. A Short Physical Performance Battery assessing lower extremity function: association with self-reported. **J Gerontol**, v. 49, n. 2, p. 85–94, 1994.

HENDELMAN et al. Validity of accelerometry for the assessment of moderate intensity physical activity in the field. **Medicine & Science in Sports & Exercise**, v. 32, n. 9, p. 9, 2000.

HERMENS, H. J. et al. Development of recommendations for SEMG sensors and sensor placement procedures. **Journal of Electromyography and Kinesiology**, v. 10, n. 1, p. 361–374, 2000.

IBGE. Censo demográfico 2010. **Instituto Brasileiro de Geografia e Estatística**, 2010.

KNIGHT, C. A.; KAMEN, G. Adaptations in muscular activation of the knee extensor muscles with strength training in young and older adults. **Journal of Electromyography and Kinesiology**, v. 11, n. 6, p. 405–412, 2001.

LACROIX, A. et al. Effects of Supervised vs . Unsupervised Training Programs on Balance and Muscle Strength in Older Adults : A Systematic Review and Meta-Analysis. **Sports Medicine**, v. 1, n. 1, p. 21, 2017.

LEBRÃO, M. L.; DUARTE, Y. A. O. **O projeto SABE no município de São Paulo: uma abordagem inicial**. 1. ed. Brasília: 1, 2003.

LEXELL, J. Strength Training and Muscle Hypertrophy in Older Men and Women. **Geriatr Rehabil**, v. 15, n. 3, p. 41–46, 2000.

LIM, J. P. et al. Inter-muscular adipose tissue is associated with adipose tissue inflammation and poorer functional performance in central adiposity. **Archives of Gerontology and Geriatrics**, v. 81, p. 1–7, 2019.

LOK, N.; LOK, S.; CANBAZ, M. The effect of physical activity on depressive symptoms and quality of life among elderly nursing home residents: Randomized controlled trial. **Archives of Gerontology and Geriatrics**, v. 70, p. 92–98, 2017.

LUSTOSA et al. Tradução e adaptação transcultural do Minnesota Leisure Time Activities

Questionnaire em idosos. **Geriatria & Gerontologia**, v. 5, n. 2, p. 57–65, 2011.

MANINI, T. Development of Physical Disability in Older Adults. **Current Aging Sciencee**, v. 4, n. 3, p. 184–191, 2012.

MAYR, S. et al. A short tutorial of GPower. **Tutorials in Quantitative Methods for Psychology**, v. 3, n. 2, p. 51–59, 2007.

MCBEAN, A. L. et al. Standing Balance and Spatiotemporal Aspects of Gait Are Impaired Upon Nocturnal Awakening in Healthy Late Middle-Aged and Older Adults. **Journal of Clinical Sleep Medicine**, v. 12, n. 11, p. 1477–1486, 2016.

MCPHEE, J. S. et al. Physical activity in older age: perspectives for healthy ageing and frailty. **Biogerontology**, v. 17, n. 3, p. 1–14, 2016.

MIRANDA, G.; MENDES, A.; SILVA, A. Population aging in Brazil: corrent and futere social challenges and consequences. **Revista Brasileira de Geriatria e Gerontologia**, v. 19, n. 3, p. 195–198, 2016.

MITCHELL, W. K. et al. Sarcopenia, dynapenia, and the impact of advancing age on human skeletal muscle size and strength; a quantitative review. **Frontiers in Physiology**, v. 3 JUL, n. July, p. 1–18, 2012.

NARICI, M. V.; MAGANARIS, C. N. Adaptability of elderly human muscles and tendons to increased loading. **Journal of Anatomy**, v. 208, n. 4, p. 433–443, 2006.

NELSON, J. K.; THOMAS, J. R. **Métodos de Pesquisa em Atividade Física**. 6. ed. Brasil: 1, 2012.

PETERS, M. Footedness: Asymmetries in Foot Preference and Skill and Neuropsychological Assessment of Foot Movement. **Psychological Bulletin**, v. 103, n. 2, p. 179–192, 1988.

PODSIADLO, D.; RICHARDSON, S. The Timed ”Up & Go”: A Test of Basic Functional Mobilitv for Frail Elderly Persons. **Journal of American Geriatrics Society**, p. 142–148, 1991.

RADLOFF, L. S. The CES-D Scale : A Self-Report Depression Scale for Research in the General Population. **Applied Psychological Measurement**, v. 1, n. 3, p. 385–401, 1977.

REEVES, N. D.; MAGANARIS, Æ. C. N.; NARICI, M. V. Ultrasonographic assessment of human skeletal muscle size. **European Journal of Applied Physiology**, v. 91, p. 116–118, 2004.

RIKLI, R. E.; JONES. **Sênior Fitness Test Manual**. 5 edition ed. São Paulo: Manole,

2011.

ROAD, R. **Biodex Medical Systems**, 2000.

ROZAND, V. et al. Assessment of Neuromuscular Function Using Percutaneous Electrical Nerve Stimulation. **Journal of Visualized Experiments**, n. 103, p. 1–11, 2015.

RUBENSTEIN, L. Z. Falls in older people: Epidemiology, risk factors and strategies for prevention. **Age and Ageing**, v. 35, n. SUPPL.2, p. 37–41, 2006.

SELVA RAJ, I.; BIRD, S. R.; SHIELD, A. J. Ultrasound Measurements of Skeletal Muscle Architecture Are Associated with Strength and Functional Capacity in Older Adults. **Ultrasound in Medicine and Biology**, v. 43, n. 3, p. 586–594, 2017.

SINGH, M. A. F. Exercise Comes of Age Rationale and Recommendations .pdf. **Journal of Gerontology**, v. 57, n. 5, p. 262–282, 2002.

SPIRDUSO. **Dimensões Físicas do Envelhecimento**. 5. ed. Brasil: 1, 2005.

STATHI, A.; MCKENNA, J.; FOX, K. R. Processes associated with participation and adherence to a 12-month exercise programme for adults aged 70 and older. **Journal of Health Psychology**, v. 15, n. 6, p. 838–847, 2010.

TEIXEIRA, J. **Associação entre os componentes da sobrecarga de treinamento e alterações na função, composição e arquiteratura muscula no atleta master de corrida**. [s.l: s.n.].

TIELAND, M.; TROUWBORST, I.; CLARK, B. C. Skeletal muscle performance and ageing. **Journal of Cachexia, Sarcopenia and Muscle**, v. 9, n. 1, p. 3–19, 2018.

UNITED NATIONS. **World Population Ageing**. [s.l: s.n.].

VALLABHAJOSULA, S. et al. Concurrent Validity of the Zeno Walkway for Measuring Spatiotemporal Gait Parameters in Older Adults. **Journal of Geriatric Physical Therapy**, v. 0, n. 0, p. 1, 2017.

VERAS, R. Envelhecimento populacional contemporâneo: demandas, desafi os e inovações - Population aging today: demands, challenges and innovations. **Revista de Saúde Pública**, v. 43, n. 3, p. 548–554, 2009.

WARE, J. E.; GANDEK, B. Overview of the SF-36 Health Survey and the International Quality of Life Assessment (IQOLA) Project. **J Clin Epidemiol**, v. 51, n. 11, p. 903–912, 1998.

WEI, N.; NG, G. Y. F. The effect of whole body vibration training on quadriceps voluntary activation level of people with age-related muscle loss (sarcopenia): A randomized pilot

study. **BMC Geriatrics**, v. 18, n. 1, p. 1–6, 2018.

WU, S.; PARK, K.-S.; MCCORMICK, J. B. Effects of Exercise Training on Fat Loss and Lean Mass Gain in Mexican-American and Korean Premenopausal Women. **International Journal of Endocrinology**, v. 2017, p. 1–7, 2017.

22. Anexos

APPENDIX 1 - ANAMNESE

FICHA DE AVALIAÇÃO

Avaliador: __________________________ CÓDIGO: ________ DATA: ___/____/_____

Data de nascimento: ______________________________________ Idade: ________

Endereço: ____

Contato telefônico: ____

Massa corporal: _____________ kg Estatura: __________ m IMC: _______ kg/m²

Circunf. Abdominal: __________ cm Comprimento da perda: D ______ E ________

NOME: ____

Mão que escreve: ____________ Perna dominante: _________ (teste: subir escada)

**Escolaridade:**<br>( ) Analfabeto<br>( ) 1-4 anos<br>( ) 5-8 anos<br>( ) >8 anos<br>( ) Superior incomp.<br>( ) Superior completo<br>( ) Pós-graduação	**Situação conjugal**<br>( ) Casado<br>( ) Divorciado<br>( ) Separado<br>( ) Viúvo<br>( ) Solteiro	**Profissão:**<br>__________<br>**Ocupação**<br>( ) Aposentado com outra ocupação<br>( ) Aposentado sem outra ocupação<br>( ) Trabalhos domésticos<br>( ) Trabalho fora do domicilio	**Renda**<br>( ) Aposentadoria<br>( ) Pensão<br>( ) Mesada dos filhos<br>( ) Aluguel<br>( ) Trabalho<br>Outras_____________
**Local de residência**<br>( ) Casa térrea<br>( ) Casa duplex<br>( ) Apartamento<br>( ) ILP<br>Outros________	**Residência**<br>( ) Sozinho<br>( ) Filhos<br>( ) Outros familiares<br>( ) Cuidadores<br>Outros_______	**Religião**<br>( ) Católica<br>( ) Evangélica<br>( ) Espírita<br>( ) Budista<br>Outra____________	**Etnia**<br>( ) Negra<br>( ) Branca<br>( ) Parda<br>( ) Amarela<br>Outra_______________
VISÃO<br>( ) Visão normal<br>( ) Déficit visual<br>( ) Usa corretores	AUDIÇÃO<br>( ) normal<br>( ) Déficit auditivo	CIRURGIAS<br>( ) Sim<br>( ) Não<br>Qual?____________	Uso de órtese:<br>____________________<br>Uso de prótese:

	( )Usa corretores		____________________
DOENÇAS<br>( ) Hipertensão<br>( ) Diabetes<br>( ) Osteoporose<br>( ) Dislipidemia<br>( ) Art<br>( ) Artrose<br>( ) Problema na tireoide<br>( ) Visão/Cataratas<br>( ) Deficiência Auditiva<br>( ) Incontinência urinári	Medicamentos:<br>Número de medicamentos:<br>( ) Hormônio: ____________________<br>( ) Diurético: _____________________<br>( ) Antidepressivo: _________________<br>( ) Pressão Arterial: _______________<br>( ) Anti-inflamatórios: ______________<br>( ) Analgésicos: ___________________<br>( ) Cardiovasculares: ______________<br>( ) Vitaminas: _____________________<br>( ) Suplementos: __________________<br>( ) Outros____________________________<br>__________________________________		Frequência:<br>________________<br>________________<br>________________<br>________________<br>________________<br>________________<br>________________<br>________________<br>________________<br>________________
Histórico de cardiopatia na família?<br>( )Sim ( ) Não<br>Quem?<br>_______________	Tabagismo? ( )Sim ( ) Não<br>Frequência: ________________<br>Bebidas alcoólicas? ? ( )Sim ( ) Não<br>Frequência: _______<br>Atividade física atual? ? ( )Sim ( ) Não		Histórico de cardiopatia na família?<br>( )Sim ( ) Não<br>Quem?<br>_______________

Atividade	Tempo de prática	Frequência semanal

APPENDIX 2 – MINI EXAME DO ESTADO MENTAL

Avaliador: ____________________ CÓDIGO: ______________ DATA: ___/____/_____

Orientação Temporal Espacial

1. Qual é o dia da semana? .... (1)
   Dia do mês? .... (1)
   Mês? .... (1)
   Ano? .... (1)
   Hora aproximada? .... (1)
2. Onde estamos?
   Local? .... (1)
   Instituição (casa,rua)? .... (1)
   Bairro? .... (1)
   Cidade? .... (1)
   Estado? .... (1)

Registro

1. Mencione 3 palavras levando 1 segundo para cada uma. Peça ao paciente para repetir as 3 palavras que você mencionou. Estabeleça um ponto para casa resposta correta.
   - Vaso, carro, tijolo
   .... (3)

Linguagem

5. Aponte para um lápis e um relógio. Faça o paciente dizer o nome desses objetos conforme você os aponta
.... (2)

6. Faça o paciente repetir “nem aqui, nem ali, nem lá”.
.... (1)

7. Faça o paciente seguir o comando de 3 estágios “Pegue o papel com a mão direita. Dobre o papel ao meio. Coloque o papel na mesa”.
.... (3)

8. Faça o paciente ler e obedecer ao seguinte: FECHE OS OLHOS
.... (1)

9. Faça o paciente escrever uma frase de sua própria autoria. (A frase deve conter um sujeito e um objeto e fazer sentido). (Ignore erros de ortografia ao marcar o ponto).
.... (1)

10. Copie o desenho abaixo.
Estabeleça um ponto se todos os lados e ângulos forem preservados e se os lados da interseção formarem um quadrilátero

Atenção e cálculo

"Sete" seriado. Dê 1 ponto para cada correto. Interrompa após 5 perguntas. Alternativamente soletre a palavra MUNDO de trás para frente

.... (5)

Lembrança (memória de evocação)

Pergunte o nome das três palavras aprendidas na questão 2. Estabeleça um ponto para cada resposta correta.

.... (3)

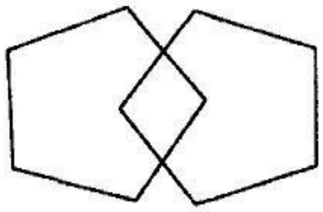

.... (1)

APPENDIX 3 – IDENTIFICAÇÃO DO FENÓTIPO FRAGILIDADE

Avaliador: __________________ CÓDIGO: ______________ DATA: ___/____/_____

Nome: ____

Data de nascimento: ______/______/_______ **Idade**: __________ anos

Estado civil: ( ) solteiro ( ) casado ( ) divorciado ( ) viúvo

Telefone: ___________________________ **Telefone celular:** _______________________

TRIAGEM DO FENÓTIPO FRAGILIDADE

Antropometria

Massa corporal: .... kg **Estatura**: .... m **IMC**: .... kg/m²

Circunf. Abdominal: .... **Compr. Perna**: **D** .... mm **E** .... mm

1. **Perda de peso não intencional**

4,5 Kg ou 5% do peso corporal no último ano ( ) Sim ( ) Não

2. **Preensão Manual – Lado Direito**

.... kgf

Homens: ( ) ≤ 21 kgf

Mulheres:

( ) IMC ≤ 24 FP ≤ 14

( ) IMC 24.1-27 FP ≤ 15

( ) IMC 27.1-31 FP ≤ 17

( ) IMC > 31 FP ≤ 14

.... kgf

.... kgf

3. Exaustão/Fadiga

a) Senti que tive que fazer esforço para dar conta das minhas tarefas habituais?

(Zero) Nunca ou Raramente (2) As vezes (3) Maioria das vezes ou sempre.

b) Não consegui levar a diante minhas coisas?

(Zero) Nunca ou Raramente (2) As vezes (3) Maioria das vezes ou sempre.

4. Velocidade Da Marcha - Teste de 4 metros

( ) sim ( ) não

.... s

.... s

.... s

Homens:

( ) altura ≤ 169 cm ≥ 5 segundos

( ) altura > 169 cm ≥ 6 segundos

Mulheres:

( ) altura ≤ 153 cm ≥ 6 segundos

( ) altura > 153 cm ≥ 5 segundos

5. Baixa Atividade Física – Minnesota Leis

Gasto energético por semana inferior: Homens: a 383 kcal e Mulheres: 270 kcal

QUESTIONÁRIO MINNESOTA DE ATIVIDADES FÍSICAS, ESPORTE E LAZER

**A ser completado pelo participante**	**Você realizou esta atividade?**		**1ª semana**	**2ª semana**	**Tempo por ocasião**
**Atividade**	**não**	**sim**	**(média de x última semana)**	**(média de x penúltima semana)**	**(minutos)**
**Seção A: Caminhada**					
010 Caminhada recreativa					
020 Caminhada para o trabalho					
030 Uso de escadas quando o elevador está disponível					
040 Caminhada ecológica					
050 Caminhada com mochila					
060 Alpinismo/escalando montanhas					
115 Ciclismo recreativo/por prazer					
125 Dança – salão, quadrilha e/ou discoteca, danças regionais					

135 Dança/ginástica – aeróbia, balé					
140 Hipismo/andando a cavalo					
**Seção B: Exercício de condicionamento**					
150 Exercícios domiciliares					
160 Exercício em clube/em academia					
180 Combinação de caminhada/corrida leve					
200 Corrida					
210 Musculação					
**Seção C: Atividades aquáticas**					
220 Esqui aquático					
235 Velejando em competição					
250 Canoagem ou remo recreativo					
260 Canoagem ou remo em competição					
270 Canoagem em viagem de acampamento					
280 Natação em piscina (pelo menos 15 metros)					
295 Natação na praia					
310 Mergulho autônomo					
320 Mergulho livre – snorkel					
**Seção D: Atividades de inverno**					
340 Esquiar na montanha					
350 Esquiar no plano					
360 Patinação no gelo ou sobre rodas					
370 Trenó ou tobogã					
**Seção E: Esportes**					
390 Boliche					
400 Voleibol					
410 Tênis de mesa					
420 Tênis individual					
430 Tênis de duplas					
480 Basquete sem jogo (bola ao cesto)					
490 Jogo de basquete					
500 Basquete como juiz					
520 Handebol					
530 Squash					
540 Futebol					

Golf					
070 Dirigir carro de golfe					
080 Caminhada, tirando os tacos do carro					
090 Caminhada carregando os tacos					
**Seção F: Atividades no jardim e na horta**					
550 Cortar a grama dirigindo um carro de cortar grama					
560 Cortar a grama andando atrás do cortador de grama motorizado					
570 Cortar a grama empurrando o cortador de grama manual					
580 Tirando o mato e cultivando o jardim/horta					
590 Afofar, cavando e cultivando a terra no jardim e na horta					
600 Trabalho com ancinho na grama					
610 Remoção de neve/terra com pá					
**Seção G: Atividades de reparos domésticos**					
620 Carpintaria em oficina					
630 Pintura interna de casa ou colocação de papel de parede					
640 Carpintaria do lado de fora da casa					
650 Pintura exterior de casa					
**Seção H: Pesca**					
660 Pesca na margem do rio					
670 Pesca em correnteza com botas					
**Seção I: Outras atividades (descrever)**					

APPENDIX 4 - TESTES FUNCIONAIS

Avaliador: ____________________ CÓDIGO: ______________ DATA: ___/____/_____

**Testes Funcionais**	
**Sentar e levantar da cadeira (SPPB)**	**TEMPO: ______:_______**
**Equilíbrio (SPPB)**	**Unipodal: ______________seg**<br>**Semi-tandem: ___________seg**<br>**Tandem: _______________seg**
**Levantar e caminhar cronometrado (TUG)**	**1 - TEMPO: ______:_______**<br>**2 - TEMPO: ______:_______**
**Sentar e alcançar**	____________________ cm
**Alcançar atrás das costas**	____________________ cm
**Flexão de antebraço**	_________________ repetições
**Teste de caminhada de 6 minutos**	_________________________ metros

APPENDIX 5 - MEDICAL OUTCOMES STUDY 36 – SF-36

CÓDIGO

SF – 36 PEQUISA EM SAÚDE

Instruções: Esta pesquisa questiona você sobre sua saúde. Estas informações nos manterão informados de como você se sente e quão bem você é capaz de fazer suas atividades de vida diária. Responda cada questão marcando a resposta como indicado. Caso você esteja inseguro em como responder, por favor, tente responder o melhor que puder.

1 - **Em geral você diria que sua saúde é: (Circule uma)**

Excelente 1
Muito Boa 2
Boa 3
Ruim 4
Muito Ruim 5

2 **- Comparada há 1 ano atrás,** como você classificaria sua saúde em geral, agora? **(Circule uma)**

Muito melhor agora do que há um ano atrás 1
Um pouco melhor agora do que há um ano atrás 2
Quase a mesma de um ano atrás 3
Um pouco pior agora do que há um ano atrás 4
Muito pior agora do que há um ano atrás 5

3 **-** Os seguintes itens são sobre atividades que você poderia fazer atualmente durante um dia comum. **Devido a sua saúde**, você tem dificuldade para fazer essas atividades? Neste caso, quanto? **(circule um número em cada linha)**

**Atividades**	**Sim dificulta muito**	**Sim dificulta um pouco**	**Não. Não dificulta de modo algum**
a - **Atividades vigorosas**, que exigem muito esforço: correr, levantar objetos pesados, participar em esportes árduos.	1	2	3
b – **Atividades moderadas**, tais como: mover uma mesa, passar aspirador de pó, jogar bola, varrer a casa.	1	2	3
c **-** Levantar ou carregar mantimentos	1	2	3
d - Subir **vários** lances de escada	1	2	3
e – Subir **um lance** de escada	1	2	3
f – Curvar-se, ajoelhar-se ou dobrar-se	1	2	3
g – Andar **mais de 1 quilômetro**	1	2	3
h – Andar **vários quarteirões**	1	2	3
i – Andar **um** quarteirão	1	2	3
j – Tomar banho ou vestir-se	1	2	3

4 – Durante **as últimas 4 semanas**,você teve algum dos seguintes problemas com o seu trabalho ou com alguma atividade diária regular, como conseqüência de sua saúde física?

	**Sim**	**Não**
a – Você diminuiu a **quantidade de tempo** que se dedicava ao seu trabalho ou a outras atividades?	1	2
b – Realizou **menos tarefas** do que você gostaria?	1	2
c – Esteve **limitado** no seu trabalho ou em outras atividades?	1	2
d – Teve **dificuldade** de fazer seu trabalho ou outras atividades? (necessitou de um esforço extra?)	1	2

5 – Durante as últimas 4 semanas, você teve algum dos seguintes problemas com o seu trabalho ou outra atividade regular diária,como conseqüência de algum problema emocional (como sentir-se deprimido ou ansioso)?

	**Sim**	**Não**
a – Você diminuiu a **quantidade de tempo** que se dedicava ao seu trabalho ou a outras atividades?	1	2
b – Realizou **menos tarefas** do que você gostaria?	1	2
c – Não trabalhou ou não fez qualquer das atividades com tanto **cuidado** como geralmente faz?	1	2

6 – Durante as **últimas 4 semanas**, de que maneira sua saúde física ou problemas emocionais interferiram nas suas atividades sociais normais, em relação a família, vizinhos, amigos ou em grupo? **(Circule uma)**

De forma nenhuma 1
Ligeiramente 2
Moderadamente 3
Bastante 4
Extremamente 5

7 – Quanta dor no corpo você teve durante **as últimas 4 semanas**?

Nenhuma 1
Muito Leve 2
Leve 3
Moderada 4
Grave 5
Muito Grave 6

8 – Durante **as últimas 4 semanas**, quanto a dor interferiu com o seu trabalho normal (incluindo tanto o trabalho, fora de casa e dentro de casa)?

De maneira alguma 1
Um pouco 2

Moderadamente 3
Bastante 4
Extremamente 5

9 – Estas questões são sobre como você se sente e como tudo tem acontecido com você durante as últimas 4 semanas. Para cada questão, por favor dê uma resposta que mais se aproxime da maneira como você se sente. Em relação as últimas 4 semanas.

	**Todo tempo**	**A maior parte do tempo**	**Uma boa parte do tempo**	**Alguma parte do tempo**	**Uma pequena parte do tempo**	**Nunca**
a - Quanto tempo você tem se sentido cheio de vigor, cheio de vontade, cheio de força?	**1**	**2**	**3**	**4**	**5**	**6**
b – Quanto tempo você tem se sentido uma pessoa muito nervosa?	**1**	**2**	**3**	**4**	**5**	**6**
c - Quanto tempo você tem se sentido tão deprimido que nada pode animá-lo?	**1**	**2**	**3**	**4**	**5**	**6**
d - Quanto tempo você tem se sentido calmo ou tranqüilo?	**1**	**2**	**3**	**4**	**5**	**6**
e - Quanto tempo você tem se sentido com muita energia?	**1**	**2**	**3**	**4**	**5**	**6**
f - Quanto tempo você tem se sentido desanimado e abatido?	**1**	**2**	**3**	**4**	**5**	**6**

g- Quanto tempo você tem se sentido esgotado?	**1**	**2**	**3**	**4**	**5**	**6**
h - Quanto tempo você tem se sentido uma pessoa feliz?	**1**	**2**	**3**	**4**	**5**	**6**
I - Quanto tempo você tem se sentido cansado?	**1**	**2**	**3**	**4**	**5**	**6**

10 – Durante as **últimas 4 semanas**, quanto do seu tempo a **sua saúde física ou problemas emocionais** interferiram com as suas atividades sociais (como visitar amigos, parentes, etc.)?

Todo o tempo 1
A maior parte do tempo 2
Alguma parte do tempo 3
Uma pequena parte do tempo 4
Nenhuma parte do tempo 5

11 – O quanto verdadeiro ou **falso** é cada uma das afirmações

	**Definitivamente verdadeiro**	**A maioria das vezes verdadeira**	**Não sei**	**A maioria das vezes falsa**	**Definitivamente falsa**
a – Eu costumo adoecer um pouco mais facilmente que as outras pessoas	1	2	3	4	5
b – Eu sou tão saudável quanto qualquer pessoa que eu conheço.	1	2	3	4	5
c – Eu acho que a minha saúde vai piorar	1	2	3	4	5
d – Minha saúde é excelente	1	2	3	4	5
