# Supplementary material for: The effects of supervision on three different exercises modalities (supervised vs. home vs. supervised+home) in older adults: Randomized controlled trial protocol
Source: PLoS One. 2021 Nov 15;16(11):e0259827. doi: 10.1371/journal.pone.0259827 (PMC8592418; doi:10.1371/journal.pone.0259827)
Supplement: S2 File — (PDF) [file pone.0259827.s002.pdf]

Projeto de Pesquisa

DESCRIÇÃO DA PESQUISA

**Título do Projeto**	Comparação de um programa de exercício multicomponente nas modalidades presencial e/ou domiciliar na função neuromuscular, composição e arquitetura muscular de idosos da comunidade
**Pesquisador Principal**	Paulo Cesar Barauce Bento
**Colaboradores**	Jaciara do Carmo Frasão<br>Maria Luiza Tavares da Silva<br>Sabrine Nayara Costa
**Local de Realização**	Universidade Federal do Paraná - Departamento de Educação Física (DEF-UFPR)
**Período de Pesquisa**	01/2020 – 12/2021

1. Objetivo da Pesquisa

1.1 Objetivo Geral

O objetivo deste estudo é comparar os efeitos de um programa de exercício físico multicomponente em diferentes modalidades de aplicação (presencial x domiciliar x misto) e verificar as adaptações neuromusculares em idosos da comunidade.

1.2 Objetivos Específicos

- Comparar os efeitos de um programa de exercícios com sessões em domicílio e em grupo (misto) aos efeitos de um programa presencial e domiciliar sobre a função musculoesquelética (função neuromuscular, composição e arquitetura muscular) de idosos da comunidade.
- Comparar os efeitos de um programa de exercícios com sessões em domicílio e em grupo (misto) aos efeitos de um programa presencial e domiciliar sobre os parâmetros espaço temporais da marcha e custo da dupla tarefa de idosos da comunidade.
- Comparar os efeitos de um programa de exercícios com sessões em domicílio e em grupo (misto) aos efeitos de um programa presencial e domiciliar sobre o desempenho de um conjunto de testes funcionais e qualidade de vida de idosos da comunidade.
- Comparar a velocidade de execução do movimento e intensidade do treinamento nas sessões presenciais e domiciliares nas três modalidades do programa de exercício multicomponente.
- Verificar os efeitos de um programa de exercícios com sessões em domicílio e em grupo aos efeitos de um programa estritamente presencial e domiciliar após oito semanas de destreinamento em idosos da comunidade.

2. Relevância Social

O envelhecimento é um processo natural que pode ser acompanhado de alterações física, fisiológicas e psicológicas. Em conjunto estas alterações podem resultar no comprometimento da funcionalidade e qualidade de vida do idoso. Nesse sentido, a

prática regular de exercícios físicos tem sido uma estratégia de contraposição aos efeitos do envelhecimento, podendo prevenir ou retardar o desenvolvimento das doenças crônicas e reduzir a velocidade em que os declínios físicos e fisiológicos ocorrem. Estudos têm mostrado que programas de exercícios físicos supervisionados e realizados em grupo podem amenizar o declínio físico e funcional, prevenir a fragilidade e sua evolução, evitando desta forma consequências como a perda da independência do idoso. No entanto, programas presenciais estão associados a dificuldades em relação à adesão do participante, devido à falta de acesso a esses tipos de programas próximos a residência, dificuldades de locomoção e dependência de familiares ou terceiros para o deslocamento do idoso aos locais de treinamento físico. Alternativamente, a literatura apoia programas de exercícios domiciliares, os quais reduzem essas barreiras e, uma vez que os pacientes estejam comprometidos com eles, têm benefícios a longo prazo. Em contrapartida, nessa modalidade de exercício, a supervisão é fornecida indiretamente por telefonemas ou visitas domiciliares, que podem resultar em limitações quanto à qualidade de execução do exercício, como a realização dos exercícios com menor amplitude de movimento, intensidade menos vigorosa e com pausas mais longas, que resulta em menor intensidade do exercício quando comparado ao treinamento presencial. Dessa forma, uma possibilidade de modelo de programa de exercício físico para a população idosa é a combinação de um programa de exercícios com sessões semanais presenciais e domiciliares. A sessão presencial proporcionaria aumento do convívio social dos idosos e maior supervisão do exercício, visto que a principal limitação do exercício domiciliar é a falta de controle da frequência, intensidade e progressão do exercício.

3. Hipóteses a Serem Testadas

Um programa de doze semanas com sessões de exercícios em domicílio e em grupo (misto) melhorará a função neuromuscular, marcha, funcionalidade e qualidade de vida de idosos da comunidade de maneira similar à um programa de exercício físico presencial e terá efeito superior ao programa domiciliar. Dessa forma, as hipóteses gerais serão testadas.

H0 – Não haverá diferenças nas respostas ao treinamento físico nas diferentes modalidades de aplicação do programa de exercício multicomponente.

H1 – O programa com sessões de exercícios em domicílio e em grupo (misto) apresentará aumento da função musculoesquelética (função neuromuscular, composição e arquitetura muscular) similar aos observados no programa de exercício físico presencial e superior ao grupo domiciliar.

H2 – O programa com sessões de exercícios em domicílio e em grupo (misto) apresentará melhora dos parâmetros espaço temporais da marcha e custo da dupla tarefa similar aos observados no programa de exercício físico estritamente presencial e superior ao grupo estritamente domiciliar.

H3 – O programa com sessões de exercícios em domicílio e em grupo (misto) apresentará melhora no desempenho de um conjunto de testes funcionais e qualidade de vida similar aos observados no programa de exercício físico estritamente presencial e superior ao grupo estritamente domiciliar.

H4 – A velocidade de execução do movimento e a intensidade do treinamento será maior nas sessões do grupo presencial e do grupo misto quando comprado ao grupo estritamente domiciliar.

H5 - Os efeitos de um programa de exercícios com sessões em domicílio e em grupo perdurará de maneira similar aos observados no programa de exercício físico presencial e será superior ao grupo domiciliar.

4. Antecedentes Científicos

O envelhecimento populacional é um fenômeno mundial e estima-se que nas próximas décadas o número de pessoas com mais de 60 anos deve chegar a 2 bilhões (UNITED NATIONS, 2013). O aumento da população idosa decorre de mudanças de alguns indicadores de saúde, como aumento da expectativa de vida devido a melhoria nas

condições de saúde, queda dos níveis de fecundidade e mortalidade e aumento do desenvolvimento socioeconômico (VERAS, 2009). No Brasil, o número de idosos cresceu em 40,3% entre os anos de 2002 e 2012, chegando a 20,5 milhões de idosos, aproximadamente 39 idosos para cada grupo de 100 jovens (IBGE, 2010). Projeções estimam que para 2040 a população idosa brasileira será de 23,8%, uma proporção de quase 153 idosos para cada 100 jovens (MIRANDA; MENDES; SILVA, 2016).

O envelhecimento é um processo caracterizado por alterações morfológicas, fisiológicas, bioquímicas e psicológicas que levam a uma diminuição da capacidade de adaptação do indivíduo ao meio ambiente (SPIRDUSO, 2005). Essas alterações levam à declínios na função física, aumento do risco de incapacidade física e perda de independência funcional (BRADY; STRAIGHT; EVANS, 2014), que em conjunto, levam o idosos a desenvolver maior risco de hospitalização, institucionalização, quedas e morte (TIELAND; TROUWBORST; CLARK, 2018).

Alguns fatores podem ser apontados como responsáveis pela diminuição da função física do idosos. Com o envelhecimento, há mudanças na quantidade e qualidade muscular, traduzido pelo aumento do tecido não contrátil e diminuição da massa muscular esquelética (LIM et al., 2019; MITCHELL et al., 2012). A diminuição da quantidade de massa muscular, conhecida como sarcopenia, é consequência da diminuição do número de fibras musculares e redução de tamanho dos sarcômeros (LEXELL, 2000), podendo ser acompanhada pela perda de força muscular, conhecida como dinapenia (CLARK; MANINI, 2010).

Além das modificações morfológicas, alterações do sistema neuromuscular também contribuem para a diminuição do desempenho físico de idosos (TIELAND; TROUWBORST; CLARK, 2018), afetando a capacidade contrátil, elevando a co-ativação da musculatura antagonista e reduzindo o recrutamento e o sincronismo de ativação das unidades motoras (CLARK; MANINI, 2010; NARICI; MAGANARIS, 2006). Em conjunto, esses fatores influenciam a contração e condução elétrica (FRAGALA; KENNY; KUCHEL, 2015) e podem causar redução da mobilidade, dificuldades em realizar atividades da vida diária como subir e descer escadas, sentar e levantar de uma cadeira e, consequentemente, elevar o risco de quedas acidentais (MANINI, 2012).

Nesse sentido, a prática regular de exercícios físicos é uma estratégia de contraposição aos efeitos do envelhecimento. Ela pode prevenir ou retardar o

desenvolvimento das doenças crônicas e reduzir a velocidade em que as alterações físicas e fisiológicas ocorrem (AMERICAN COLLEGE OF SPORTS MEDICE, 2009; MCPHEE et al., 2016). Além disso, o exercício físico oferece uma das maiores oportunidades para prolongar os anos de vida ativa independente, reduzir incapacidades e melhorar a qualidade de vida de idosos (BAUMAN et al., 2016; CRESS et al., 2005).

Programas de exercício físico têm se mostrado efetivos no aumento da massa muscular, diminuição da infiltração de gordura intramuscular (WU; PARK; MCCORMICK, 2017), aumento da condução elétrica do músculo, aumento da força e potência muscular (CADORE et al., 2014; RUBENSTEIN, 2006), velocidade da marcha (RUBENSTEIN, 2006), funcionalidade física (ARRIETA et al., 2018; CADORE et al., 2014) e qualidade de vida (LOK; LOK; CANBAZ, 2017). Esses programas têm sido aplicados tanto na modalidade presencial (em grupo) quanto na modalidade domiciliar (individual).

Quando comparado as duas modalidades de aplicação, programas de exercício presenciais em grupo parecem ser mais efetivos na função muscular, qualidade de vida e funcionalidade que programas domiciliares (LACROIX et al., 2017). Além disso, programas de exercícios presenciais possivelmente oferecem maiores desafios cognitivos, devido à necessidade do planejamento do transporte, o deslocamento até o local de prática (demanda física e cognitiva) e a interação social com os demais participantes, estímulos que ficam ausentes quando realizado o exercício em domicílio (STATHI; MCKENNA; FOX, 2010). No entanto, programas presenciais estão associados a dificuldades em relação à adesão do participante, devido à falta de acesso a esses tipos de programas próximos a residência, dificuldades de locomoção e dependência de familiares ou terceiros para o deslocamento do idoso aos locais de treinamento físico (LACROIX et al., 2017; STATHI; MCKENNA; FOX, 2010).

Alternativamente, a literatura apoia programas de exercícios domiciliares, os quais reduzem essas barreiras e, uma vez que os pacientes estejam comprometidos com eles, têm benefícios a longo prazo (GILL et al., 2002). Além disso, o treinamento em domicílio é capaz de ajudar o indivíduo a aprimorar o treinamento independente, podendo sustentá-lo por períodos mais longos e facilitar a participação a programas de exercício, pois não necessitam sair de suas casas e conseguem adequar o treinamento à sua rotina (BYRNE et al., 2016; LACROIX et al., 2017). Em contrapartida, nessa modalidade de exercício, a supervisão é fornecida indiretamente por telefonemas ou visitas domiciliares, que podem

resultar em limitações quanto à qualidade de execução do exercício, como a realização dos exercícios com menor amplitude de movimento, intensidade menos vigorosa e com pausas mais longas, que resulta em menor intensidade do exercício quando comparado ao treinamento presencial (LACROIX et al., 2017).

Nesse sentido, uma recente meta análise sobre exercício a efetividade de programas de exercícios domiciliares verificaram que a maior efetividade de programas presenciais poderia ser anulada ou diminuída com a adição de maior supervisão à programas domiciliares (LACROIX et al., 2017). Dessa forma, um programa de exercício físico que alie sessões presenciais e em domicílio é uma alternativa válida, pois a sessão presencial proporciona o aumento do convívio social dos idosos e maior supervisão do exercício, visto que a principal limitação do exercício domiciliar é a falta de controle da frequência, intensidade e progressão do exercício (COSTA; VIEIRA; BENTO, 2019, *IN PRESS*).

Estudo anterior desenvolvido em nosso laboratório testou esse modelo de programa de exercício físico combinado de sessões presenciais e domiciliares em idosas pré-frágeis e constatou que os ganhos de força muscular de membros inferiores foram similares entre aqueles que realizam programa de treinamento estritamente presencial (COSTA; VIEIRA; BENTO, 2019, *IN PRESS*). No entanto, não foi verificado quais mecanismos foram responsáveis pela melhora da função muscular após o programa de treinamento. Sabe-se que os mecanismos responsáveis pelo aumento da força muscular podem ser atribuídos a uma combinação de fatores neurais e morfológicos, como o aumento da quantidade de massa muscular, diminuição da infiltração de gordura, aumento do número de unidades motoras, melhora da taxa de disparo e diminuição da co-ativação da musculatura antagonista (TIELAND; TROUWBORST; CLARK, 2018). Até onde sabemos, nenhum estudo verificou se existem diferenças nos mecanismos neuromusculares e fisiológicos responsáveis pelo aumento da força muscular e funcionalidade em idosos em diferentes modalidades de exercícios físico (domiciliar e/ou presencial). Conhecer a efetiva contribuição dos mecanismos morfológicos e neurais sobre a força muscular e funcionalidade após programas de exercícios aplicados de formas diversas (domicílio/presencial) é essencial para o planejamento de programas efetivos e viáveis para a população idosa, além de ampliar as estratégias terapêuticas ao idoso que apresenta dificuldade em comparecer ao local de treinamento.

Dessa forma, identifica-se a necessidade de verificar a contribuição do sistema neuromuscular nas adaptações ao exercício físico em suas diferentes formas de aplicação: estritamente domiciliar, estritamente presencial e misto (sessões presenciais e domiciliares) e as contribuições dessas adaptações para a melhora da funcionalidade de idosos. Sendo assim, objetivo deste estudo é comparar os efeitos de um programa de exercício físico multicomponente com diferentes formas de aplicação (presencial x domiciliar x misto) e verificar a contribuição do sistema neuromuscular nas adaptações ao exercício físico em suas diferentes formas de aplicação. A hipótese do estudo é que a adição de uma sessão presencial em um programa de treinamento domiciliar acarretará melhoras na função, qualidade e composição muscular, na marcha, na funcionalidade e na qualidade de vida de idosos de maneira similar à um programa de exercício físico estritamente presencial e terá efeito superior ao programa estritamente domiciliar.

5. Casuística

O envelhecimento populacional é um fenômeno generalizado e sem precedentes em quase todos os países, onde estima-se que nas próximas décadas o número de pessoas com mais de 60 anos deve chegar a 2 bilhões (UNITED NATIONS, 2013). Atualmente, os idosos correspondem a aproximadamente 30,2 milhões de pessoas, e representam uma parcela significativa da população brasileira. Nos últimos cinco anos a população idosa no país aumentou 4,8 milhões, o que corresponde a um crescimento de 18%. Nesse grupo, as mulheres são maioria expressiva com 16,9 milhões (56% dos idosos), enquanto os homens idosos são 13,3 milhões (44% do grupo).

No estado do Paraná, a população idosa aumentou 15,92% nos últimos cinco anos, totalizando 1.717.889 milhões de pessoas (INSTITUTO BRASILEIRO DE GEOGRAFIA E ESTATÍSTICA, 2017). Em 2017, Curitiba registrou 268,7 mil pessoas com idade igual a superior a sessenta anos. Na escala de projeção do envelhecimento, o número de idosos na cidade de Curitiba deve subir para 307,3 mil em 2020; 432,5 mil em 2030 e 544,5 mil em 2040.

6. Material e Metodologia

6.1 CARACTERÍSTICAS DO ESTUDO

Trata-se de um estudo do tipo experimental, randomizado, simples cego. Na pesquisa experimental, o pesquisador determina um objeto de estudo, seleciona as variáveis capazes de influenciar esse objeto e define formas de controle, observando os efeitos que a variável produz sobre o objeto de estudo (NELSON; THOMAS, 2012). Este estudo será aprovado pelo Comitê de ética em Pesquisa do Setor de Ciências da Saúde da Universidade Federal do Paraná.

Para o cálculo amostral foi utilizada a calculadora GPower (MAYR et al., 2007). Foi realizada uma análise a priori com os seguintes parâmetros de entrada: tamanho do efeito (0,25) (COSTA; VIEIRA; BENTO, 2019, *IN PRESS*), erro do tipo I (0,05), erro tipo II (0,80), número de grupos (3), número de medições (3) e correlação entre grupo (0,5). Em adição, foi considerada uma taxa de possíveis perdas de 15%. Dessa forma, nossa análise revelou um tamanho amostral de 42 participantes, que serão divididos em 3 grupos de 14 participantes.

6.2 PROCEDIMENTOS

Os idosos interessados em participar do projeto receberão informações detalhadas sobre os objetivos e procedimentos da pesquisa em reunião agendada por telefone. Os idosos que aceitarem participar, comparecerão ao Centro de Estudos do Comportamento Motor (CECOM) na Universidade Federal do Paraná para entrevista. Após esta etapa, os idosos que atenderem aos critérios de inclusão assinarão o Termo de Consentimento Livre e Esclarecido, de acordo com os critérios do Comitê de Ética do Setor de Ciências da Saúde da Universidade Federal do Paraná atendendo à resolução 466/2012 do CNS e complementares. Logo após os participantes responderão um questionário referente às características sócio demográficas, classificação econômica, estado cognitivo, risco de queda, e realizarão uma bateria de avaliação física.

Após essa etapa, os idosos serão randomizados em três grupos experimentais: grupo de exercício presencial (GP, n=14), grupo de exercício domiciliar (GD, n=14) e grupo de

exercício com sessão presencial e domiciliar (GPD, n=14). Os participantes dos grupos experimentais serão submetidos a 12 semanas de treinamento com exercício físico e após finalizado o período de 12 semanas, os grupos serão reavaliados e passarão por um acompanhamento de 8 semanas. Nesse período de acompanhamento os participantes serão orientados a manter suas atividades habituais. O período de acompanhamento será nomeado como período follow-up. Ao final desse período, os participantes serão reavaliados. Detalhes sobre o modelo experimental são apresentados na Figura 1.

FIGURA 1 - FLUXOGRAMA DO MODELO EXPERIMENTAL PROPOSTO

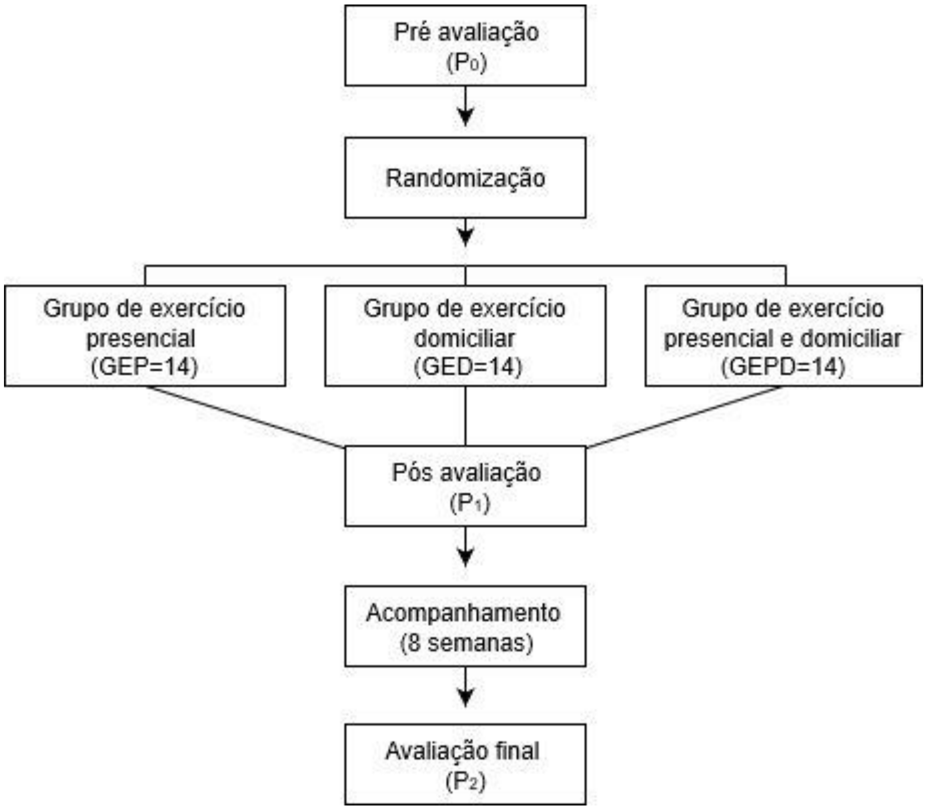

FONTE: O autor (2019).

6.3 PROTOCOLOS DE AVALIAÇÃO

As sessões de avaliação serão desenvolvidas no laboratório de biomecânica do Centro de Estudos do Comportamento Motor (CECOM), no Departamento de Educação Física da Universidade Federal do Paraná (UFPR). Todas as avaliações e protocolo de treinamento serão aplicadas por uma equipe de avaliadores previamente treinados e com

experiência nos protocolos utilizados. Os avaliadores não participarão da aplicação do programa de exercício físico e os aplicadores do programa de exercício físico não participarão da avaliação, caracterizando um estudo simples cego.

Na pré-avaliação (P_0) os participantes serão submetidos a uma bateria de testes que serão aplicados em dois dias, respeitando um intervalo mínimo de 48 horas entre as avaliações. No primeiro dia, os participantes responderão a uma anamnese clínica, seguida de avaliação antropométrica, avaliação do fenótipo da fragilidade, testes funcionais de força muscular, flexibilidade e velocidade da marcha e uma avaliação dos parâmetros espaço temporais da marcha com e sem dupla tarefa. Nessa primeira avaliação também será entregue um acelerômetro com orientações para a utilização. No segundo dia, o participante será submetido a avalição do equilíbrio dinâmico, teste de força de flexão e extensão do quadril, joelho e tornozelo na cadeira isocinética e avaliação da ativação elétrica muscular e avaliação da arquitetura muscular pelo ultrassom.

As variáveis primárias do estudo serão a função neuromuscular, composta pela avaliação da força muscular, arquitetura e ativação elétrica do músculo. As variáveis secundárias serão equilíbrio dinâmico, funcionalidade, avaliação dos parâmetros espaço-temporais da marcha usual e com dupla tarefa e qualidade de vida.

Todos os protocolos aplicados serão são descritos com detalhes a seguir.

6.3.1 Caracterização da amostra

A caracterização dos voluntários será realizada por meio de anamnese clínica, avaliação antropométrica, estado mental e fragilidade.

A anamnese será composta por questões objetivas sobre escolaridade, situação conjugal, profissão, renda, moradia, etnia, condições de saúde (visão e audição), histórico de cirurgias, uso de medicamentos, hábitos de vida (tabagismo, consumo de bebida alcoólica e histórico de atividade física), doenças associadas e histórico de quedas (ANEXO 1). Para a avaliação do histórico de quedas, o voluntário será questionado sobre a ocorrência de quedas no último ano por meio da pergunta: “O/a Sr(a). apresentou algum episódio de queda nos últimos doze meses?”. Se a resposta foi positiva, aspectos relacionados ao local da queda, a frequência e suas consequências serão questionadas.

A avaliação antropométrica será composta por avaliação da estatura, massa corporal, circunferência abdominal e índice de massa corporal (IMC).

Para avaliação da estatura será utilizado um estadiômetro fixo à parede com escala de 1 mm. O voluntário será orientado a ficar descalça e posicionada anatomicamente, com a cabeça e o tronco posicionados o mais ereto possível, cabeça orientada paralela ao solo e com o peso corporal distribuído igualmente em ambos os pés. Após o posicionamento adequado, o ponto mais alto da cabeça será utilizado como referência (GUEDES, 2006).

A massa corporal será mensurada por meio de pesagem em uma balança digital. A voluntária será posicionada em cima da balança em posição anatômica, descalça e com roupas o mais leve possível, com o rosto direcionado para frente e peso distribuído em ambos os pés (GUEDES, 2006).

A medição da circunferência abdominal será realizada com uma fita antropométrica com precisão de 0,1 cm e com o voluntário em posição ortostática. A fita circundará o indivíduo no ponto médio entre a última costela e a crista ilíaca (GUEDES, 2006). Será realizada três medições e o valor médio será utilizado.

Para obtenção do IMC será realizado o seguinte cálculo: IMC = massa corporal(kg)/ estatura(m)², para verificar se estes apresentam peso adequado, sobrepeso ou obesidade. Para a classificação do IMC será utilizado os pontos de corte recomendados pela Organização Pan-americana de Saúde (Quadro 1), no projeto Saúde, Bem-estar e Envelhecimento (SABE) que pesquisou o perfil dos idosos nos países da América Latina (LEBRÃO; DUARTE, 2003).

QUADRO 1 - CLASSIFICAÇÃO DO ÍNDICE DE MASSA CORPORAL PARA IDOSOS

**ÍNDICE DE MASSA CORPORAL**	**STATUS DO PESO**
> 23,0	Abaixo do peso
23,0 – 27,9	Peso normal
28,0 – 30,0	Pré-obesidade
≥ 30,0	Obesidade

FONTE: ORGANIZAÇÃO PAN-AMERICANA DE SAÚDE (2003).

O estado cognitivo mental dos idosos será avaliado por meio do Mini Exame do Estado Mental (MEEM) (FOLSTEIN; FOLSTEIN; MCHUGH, 1975) (ANEXO 2). O MEEM é um instrumento dividido em duas seções. A primeira requer apenas respostas vocais e

abrange orientação, memória e atenção com pontuação máxima de 21 pontos. A segunda parte avalia a capacidade de nomear, seguir comandos verbais e escritos, escrever uma sentença espontaneamente e copiar um polígono complexo, sendo a pontuação máxima 9 pontos. A pontuação total máxima é de 30 pontos e seu ponto de corte é definido pelo nível de escolaridade, sendo 20 pontos para analfabetos, 25 pontos para 1-4 anos de escolaridade, 26,5 pontos para 5- 8 anos de escolaridade, 28 pontos para 9-11 anos de escolaridade e 29 pontos para indivíduos com escolaridade superior a 11 anos (BERTOLUCCI et al., 1994). Os idosos que apresentarem pontuação abaixo do ponto de corte definido pela escolaridade serão excluídos do estudo, sendo considerados com comprometimento cognitivo.

Para avaliação do fenótipo fragilidade serão utilizados os critérios propostos por Fried et al (2001), sendo estes a perda de peso, fraqueza, exaustão, diminuição da velocidade da marcha e nível de atividade física (ANEXO 3). Idosos que se enquadrem em um ou dois dos critérios supracitados serão considerados pré-frágeis e aqueles que se enquadrem em três ou mais, serão considerados frágeis. Detalhes sobre os critérios a seguir.

O critério perda de peso é pontuado quando o idoso auto relatar perda de peso não intencional de 4,5 kg ou ≥ 5% do peso no ano anterior. O idoso será questionado por meio da pergunta: “No último ano, você perdeu mais de 4,5 kg involuntariamente (ou seja, não devido à dieta ou exercício) ” (FRIED et al., 2001).

O critério fraqueza será pontuado aquele que apresentar força de preensão manual inferior a 20% da linha base, ajustado pelo Índice de Massa Corporal e estratificado por sexo, sendo ponto de corte apresentado do Quadro 2. A força de membros superiores será avaliada por meio do dinamômetro de preensão manual SH com as mesmas especificações do JAMAR, com escala de medida variando de 0 a 100 quilogramas força (kgf). O teste será realizado com o idoso em posição sentada, com o ombro levemente aduzido, cotovelo posicionado a 90° de flexão e antebraço e punho em posição neutra. Após o posicionamento, serão realizados três movimentos máximos de preensão, com um minuto de intervalo de recuperação, sendo utilizada a média das três tentativas (FERNANDES; MARINS, 2011).

QUADRO 2 – PONTO DE CORTE FORÇA DE PREENSÃO MANUAL

Força de preensão, estratificada por sexo e índice de massa corporal (IMC)			
**MULHERES**	**Ponto de corte**	**HOMENS**	**Ponto de corte**
IMC ≤ 23	Força de preensão ≤ 17	IMC ≤ 24	Força de preensão ≤ 29
IMC 23.1-26	Força de preensão ≤ 17.3	IMC 24.1-26	Força de preensão ≤ 30
IMC 26.1-29	Força de preensão ≤ 18	IMC 26.1-29	Força de preensão ≤ 30
IMC > 29	Força de preensão ≤ 21	IMC > 29	Força de preensão ≤ 32

FONTE: Adaptado de (FRIED et al., 2001).

O critério exaustão será avaliado por meio de duas questões retiradas da escala de depressão do Center for Epidemiological Stidies (RADLOFF, 1977). Os voluntários serão questionados sobre a exaustão no último mês com as perguntas: “O/a senhor/a sentiu que teve que fazer esforço para fazer tarefas habituais? ” e “O/a senhor/a consegue levar as suas coisas adiante?”. As seguintes opções de respostas serão dadas: (a) raramente ou nunca, (b) as vezes (1-2 dias) ou, (c) a maior parte do tempo ou sempre (3 a 4 dias). Será pontuado no critério o voluntário que responder as alternativas b e c (FRIED et al., 2001).

A diminuição da velocidade da marcha será verificada por meio do tempo gasto em segundos para percorrer uma distância de 4 metros. O voluntário percorrerá, em linha reta, um percurso de 8 metros, havendo cones demarcando o ponto inicial e final. Será cronometrado o tempo percorrido nos quatro metros intermediários. O voluntário será orientado a caminhar de um cone ao outro na sua velocidade usual, “como se estivesse caminhando normalmente na rua” (GURALNIK; et al., 1994). Para a pontuação no critério, será adotado o ponto de corte ajustado pelo sexo e altura. Mulheres: altura ≤ 159 cm ≥ 7 segundos e altura > 159 cm ≥ 6 segundos e homens altura ≤ 173 cm ≥ 7 segundos e altura > 173 cm ≥ 6 segundos (FRIED et al., 2001).

O nível de atividade física será verificado por meio do questionário Minnesota Leisute Time Activity (TAYLOR et al., 1978). O Questionário Minnesota Leisute Time Activity é um instrumento que avalia o nível de atividade física, esporte e lazer de acordo com o gasto energético, sendo adaptado e validado para a população brasileira (LUSTOSA et al., 2011). O gasto energético semanal será calculado obtendo a média do gasto energético das duas últimas semanas. Será pontuado no critério aquele que teve o gasto energético por semana inferior a 383 kcal para homens e 270 para mulheres.

6.4.2 Avaliação da Função Neuromuscular

A avaliação da função neuromuscular será composta pela avaliação da função muscular, atividade elétrica e qualidade muscular.

6.4.2.1 Avaliação da Função Muscular

A avaliação da função muscular será realizada por meio da utilização do dinamômetro Biodex Multi-joint System (Biodex Medical Systems. Inc.. Shirley. NY. USA). Serão adquiridas 3 repetições máximas dos torques concêntricos dos grupos musculares extensores e flexores de joelho e quadril e dorsi e plantiflexores do tornozelo do membro dominante às velocidades de 60 e 180°/s. A avaliação será sempre iniciada pela articulação do joelho, seguida pelo tornozelo e quadril, pela maior facilidade de ajuste do equipamento. O membro dominante será definido perguntando aos participantes: “se você fosse chutar uma bola, com qual pé chutaria?” (PETERS, 1988).

Cada teste será realizado duas vezes, na mesma sessão de avaliação, diferenciadas pelo comando verbal do avaliador: primeiramente com séries submáximas para a familiarização e após, com séries máximas para registro.

Para familiarização, o avaliador explicará o teste e demonstrará o movimento a ser executado de maneira passiva e na sequência, será solicitado movimentos de flexão e extensão ativo. O protocolo para registro será composto por três repetições máximas dos torques concêntricos dos grupos musculares dos extensores e flexores de joelho e quadril e plantiflexores e dorsiflexores do tornozelo do membro dominante às velocidades de 60 e 180°/s, com um intervalo de 1 minuto entre os testes. Esses grupos musculares foram escolhidos por sua ativação em atividades funcionais habituais, como caminhar, subir e descer escadas e sentar e levantar da cadeira (BYRNE et al., 2016). As velocidades de 60 e 180°/s foram escolhidas por analisarem a força e a potência dos membros inferiores, respectivamente. Durante o teste os avaliadores incentivarão verbalmente os idosos a realizar o máximo de força na maior velocidade possível, com as frases “mais rápido” e “mais forte”.

Os participantes serão posicionados de acordo com as especificações de fábrica do Biodex, que são descritas por Symons et al (2004) e serão apresentadas a seguir.

Para a avaliação do joelho, a voluntária será posicionada sentada confortavelmente na cadeira do equipamento, com o encosto inclinado a 85º e estabilizada por cintos no tronco, cruzando o quadril, em volta de uma das coxas e em volta do tornozelo do membro a ser testado logo acima do maléolo medial. A cadeira será posicionada de maneira que o epicôndilo lateral do joelho avaliado fique alinhado com o eixo de rotação do braço do equipamento (ROAD, 2000). Serão anotadas as medidas de altura da cadeira, inclinação do encosto, altura do dinamômetro, rotação da cadeira e do dinamômetro, posicionamento da cadeira e do dinamômetro e comprimento do braço de resistência. Essas medidas serão anotadas para padronizar a posição de teste de cada participante, individualmente e para garantir o mesmo posicionamento na reavaliação.

Para a avaliação do quadril, a voluntária será posicionada deitada confortavelmente na cadeira do equipamento, com o encosto inclinado a 0º e estabilizada por cintos no tronco, cruzando o quadril e em volta da coxa do membro a ser testado logo acima do joelho. A cadeira será posicionada de maneira que o trocânter do fêmur do membro avaliado estivesse alinhado com o eixo de rotação do braço do equipamento(ROAD, 2000).

Para a avaliação do tornozelo, os participantes serão posicionados com o joelho em flexão de 30° e com o encosto do banco inclinado em um ângulo de 40 °.Um braço será fixado sob a coxa para estabilização. A estabilização do membro será realizada por uma faixa na cintura e na coxa. O pé do participante será posicionado de modo que o eixo de rotação do tornozelo esteja alinhado com o eixo de rotação do dinamômetro. O pé será fixado em uma plataforma duas alças, uma distal ao tornozelo e a outra sobre os ossos metatarsais.

A aquisição do sinal é realizada a frequência de 1000 Hz e todos os testes serão analisados usando o Biodex System 3 Advantage software, version 3.2. Serão analisadas as variáveis:

- Pico de Torque (PT), obtido pelo maior valor de torque de extensão e flexão de uma determinada curva, expresso em N.m;
- Pico de Torque normalizado pela massa corporal (PT/MC), obtido pela divisão do pico de torque pela massa corporal, expresso em % da massa corporal;
- Potência média (POT), que representa a velocidade em que os músculos extensores e flexores do joelho são capazes de realizar trabalho, expressa em Watts;

- Trabalho total (TT), que consiste no cálculo da área das curvas de extensão e flexão, expresso em Joules.
- Máxima repetição do trabalho total (MRTT), que representa a repetição com a maior quantidade de trabalho, expresso em foot pounts.

6.4.2.2 Função neuromuscular e nível de ativação voluntária

Para avaliar a função neuromuscular será utilizada a técnica não invasiva de eletromiografia de superfície (EMGs), a qual utiliza eletrodos colocados sobre a pele para registro dos estímulos elétricos musculares. A eletromiografia é excelente método não invasivo para a avaliação da função neuromuscular, pois consegue verificar alterações a nível muscular, espinhal e supraespinhal (HERMENS et al., 2000). Além disso, esse método é amplamente utilizado para verificar efeito de treinamento físico em idosos (KNIGHT; KAMEN, 2001; WEI; NG, 2018)

Para determinar o nível de ativação voluntária (VAL), será utilizado a técnica da twitch interpolation technique (superimposição de um estímulo elétrico - ITT). Nela, o torque extra induzido estimulando o nervo é comparado a uma contração de controle produzida pela estimulação nervosa idêntica com o músculo relaxado. Dessa maneira, o VAL fornece um índice da capacidade do sistema nervoso central de ativar ao máximo os músculos ao trabalho. O efeito induzido por estimulação elétrica reflete um recrutamento incompleto de unidades motoras e/ou uma frequência de disparo submáximo das unidades motoras e, portanto, um déficit na ativação voluntária (ROZAND et al., 2015)

A estimulação elétrica consiste em induzir um estímulo elétrico em um nervo motor para evocar uma resposta muscular. A resposta mecânica (torque) e a resposta eletrofisiológica (atividade eletromiográfica) são gravadas simultaneamente. O protocolo do presente estudo irá estimular o nervo tibial posterior, que ativa os músculos flexores plantares (tríceps sural - sóleo e gastrocnêmio) (ROZAND et al., 2015). A escolha desse protocolo se justifica pelo fato desses músculos estarem envolvidos na locomoção (BYRNE et al., 2016).

Serão analisadas a resposta eletrofisiológica direta - onda M, e a resposta indireta - reflexo-H. A resposta mecânica - torque de contração, será quantificada pelo dinamômetro isocinético Biodex Multi-joint System. A onda M e o torque de contração

refletem a transmissão neuromuscular e a relação entre excitação-contração muscular, enquanto o reflexo H fornece um índice de excitabilidade em nível espinhal. A atividade EMG e as respostas mecânicas (contração superimposta) serão registradas durante contrações voluntárias máximas, a fim de avaliar o nível de ativação voluntária (VAL). O VAL fornece um índice da capacidade do sistema nervoso central de ativar ao máximo os músculos em atividade.

Para a aquisição dos sinais de EMG será utilizado o eletromiógrafo Trigno Wireless, Delsys, USA; usando um estimulador elétrico modelo Neuro IOM – Neurosoft® com eletrodos de superfície AMBU Neuroline 715. O músculo analisado será o tríceps sural do membro dominante, por ser um músculo amplamente solicitado nas atividades diárias. O membro dominante será definido perguntando aos participantes: “se você fosse chutar uma bola, com qual pé chutaria?” (PETERS, 1988). Serão utilizados eletrodos bipolares com superfícies metálicas responsáveis por fazer a aquisição dos dados eletromiográficos. Antes da colocação do eletrodo, a pele será raspada, levemente esfregada com gel abrasivo e limpa com algodão embebido no álcool, como forma de evitar possíveis interferências no sinal eletromiográfico (HERMENS et al., 2000). Após a limpeza, os eletrodos serão fixados à pele por meio de fita adesiva dupla-face descartável, para minimizar possíveis deslocamentos dos eletrodos. Para o músculo sóleo, o eletrodo será posicionado a 2/3 da linha entre o côndilo medial do fêmur e o côndilo medial do maléolo; para o gastrocnêmio medial, na protuberância mais proeminente do músculo; para o gastrocnêmio lateral, a 1/3 da distância ao longo de uma linha entre a cabeça da fíbula e o calcanhar; e para o tibial anterior, a 1/3 da distância ao longo de uma linha entre a ponta da fíbula e a ponta do maléolo medial, no sentido das fibras musculares conforme as recomendações propostas pelo SENIAM (Surface EMG for the Non-Invasive Assessment of Muscles). Também será fixado um eletrodo de referência em posição central na mesma perna (entre os locais de estimulação e gravação).

Como a resposta mecânica induzida pela estimulação do nervo tibial é gerada pelos flexores plantares e eventualmente reduzida pela ativação concomitante do músculo tibial anterior (TA), a atividade eletromiográfica do músculo antagonista e a palpação do músculo/tendão permitirão a exclusão da ativação do TA.

Além disso, será posicionado um elétrodo para estimulação elétrica sobre o tendão patelar. O melhor local para a estimulação do nervo tibial posterior, será definido após

uma série de testes até que um valor máximo seja atingido, onde o cátodo será posicionado na fossa poplítea. Será registrado a atividade EMG do tibial anterior para garantir que o nervo fibular não seja ativado, a fim de evitar a influência de antagonistas. A largura do pulso será de 1 mseg para fornecer uma ativação ideal das fibras nervosas, especialmente as fibras aferentes.

Após a colocação dos eletrodos, o participante será posicionado no dinamômetro isocinético Biodex System, de acordo com a padronização sugerida pelo fabricante para a avaliação do tornozelo, as quais já foram descritas anteriormente (para mais informações verificar item 4.4.1.1). O pé do participante será preso firmemente e irá exercer pressão sobre uma plataforma anexada ao ergômetro para registrar o torque do flexor plantar.

A taxa de amostragem para medições de torque e EMG será definida para 2-5 kHz. O sinal EMG será gravado usando um sistema de conversão analógico-digital (AD). O sinal será exibido em um monitor com um sistema de aquisição de dados, que instantaneamente fornecerá valores de vários parâmetros (valor máximo, amplitude pico a pico, duração). Os sinais EMG serão diferencialmente amplificados com um ganho de 2000 e uma largura de banda de 10-500 Hz a -3 dB. A atividade EMG real será registrada com uma frequência de amostragem de 2000 Hz. Uma janela de 500 mseg será usada para calcular os valores do quadrado médio da raiz EMG (RMS), em torno do tempo da flexão isométrica máximo do tornozelo.

A coleta será realizada durante contração isométrica voluntária máxima. O protocolo da contração isométrica voluntária máxima (CIVM), será composto por três séries com cinco segundos de duração cada. O intervalo de repouso entre cada série será de 120 segundos. Os participantes serão instruídos a realizar a contração com “a maior quantidade de força e o mais rápido possível”. Se for observado um contra movimento inicial (identificado pela queda visual no sinal do torque), a tentativa será descartada e uma nova tentativa será realizada. Durante todas as séries de CIVM, os participantes poderão visualizar suas curvas de torque no monitor do dinamômetro como feedback visual, da mesma forma que serão motivados verbalmente para tentar obter o seu melhor desempenho (DOTAN; MITCHELL, 2013). O maior valor de força atingido nos três esforços máximos será tomado como a CIVM. A variabilidade de 10% entre as medidas será determinada e o maior valor será utilizado para análise.

Para verificar o nível de ativação voluntária, dois doublets supramáximos (dois estímulos elétricos com 200 μs largura de pulso, intervalo inter-estímulo de 10 ms e saída máxima de 1000 mA) serão aplicados pericutaneamente no nervo femoral durante uma contração isométrica voluntária máxima (CIVM), usando um estimulador elétrico modelo Neuro IOM – Neurosoft® com eletrodos de superfície AMBU Neuroline 715.

O estímulo supramáximo será administrado a 350-500 milésimos de segundo após o início do platô da CIVM (contração de sobreposição/superimposição) e novamente 3-5 segundos após, em condição de repouso (contração potenciada). A intensidade do estímulo será determinada pela administração de estímulos elétricos de corrente progressivamente crescente. Os estímulos serão realizados com os participantes em estado de repouso, a partir de 50 mA, aumentando 5 mA até atingir o máximo twitch torque. Então, um adicional de 30% será utilizado para garantir a estimulação supra-máxima.

As variáveis analisadas serão o pico do twitch torque (PT); tempo de contração (TC); taxa de desenvolvimento de torque (RTD = PT/CT); tempo de meio relaxamento e o VAL. O % VAL será calculado usando a equação: [1 – (superimposed doublet torque / rest doublet torque)] x 100.

As tentativas de CIVM serão rejeitadas e repetidas quando a linha do torque não exibir um platô claro antes da estimulação superimposta e quando o estímulo for liberado antes da CIVM.

6.4.2.3 Avaliação da Arquitetura Muscular

A arquitetura muscular dos músculos vasto lateral (VL) e gastrocnêmio medial (MG) serão avaliadas por meio de equipamento de ultrassom (Konica Minolta Medical Imaging Inc Newark-Pompton Turnpike, Wayne, NJ, USA) no modo B com transdutor arranjo linear (4 cm altura x 2 cm comprimento, 10 MHz), com profundidade de coleta ajustada para 5 cm para o VL e 4 cm para o MG. Será avaliado o membro dominante e o transdutor será revestido com gel de transmissão a base de água com quantidade suficiente para garantir que sejam obtidas imagens claras do músculo, sem a necessidade de comprimir os músculos durante a coleta.

Os participantes serão instruídos a não realizar qualquer tipo de exercício físico de membros inferiores nas 48 horas anteriores ao procedimento de aquisição de imagem. Antes da coleta, as participantes deverão repousar por 20 min em decúbito dorsal, com o membro avaliado estendido e relaxado, para permitir acomodação dos fluidos corporais (BERG; TEDNER; TESCH, 1993). Durante as medidas, as participantes serão instruídas a relaxar o membro o máximo possível.

Para avaliar o músculo vasto lateral, as imagens serão obtidas a 39% do comprimento da perna, definido a partir da distância de entre a patela e a crista ilíaca (Blazevich et al. 2007). A inserção proximal do músculo VL será identificada e marcada, sendo as seções axiais marcadas em intervalos de 30. O transdutor será orientado no plano axial, alinhado perpendicularmente ao músculo VL e movido do centro para posição lateral ao longo de um gabarito demarcado sobre a pele.

Para avaliar o músculo gastrocnêmio medial, a participante estará deitada no decúbito ventral em uma maca, com os pés pendurados na borda e será fixado um ângulo da articulação do tornozelo a 15º dorsiflexão. O transdutor do ultrassom será posicionado na porção de 30% entre o maléolo lateral da fíbula e o côndilo lateral da tíbia. Essas especificações foram escolhidas para a avaliação, pois trabalhos anteriores relevaram curvatura mínima do fascículo neste local quando os participantes estavam deitados nessa posição (SELVA RAJ; BIRD; SHIELD, 2017). O transdutor será orientado no plano axial, alinhado perpendicularmente ao músculo e movido do centro para posição lateral ao longo de um gabarito demarcado sobre a pele.

As imagens serão reconstruídas utilizando o PowerPoint (Microsoft, Redmond, WA, EUA), seguindo os procedimentos descritor por Reeves et al (2004) (REEVES; MAGANARIS; NARICI, 2004). Cada imagem será posicionada manualmente até que a fáscia do músculo seja reconstruída. A área de secção transversa do VL será medida usando a planimetria computadorizada, onde será contornada com auxílio do mouse.

As imagens serão analisadas usando o software ImageJ (versão 1.46). O software será calibrado a partir de uma distância conhecida de 1cm nas imagens realizadas pela ferramenta de medida do equipamento de ultrassonografia. Será levado em consideração a intensidade do eco, o comprimento do fascículo, ângulo de penação e espessura muscular. Essas variáveis foram escolhidas pois o comprimento do fascículo muscular desempenha um papel na geração de força durante as contrações, enquanto o ângulo de

penação do fascículo e a espessura muscular são fatores importantes para a geração de força global (BLAZEVICH; SHARP, 2005).

A espessura do músculo vasto lateral será definida como a distância entre a aponeurose superficial e o fêmur. A espessura de músculo gastrocnêmio medial será definida como a distância entre as aponeuroses superficiais e profundas. O ângulo de penação será medido como o ângulo entre os fascículos do músculo e aponeurose profunda. O comprimento do fascículo será medido como comprimento de um fascículo entre suas inserções na aponeurose superficial e profunda. Nos casos em que os fascículos tiverem comprimento para além da imagem gravada, o comprimento do fascículo, a espessura e o ângulo de penação serão estimados utilizando a seguinte equação (ABE; KUMAGAI; BRECHUE, 2000):

$$\text{Comprimento do fascículo} = \text{espessura do músculo} \sin \theta^{-1}$$

onde θ é o ângulo de penação do fascículo muscular determinado pelo ultrassom.

A intensidade do eco foi determinada por meio da análise da escala de cinza, usando a função de histograma padrão no software Imagem-J (National Institute of Health, USA, versão 1.37). Os pixels dentro da área de interesse serão processados com a transformada rápida de Fourier, resultando em uma distribuição de 256 tons de cinza, sendo 0 = preto e 255 = branco. Pixels mais claros (hiperecóicos) podem indicar presença de gordura infiltrada e elementos não contráteis (YOUNG, HUI; JENKINS, NATHAN T.; ZHAO QUN, 2015). A intensidade do eco será calculada como a média e mediana dos valores dentro área de secção transversa, sendo que quanto maior a média e mediana, maior a presença de gordura infiltrada e elementos não contráteis no músculo.

A reprodutibilidade das medidas (média e mediana) será determinada calculando os valores do coeficiente de variação (CV), coeficiente de correlação intraclasse (CCI) e erro típico da medida (ETM) entre as imagens coletadas em 2 dias diferentes, com pelo menos 48 horas de intervalo entre elas. Conforme sugerido por Hopkins (2000), o ETM é o resultante da razão entre o desvio-padrão da diferença das medidas repetidas em dois dias consecutivos (dia 1 e dia 2) e a raiz quadrada de dois (HOPKINS, 2000).

Sabe-se que o CV ideal deve ser o menor possível, preferencialmente igual ou abaixo de 10%, os valores de CCI superiores a 0,9 representam uma alta confiabilidade e entre

0,7 a 0,8, razoável, o ETM utiliza a unidade de medida da variável analisada e o seu valor mínimo (mais próximo de zero) representa uma pequena variabilidade entre as medidas (ATKINSON; NEVILL, 1998).

6.4.3 Avaliação da Marcha

A marcha será avaliada por meio do tapete ProtoKinetics Zeno walkway (ProtoKinetics LLC, Havertown, Pennsylvania), que consiste em uma passarela instrumentada com 16 níveis de sensores de pressão distribuídos em 6,09 m de comprimento e 0,61 metros de largura. A passarela possui três camadas: base de proteção, sensores de pressão e capa de proteção, o qual detecta e coleta dados de pressão em avaliações de marcha. A passarela instrumentada é conectada a um laptop por um cabo de interface serial para processar e armazenar dados usando o ProtoKinetics Movement Analysis Software. A passarela Zeno tem sido usada para investigar características espaço-temporais da marcha em idosos (DALY et al., 2015; MCBEAN et al., 2016) e apresenta excelente validade concorrente (VALLABHAJOSULA et al., 2017).

O protocolo de avaliação será composto por quatro condições distintas: marcha na velocidade habitual, velocidade máxima, velocidade habitual com dupla tarefa e velocidade máxima com dupla tarefa. Os voluntários percorrerão uma distância de 10 metros, onde os dois primeiros e últimos serão considerados tempo de aceleração e desaceleração. Para a velocidade habitual, os participantes serão instruídos a caminhar na velocidade que caminham na rua, por meio do comando: “O/a Sr/a irá realizar o percurso na velocidade que você caminha normalmente na rua”. Para a velocidade máxima, os participantes serão instruídos a caminhar na maior velocidade possível, por meio do comando: “O/a Sr/a. irá caminhar na maior velocidade possível, como se você estivesse perdendo o ônibus”. Além disso, será considerado marcha em velocidade máxima aquela que apresentar, no mínimo, 10% de diminuição do tempo em relação a marcha habitual.

A marcha em dupla tarefa será avaliada por meio de tarefa cognitiva aritmética (contagem regressiva a partir de 50). Essa tarefa foi escolhida por ser a mais utilizada em estudos com idosos e pelo fato que uma tarefa aritmética depende essencialmente da

memória operacional ou de trabalho, um sistema de armazenamento temporário e de processamento de informações, o qual está diretamente relacionado a funções executivas (GOMES et al., 2016). Para a avaliação, os participantes serão orientados a caminhar e realizar a contagem regressiva em voz alta a partir do número 50. Será enfatizado a importância de caminhar e contar ao mesmo tempo, com o melhor da sua capacidade, sem priorizar qualquer tarefa. Os possíveis erros de contagem não serão corrigidos (BEAUCHET et al., 2009).

Serão analisados os seguintes parâmetros espaço-temporais:

- Velocidade da marcha: velocidade do participante no sentido de deslocamento (m/s);

- Cadência: número de passos por unidade de tempo (passos/min);

- Tempo da passada: período de tempo de duração de uma passada (s);

- Comprimento da passada: distância entre o contato inicial do pé analisado e o segundo contato do mesmo pé, projetada no eixo de deslocamento do participante (m);

- Largura da passada: distância lateral entre os calcanhares dos contatos consecutivos dos dois pés (m);

- Tempo de apoio simples: período de tempo o qual somente um pé está em contato com o solo (s);

- Tempo de apoio duplo: período de tempo em que os dois pés estão em contato com o solo (s);

- Tempo de balanço: período de tempo em que o pé está no ar para o avanço do membro (s).

6.4.4 Avaliação do equilíbrio dinâmico

A avaliação do equilíbrio dinâmico será realizada por meio da realização de um jogo de vídeo game, com análise do deslocamento do centro de massa do participante. Para isso, serão utilizadas duas plataformas de força, o sistema de análise tridimensional do movimento (VICON) e Microsoft Kinect®.

O sistema de análise do movimento da empresa Vicon® permite a captura dos movimentos em três planos (x, y, z) por meio de onze câmeras de radiação infravermelha, modelo Vicon® Bonita B10. As câmeras possuem resolução de um megapixel e precisão de até 0.5 mm, em um volume de 4m x 4m. Estas câmeras registram apenas os

marcadores esféricos refletivos posicionados sobre pontos anatômicos específicos de acordo com o modelo biomecânico utilizado. Será utilizado o modelo biomecânico composto por 29 marcadores refletivos, que permite a reconstrução de 9 segmentos corporais, que são necessários para o posterior cálculo do centro de massa (CM).

As plataformas de força que serão utilizadas são da marca AMTI® modelo OR6-7-1000, e serão afixadas no chão com 5 mm de distância entre si e niveladas em altura com um tablado de madeira. As plataformas serão centralizadas dentro da área de captura das câmeras do sistema Vicon® e uma tela de projeção da imagem do equipamento da Microsoft Kinect ® será posicionado a 2 m de distância.

Antes do início da coleta, serão colocados 29 marcadores refletivos de 14 mm de diâmetro posicionados bilateralmente sobre os seguintes pontos anatômicos: face anterior da cabeça, face póstero-superior da cabeça, acrômio, epicôndilo lateral do úmero, processo estiloide da ulna, falange distal do dedo médio, incisura jugular, espinha ilíaca ântero-superior, sacro, maior circunferência femoral, epicôndilo lateral do fêmur, maior circunferência tibial, maléolo lateral, calcâneo e cabeça do 2º metatarso.

Após a colocação dos marcadores, os voluntários serão conduzidos até a área de captura das câmeras do sistema Vicon® e posicionados sobre as plataformas de força.

O jogo utilizado para a coleta é chamado pelo acrônimo SIRMeC, que significa Sistema Interativo de Reabilitação Motora e Cognitiva. Este sistema foi desenvolvido em conjunto com engenheiros, fisioterapeutas e um aluno do curso de Tecnologia em Desenvolvimento de Jogos Digitais da PUCPR, com base nos exercícios fisioterapêuticos de reabilitação neurológica, frequentemente, utilizados na prática clínica (LASKOS, 2014). O Serious Game SIRMeC foi projetado para que diferentes parâmetros de jogo fossem personalizados de acordo com as características particulares de cada indivíduo. Com a finalidade de definir os limites dos desafios lançados na tela, o sistema realiza a calibração da área efetiva de jogo, que possibilita registrar as medidas de amplitude máxima dos movimentos envolvidos em cada jogo, previamente. Sendo este registro feito através do equipamento da Microsoft Kinect® (Microsoft, Redmond, EUA).

O jogo realizado na coleta será o Esqui. O jogo consiste em um ambiente desportivo de inverno, em que o voluntário deve passar por dentro de bandeiras contidas na pista, controlando a direção de seu esqui através da realização dos movimentos de rotação de tronco. Os desafios presentes no jogo são aleatórios e em ambos os lados da pista,

favorecendo o treino de rotação e dissociação de tronco. O jogo possui diferentes níveis de velocidade, sendo moderada, rápida e muito rápida. O voluntário permanecerá em cada nível de jogo por 60 segundos, totalizando 180 s por jogo.

Após processamento e análise dos dados, serão utilizadas as seguintes variáveis: área da base de suporte, margem de estabilidade, distância entre o centro de massa e o as fronteiras da base de apoio e simetria de descarga de peso.

6.4.5 Funcionalidade

A funcionalidade dos participantes será avaliada por meio da bateria de testes do Short Physical Performance Baterry Test (SPPB) e pela bateria de testes de Rick e Jones (ANEXO 4), com o objetivo de verificar a força muscular, agilidade e equilíbrio dinâmico e estático.

A bateria de testes Short Physical Performance Battery (SPPB) é composta por teste de equilíbrio, caminhada em 4 metros e pelo teste de levantar e sentar da cadeira cinco vezes.Para o teste de equilíbrio, o participante deve conseguir manter-se em cada uma das três posições por 10 segundos: em pé com os pés juntos, em pé com os pés em posição semi-tandem e em pé em posição tandem. Nas duas primeiras posições o participante recebe nota 1 caso consiga manter-se na posição por 10 segundos, e nota 0 caso não consiga manter-se por 10 segundos. Na terceira posição, o indivíduo recebe nota 2 caso consiga manter a posição 10 segundos; nota 1 se mantiver a posição por 3 a 9,99 segundos e, nota 0 para o tempo menor que 3 segundos ou caso não realize o teste (GURALNIK; et al., 1994).

No teste de velocidade da marcha, o participante deve caminhar em passo habitual uma distância de 4 metros, demarcados por fitas fixas ao chão. Nota 0 é atribuída ao participante que não conseguir completar o teste, nota 1 se o tempo for maior do que 8,7 segundos, nota 2 o tempo for de 6,21 a 8,7 segundos, nota 3 se o tempo for de 4,82 a 6,2 segundos e nota 4 se o tempo for menor do que 4,82 segundos (GURALNIK; et al., 1994).

Para o teste de levantar e sentar da cadeira, é solicitado ao paciente que o mesmo inicie o teste a posição sentada, com os braços cruzados sobre o tronco e, ao sinal do avaliador, deverá levantar e sentar na cadeira o mais rápido possível, cinco vezes. Se o participante não conseguir levantar-se as 5 vezes ou completar o teste em tempo maior

que 60 segundos é atribuído 0 ponto; se o tempo do teste for de 16,7 seg ou mais, é atribuído 1 ponto; se o tempo do teste for de 13,7 a 16,69 seg, são atribuídos 2 pontos; se o tempo do teste for de 11,2 a 13, 69 seg, são atribuídos 3 pontos; e por fim, tempo do teste menor do que 11,19 seg: 4 pontos (GURALNIK; et al., 1994).

A pontuação final da SPPB é dada pela soma dos três testes, com pontuação máxima de 12 pontos. Será dada a seguinte classificação de acordo com a pontuação: 0 a 3 pontos: incapacidade ou capacidade ruim; 4 a 6 pontos: baixa capacidade; 7 a 9 pontos: capacidade moderada e 10 a 12 pontos: boa capacidade (GURALNIK; et al., 1994).

A bateria de testes Senior Fitness Test (SFT) de Rikli e Jones (2001) é composto pelos testes de levantar e sentar da cadeira, teste de flexão de antebraço, teste de sentar e alcançar, teste Timed-up-and-Go, teste de alcançar atrás das costas e caminhada de 6 minutos.

O teste de flexão de antebraço avalia a força e resistência do membro superior. O participante inicia o teste na posição sentada e segura um halter (2kg para mulheres e 4 kg para homens) com o braço estendido perto da cadeira e perpendicular ao chão. Ao sinal, o participante gira sua palma para cima enquanto flexiona o braço em amplitude total de movimento e então retorna o braço para uma posição completamente estendida. Na posição inicial, o peso deve retornar para a posição de empunhadura de aperto de mão. O avaliado é encorajado a executar tantas repetições quanto possível em 30 segundos. O número total de flexões corretas realizadas no intervalo de 30 segundos será anotado (RIKLI; JONES, 2011).

O teste sentar e alcançar avalia a flexibilidade dos membros inferiores. O participante será orientado a encostar na cadeira somente o glúteo, manter uma perna flexionada, com o pé no chão, os joelhos paralelos, voltados para frente. A outra perda deverá ser estendida à frente do quadril, com o calcanhar no chão e dorsiflexão plantar a aproximadamente 90º. Com a perna estendida, o participante deverá inclinar-se lentamente para a frente mantendo a coluna o mais ereta possível e a cabeça alinhada à coluna. O participante tentará tocar os dedos dos pés escorregando as mãos, uma em cima da outra, com as pontas dos dedos médios, na perna estendida. Para a pontuação, será registrado a distância (cm) até os dedos dos pés ou a distância que se consegue alcançar para além dos dedos dos pés (RIKLI; JONES, 2011).

O teste Timed Up-and-Go (TUG) avalia a mobilidade funcional e equilíbrio dinâmico. O protocolo consiste no voluntário levantar-se de uma cadeira (aproximadamente 46 cm de altura), caminhar até uma linha no chão a 2,44 metros de distância, virar, voltar pelo mesmo percurso e sentar na cadeira novamente, em um ritmo confortável e seguro. O avaliado deve iniciar o teste com o tronco apoiado no encosto da cadeira e ao final, encostar novamente, sendo o tempo cronometrado a partir do comando verbal “já” até o momento que o avaliado apoie novamente o tronco na cadeira. Será realizada uma familiarização e após será registrado o tempo em segundos gasto para completar o teste a partir do comando verbal “já” e finalizado quando o voluntário apoie novamente o tronco na cadeira (PODSIADLO; RICHARDSON, 1991).

O teste alcançar atrás das costas avalia a flexibilidade dos membros superiores. Em pé, o participante coloca a mão de preferência sobre o mesmo ombro, com a palma aberta e os dedos estendidos, alcança o meio das costas tanto quanto possível. A mão do outro braço será colocada atrás das costas e irá tentar tocar ou sobrepor os dedos médios estendidos de ambas as mãos. Para a pontuação, será registrado a distância da sobreposição, ou a distância entre as pontas dos dedos médios. Os resultados negativos (-) representam a distância mais curta entre os dedos médios; os resultados positivos (+) representam a medida da sobreposição dos dedos médios (RIKLI; JONES, 2011).

Por fim, será realizado o teste de caminhada de 6 minutos que avalia a capacidade cardiorrespiratória do indivíduo. O participante será instruído a caminhar, por 6 minutos, a maior distância possível, em terreno plano, em velocidade habitual. O teste de caminhada de 6 minutos utiliza um percurso de 45,7 m medido dentro de segmentos de 4,57 m. Ao final dos 6 minutos o avaliador mede a distância total percorrida (AMERICAN THORACIC SOCIETY, 2002; RIKLI; JONES, 2011).

6.4.6 Qualidade de vida

Para avaliar a qualidade de vida dos participantes será utilizado o questionário Medical Outcomes Study 36 – Item Short-Form Health Survey (SF-36) (ANEXO 5). O SF-36 é um instrumento genérico de avaliação da qualidade de vida, de fácil administração e compreensão. Foi desenvolvido por Ware e Sherbourne (1992) e traduzido e validado para a língua portuguesa por Ciconelli (1999), podendo ser auto aplicado, aplicado via

computorizarão ou aplicado por um treinador previamente treinado. O instrumento é multidimensional formado por 36 itens, englobados em 8 componentes: capacidade funcional, aspectos físicos, dor, estado geral da saúde, vitalidade, aspectos sociais, aspectos emocionais, saúde mental e uma questão comparativa sobre a percepção atual da saúde. Cada componente recebe um escore, que varia de 0 a 100, sendo 0 pior percepção de qualidade de vida e 100 melhor percepção de qualidade de vida (CICONELLI et al., 1999; WARE; GANDEK, 1998).

6.4.7 Avaliação do nível de atividade física

Para avaliar o nível de atividade física inicial e durante o programa de exercícios será utilizado o acelerômetro da marca *Actigraph,* MODELO GT3X. O acelerômetro será utilizado a fim de certificar a realização do treinamento em domicílio, dada as limitações em controlar a realização das sessões em domicílio somente pelo calendário do guia de exercícios. Além disso, será utilizado para comparar a porcentagem de tempo despendido em atividades sedentária, leve, moderada, vigorosa e muito vigorosa nas sessões estritamente presenciais e estritamente domiciliares.

Os participantes serão instruídos a usar o aparelho durante 7 dias consecutivos, o dia inteiro, retirando apenas para dormir e para a realização de atividades aquáticas, incluindo o banho. O aparelho será fixado a uma cinta elástica e posicionado no tornozelo da perna dominante, logo acima do maléolo. Serão considerados como dados válidos o uso do acelerômetro por pelo menos 4 dias, sendo um de final de semana. O dia será considerado válido quando forem registradas no mínimo 10 horas de gravação. Os dados serão coletados numa frequência de 60 Hz. O processo de *download* e análise de dados registrados será realizado pelo *software* Actilife (HENDELMAN et al., 2000).

No software, os valores de *counts* são traduzidos para minutos de atividade física. A intensidade da prática de atividade física será analisada a partir da classificação estabelecida pelo *software* que utiliza a equação de Freedson, sendo: atividade sedentária (0 – 99 contagens/min), leve (100 – 1951 contagens/min), moderada (1952 – 5724 contagens/min), vigorosa (5725 – 9498 contagens/min) e muito vigorosa (>9499 contagens/min) (HENDELMAN et al., 2000).

Serão analisadas as variáveis: média de calorias por dia, tempo em atividades sedentárias, atividades leves, atividades moderadas, atividades vigorosas e muito vigorosas.

6.5 PROGRAMA DE EXERCÍCIOS

Os voluntários participarão de um programa de exercício multicomponente, combinando atividades em grupo presencial e em domicílio durante um período de 12 semanas, com três sessões semanais e duração de 60 minutos, totalizando 180 minutos semanais. O grupo presencial (GEP) realizará todas as sessões de treinamento no DEF/UFPR, o grupo domiciliar (GED) realizará todas as sessões de treinamento em seu domicílio e o grupo do programa com sessões presenciais e domiciliares (GEPD) realizará uma sessão semanal no DEF/UFPR e duas sessões semanais em domicílio.

O programa de exercício será composto por exercícios funcionais de força muscular, equilíbrio, flexibilidade e marcha, baseado nas recomendações de Singh (2002) e ACSM (2009), conforme exposto na Quadro 3. Cada sessão será dividida em 05 minutos de aquecimento dinâmico e articular, com exercícios de grande amplitude, como abdução, adução e rotação dos membros superiores, rotação lateral do tronco, abdução e adução dos membros inferiores, flexão e extensão do quadril; seguida de 10 minutos de exercício específicos de equilíbrio dinâmico e estático, com manipulação da informação visual (olhos abertos e fechados), redução da base de suporte, marcha estacionária e em deslocamento; seguida de 25 minutos de exercícios de fortalecimento muscular de membros inferiores; 10 minutos para exercícios específicos de marcha; e 10 minutos de atividades de flexibilidade, com relaxamento e alongamento dos grandes grupos musculares dos membros superiores, inferiores e do tronco.

QUADRO 3 – PLANEJAMENTO DO PROGRAMA DE EXERCÍCIO

Modalidade	Treinamento de equilíbrio	Treinamento de força	Treinamento de marcha	Treinamento de flexibilidade
**Volume**	1–2 séries<br>4 a 5 exercícios enfatizando posturas estáticas e dinâmicas	3 séries<br>8-12 repetições<br>5 a 6 exercícios envolvendo os maiores grupos musculares	1 série<br>5 repetições<br>6 exercícios envolvendo marcha e dupla tarefa	Principais grupos musculares<br>Sustentar 20s cada
**Intensidade**	Dificuldade progressiva com aumento da complexibilidade, redução da informação visual e manipulação de objetos	15–17 na Escala de Borg<br>1 min de descanso entre as séries	12–13 na Escala de Borg	Técnica de facilitação neuromuscular progressiva

FONTE: O autor (2019).

Os exercícios de equilíbrio serão compostos por diferentes exercícios enfatizando posturas dinâmicas, com movimentos do cotidiano ou que causem perturbações do equilíbrio. Exercícios estáticos serão compostos por posição unipodal, posição semi tandem, posição tandem (hálux do pé que está atrás tocando o calcâneo do pé da frente), ficar de pé sobre os calcanhares. O tempo máximo em cada posição estática será de 30 segundos. Os exercícios dinâmicos serão compostos por deslocamentos para frente, para trás e lateralmente em planti e dorsi flexão e em posição tandem, transferências de uma cadeira para outra, passar por cima de objetos, subir e descer degraus devagar e girar.

A intensidade será aumentada com redução da base de apoio (pés); redução de informações sensoriais (visual e vestibular); ou perturbação do centro de massa (por exemplo, segurando um objeto pesado em um lado enquanto mantém o equilíbrio, em pé em uma perna enquanto levanta a outra perna atrás do corpo, ou inclinando-se o máximo possível sem cair ou mover os pés). Mais informações sobre os exercícios de equilíbrio no Quadro 4.

QUADRO 4 - PLANEJAMENTO SEMANAL DOS EXERCÍCIOS DE EQUILÍBRIO

**Semana**	**Semana 1-3**	**Semana 4-6**	**Semana 7-9**	**Semana 10-12**
**Volume**	1–2 séries<br>4 a 5 exercícios enfatizando posturas estáticas e dinâmicas			
**Intensidade**	Com redução da base de apoio	Superfície instável (colchonete)	Redução da informação sensorial	Manipulando objetos
**Exercícios**	Exercícios estáticos: posição unipodal; semi tandem; posição tandem e ficar de pé sobre os calcanhares + manipulando objeto (ex: bola – jogando para cima, trocando de mão, jogar e bater palma).<br><br>Exercícios dinâmicos: caminhada para trás; caminhada formato do número 8; caminhada lateral; caminhada dorsiflexão; caminhada plantiflexão; sentar e levantar da cadeira; transferências de uma cadeira para outra; passar por cima de objetos; subir e descer degraus devagar; girar; em pé em uma perna enquanto levanta a outra perna atrás do corpo, ou inclinando-se o máximo possível sem cair ou mover os pés; deslocamento lateral com agachamento/adução e abdução de quadril.			

FONTE: O autor (2019).

Os exercícios de força muscular serão compostos por exercícios que envolvem os principais grupos musculares requisitados para a realização das atividades diárias, como marcha, levantar e sentar, subir escadas. Serão desenvolvidos exercícios dos dorsiflexores e plantiflexores do tornozelo, flexores e extensores dos joelhos e quadril, exercícios para glúteos, adutores e abdutores de quadril. Exercícios para abdômen e membros superiores completarão o treinamento. Serão realizadas de 2 a 3 séries de 08 a 12 repetições em cada exercício com intervalo de 1 minuto, com intensidade controlada pela percepção subjetiva de esforço a partir da escala de Borg (6-20). O planejamento do programa de exercício de força está apresentado no Quadro 5.

A intensidade das sessões será mensurada pela taxa de esforço percebido a partir da escala de Borg (6-20), em diferentes momentos da sessão nos exercícios de força, a fim de caracterizar a intensidade e não como monitoramento. Será realizada a explicação da escala, seguida de familiarização. A sobrecarga dos exercícios de força será determinada pela execução de 8 a 10 repetições do exercício, com material resistido (caneleira), de boa qualidade antes da fadiga.

QUADRO 5 - PLANEJAMENTO SEMANAL DOS EXERCÍCIOS DE FORÇA

**Semana**	**Semana 1-3**	**Semana 4-6**	**Semana 7-9**	**Semana 10-12**
**Volume**	3 séries de 12 repetições, intervalo de 1 minuto	3 séries de 10 repetições, intervalo de 30 segundos	3 séries de 8 repetições, intervalo de 1 minuto	3 séries de 8 repetições, intervalo de 30 segundos
**Sobrecarga**	Familiarização sem sobrecarga e início da progressão da carga	Progressão da sobrecarga: execução de boa qualidade antes da fadiga		
**Intensidade (PSE)**	Um pouco intenso a intenso (13-15)	Pesado a muito pesado (15–17)	Pesado a muito pesado (15–17)	Pesado a muito pesado (15–17)
**Exercícios**	Divisão das sessões por grupamento muscular:<br>Segunda-feira com ênfase nos músculos do quadríceps e panturrilha;<br>Quarta-feira com ênfase nos membros superiores e abdômen;<br>Sexta-feira com ênfase nos músculos posteriores e adutores/abdutores da coxa.<br>Exercícios: Extensão de joelho; Flexão de joelho; Extensão de quadril; Flexão de quadril; Adução de quadril; Abdução de quadril; Plantiflexão de tornozelo; Dorsiflexão de tornozelo; Glúteos. Exercícios complementares para membros superiores			

FONTE: O autor (2019).

Os exercícios de marcha serão compostos por deslocamentos na velocidade habitual e máxima, marcha estacionária com e sem elevação de joelhos e atividades envolvendo uma escada de agilidade. A escada de agilidade consiste em 10 quadrados (25 cm cada um) dispostos em linha reta. Os voluntários serão instruídos a andar de uma extremidade da escala à outra, de acordo com um padrão fornecido (Figura 2). Quando os voluntários

chegarem ao final da escada, serão instruídos a retornar às suas posições de partida, andando normalmente fora da escada, em seguida, iniciar o próximo padrão. Serão incluídos padrões de passos para frente, para trás, lateral e em diagonal. A progressão do exercício será realizada por meio da dificuldade dos padrões; pela diminuição da base de apoio, como andar nas pontas dos pés ou com os calcanhares; e pela realização de tarefa cognitiva simultaneamente, como nomear animais e contagem regressiva. Cada padrão será repetido de 4 a 10 vezes por sessão.

Os voluntários serão encorajados a se concentrar para a realizar com sucesso cada padrão de passos. A cadência do passo não será determinada, podendo ser realizada no ritmo preferido para cada participante. Os exercícios da escala de agilidade foram baseados no estudo de Shigematsu et al (2008).

FIGURA 2 – EXERCÍCIOS DE MARCHA NA ESCADA DE AGILIDADE

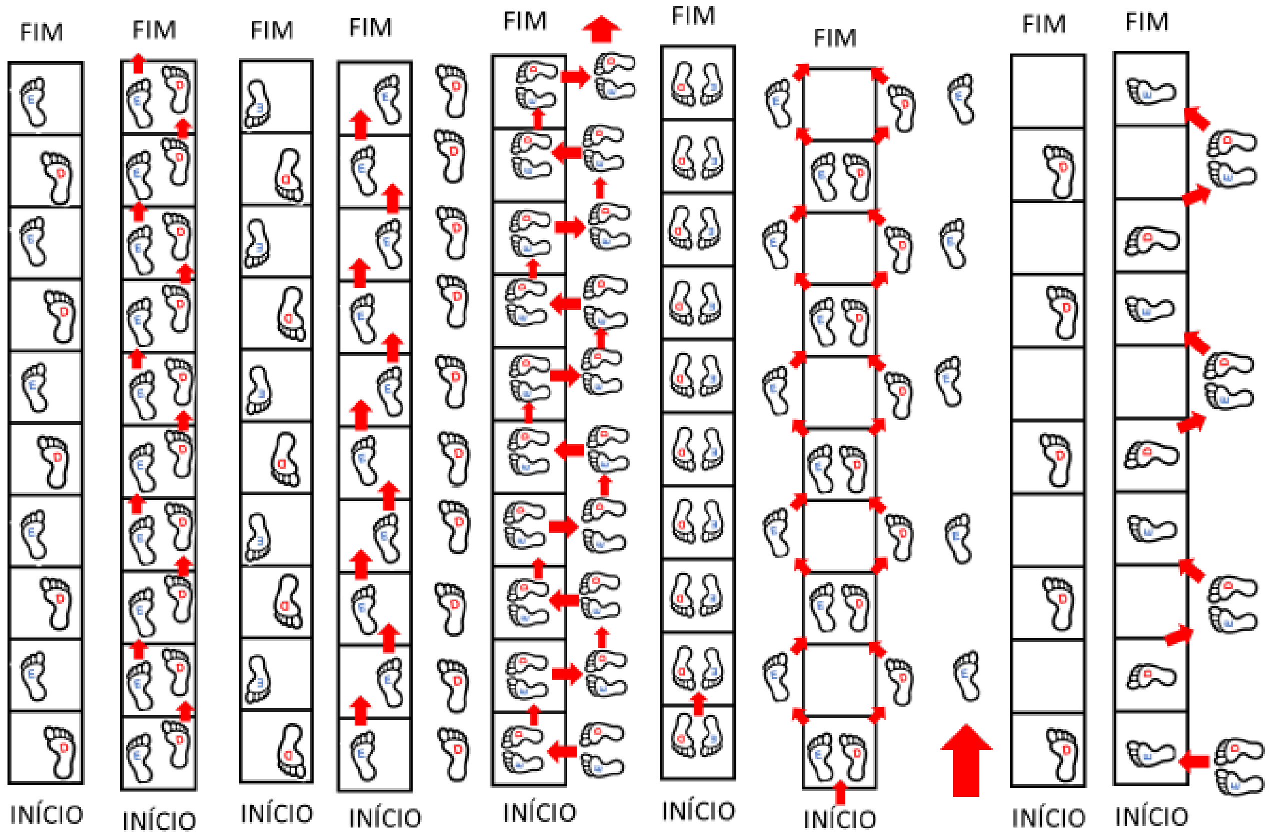

FONTE: O autor (2019).

Os exercícios de flexibilidade serão compostos pelo alongamento estático dos principais grupos musculares, como flexão e extensão de quadril e tronco, abdução,

adução dos membros superiores e inferiores. Cada posição será mantida por, pelo menos, 20 segundos. A intensidade será aumentada pela dificuldade progressiva do exercício, conforme tolerada pelo participante e utilizando o princípio da técnica de facilitação neuromuscular progressiva. A facilitação neuromuscular proprioceptiva envolve o máximo alongamento muscular possível, seguida de relaxamento e tentativa de maior alongamento, para finalmente manter a posição de alongamento máximo por pelo menos 20 segundos (SINGH, 2002).

Durante todas as sessões de treino a execução dos movimentos será priorizada e corrigida individualmente. Os voluntários serão estimulados o tempo todo a realizar os exercícios na maior velocidade possível. A intensidade dos exercícios será aumentada gradativamente, à medida que os participantes se adaptarem ao estímulo. O programa de exercício será dividido em quatro níveis de dificuldade: semana 1-3 fase, com introdução lenta de material resistido e os exercícios de equilíbrio foram realizados com redução da base de apoio; semana 4-6, com o incremento da carga do material resistido e os exercícios de equilíbrio foram realizados com apoio unipodal; semana 7-9, com incremento da carga e série e os exercícios de equilíbrio foram realizados com o apoio sobre superfícies instáveis, como colchonetes; semana 10-12,com incremento da carga e série e os exercícios de equilíbrio foram realizados com a redução da informação sensorial (visual e vestibular).

Os voluntários que realizarão sessões domiciliares (grupo GEPD e GED) receberão uma visita inicial no seu domicílio na qual serão informadas todas as orientações necessárias para a realização do programa com segurança e um manual com instruções para cada exercício, com espaço para registro das sessões realizadas e possíveis ocorrências (ANEXO 6). Além disso, receberam um kit de materiais contendo três pares de caneleiras (1, 2 e 4 kg) para proporcionar resistência aos exercícios de fortalecimento e uma escala de agilidade para os exercícios de marcha. Para controlar a aderência ao programa, os participantes registrarão os dias que realizaram o programa e receberão mensagens com incentivos para a realização dos exercícios em casa.

6.4 TRATAMENTO ESTATÍSITCO

Para análise dos dados coletados será utilizada a estatística descritiva, média e desvio-padrão. O teste de Shapiro-Wilk será aplicado para verificar a normalidade dos dados e o teste de Levene para a homogeneidade da amostra. Para os dados normais, será utilizada a comparação entre grupos e entre os períodos (pré e pós treinamento) por meio da ANOVA de modelo misto com post-hoc de Bonferroni. Quando observadas diferenças entre os grupos no período pré-treinamento, será aplicado uma análise de covariância (ANCOVA), com os valores iniciais como covariada e compararados os dados no pós testes desconsiderando as diferenças iniciais. O coeficiente de $p<0.05$ será adotado para determinar a significância dos dados. Os testes serão realizados por meio do software IBM SPSS Statistics versão 25.

7. Resultados Esperados do Estudo

Com a realização do presente estudo, espera-se encontrar, após o programa de exercícios físicos, um melhor entendimento sobre a condução de exercício físico na população idosa, como a identificação da proposta de condução do programa de exercício mais efetiva para o aumento de força muscular, funcionalidade e qualidade de vida de idosos; o desenvolvimento de um programa de exercício que poderá ser realizado tanto em domicílio como em grupo, facilitando barreiras como dependência do idoso para locomoção ao local de treinamento físico; a promoção e manutenção da independência na realização de atividade física, bem como a realização das atividades básicas da vida diária; a manutenção de um estilo de vida ativo após a finalização do programa de exercício físico. Adicionalmente, espera-se identificar em quanto tempo ocorre redução dos efeitos dos exercícios nos aspectos clínicos, funcionais e de qualidade de vida dos participantes que deixaram de praticar exercícios após a intervenção.

Além disso, o projeto contribuirá na formação de pesquisadores, pois fará parte da tese de doutorado da aluna Sabrine Nayara Costa (PPGED/UFPR) e de projeto de pesquisa de iniciação científica do curso de Educação Física da UFPR. Adicionalmente, espera-se contribuir com o conhecimento de profissionais da área da saúde acerca da

avaliação e prescrição de exercícios físicos para idosos, para que possam dar continuidade a projetos como este na prática profissional.

Os pesquisadores garantem que os dados dos resultados serão divulgados individualmente aos participantes da pesquisa.

8. Análise Crítica de Riscos e Benefícios

8.1 Quais os benefícios, diretos ou indiretos, para a população e a sociedade?

Os benefícios diretos desta pesquisa para a população estudada envolvem a participação em um programa de exercício físico planejado e controlado, que pode ser realizado tanto em domicílio como em grupo e, que pode promover efeitos positivos na manutenção e aumento da função física, o aumento da força e potência muscular de membros inferiores, melhorias no equilíbrio e no padrão de caminhar, consequentemente reduzindo o risco de quedas e aumentando a qualidade de vida dos participantes. Indiretamente, o presente estudo contribuirá com a formação dos profissionais e acadêmicos envolvidos em relação à prescrição de exercícios para idosos e seus efeitos. Ao final da pesquisa, os dados e resultados serão divulgados a todos os participantes e colaboradores, bem como em congressos e publicações científicas, contribuindo com o conhecimento científico na área.

8.2 Quais os riscos inerentes ou decorrentes da pesquisa?

É possível que os participantes experimentem algum desconforto em determinadas avaliações e exercícios, como risco de dores musculares de início tardio (até 48 horas após a prática) que são comuns quando se inicia a participação em um programa de exercício físicos. À medida que o corpo se adapta ao exercício estas dores não devem mais ocorrer. Há também a possibilidades de lesão muscular durante a prática, que serão minimizadas por período de aquecimento no início da sessão e relaxamento ao final da sessão. Além disso, é possível que o participante se sinta constrangido ao responder os questionários, porém, se isso ocorrer, o participante pode interromper a entrevista ou optar por não responder, sem acarretar nenhum prejuízo à sua participação no projeto.

8.3 Qual a possibilidade da ocorrência?

As dores musculares poderão ocorrer quando realizado ajuste de carga e/ou início de uma nova série de exercícios.

8.4 Quais as medidas para sua minimização e proteção do participante da pesquisa?

As sessões de exercícios serão precedidas de período de aquecimento e exercícios de alongamento e relaxamentos serão aplicados ao final de cada sessão. Em adição, haverá um período de adaptação ao programa de exercícios e as cargas serão individualizadas e sofrerão aumento progressivo ao longo do programa.

Caso ocorra constrangimento por parte do participante ao responder algum dos questionários, este poderá interromper a entrevista ou optar por não responder.

Todas as ações acima citadas contribuirão para a redução do risco de qualquer evento negativo durante o período do estudo, porém, caso ocorra, serão tomadas as devidas providências para que o participante seja atendido e tratado imediatamente.

9. Duração Total da Pesquisa | Cronograma

A pesquisa tem início previsto a partir da aprovação do CEP/SD e término em dezembro de 2021. O recrutamento e a realização dos programas de exercício físico serão realizados durante dois anos, conforme cronograma abaixo.

MÊS/ANOS	AÇÕES
**Janeiro/2020**	Divulgação do projeto
**Fevereiro/2020**	Mapeamento e recrutamento de participantes
**Março a Abril/2020**	Avaliações iniciais
**Maio a Julho/2020**	Intervenção com programa de exercício
**Agosto a Setembro/2020**	Avaliações finais
**Outubro/2020**	Avaliação de follow-up
**Novembro a Dezembro/2020**	Apresentação de resultados aos participantes
**Janeiro/2021**	Divulgação do projeto
**Fevereiro/2021**	Mapeamento e recrutamento de participantes

**Março a Abril/2021**	Avaliações iniciais
**Maio a Julho/2021**	Intervenção com programa de exercício
**Agosto a Setembro/2021**	Avaliações finais
**Outubro/2021**	Avaliação de follow-up
**Novembro/2021**	Apresentação de resultados aos participantes
**Dezembro/ 2021**	Análise de dados total

10. Critérios para Suspender ou Encerrar a Pesquisa

Não atingir o número de voluntários necessários para a realização do estudo.

11. Local de Realização da Pesquisa

As avaliações pré e pós-intervenção serão realizadas no Centro de Estudos do Comportamento Motor (CECOM) na UFPR e os programas de exercício serão realizados na sala de atividades rítmicas do Departamento de Educação Física (DEF/UFPR). No caso do grupo de exercícios domiciliares, as atividades serão realizadas no domicílio e terão acompanhamento mediante contato telefônico e visitas domiciliares realizadas pelos pesquisadores.

11. Demonstrativo da Existência de Infra-Estrutura

As avaliações e o programa de intervenção serão realizados nas dependências do Departamento de Educação Física (UFPR). Os instrumentos de avaliação e materiais para a realização do programa de exercício pertencem ao Centro de Estudos do Comportamento Motor (CECOM) do DEF/UFPR.

12. Propriedade das Informações

As informações referentes aos dados coletados durante a pesquisa estarão sobre a guarda e sigilo do pesquisador responsável, Prof. Dr. Paulo Cesar Barauce Bento.

14. Informações Relativas ao Participante da Pesquisa e Características da População a Ser Estudada

Participarão do estudo homens e mulheres com idade igual ou superior a 60 anos, residentes na comunidade, sem distinção em relação a cor, raça, etnia e classe social.

15. Grupos Vulneráveis

Não se aplica.

16. Fontes de Material da Pesquisa

Todo o material listado será usado para os propósitos dessa pesquisa:

FINALIDADE	MATERIAL	OBTENÇÃO
Avaliações	*Notebook*	DEF/UFPR
Avaliações	Dinamômetro isocinético Biodex System (Biodex Medical Systems Inc., Shirley, NY, USA)	DEF/UFPR
Avaliações	Tapete Zeno Walkway e software ProtoKinetics Movement Analysis (PKMAS)	DEF/UFPR
Avaliações	Sistema de análise tridimensional do movimento (VICON)	DEF/UFPR
Avaliações	Plataforma de força (AMTI, modelo OR-06, USA)	DEF/UFPR
Avaliações	Microsoft Kinect	DEF/UFPR
Avaliações	Equipamento de ultrassom (Konica Minolta Medical Imaging Inc Newark-Pompton Turnpike,	DEF/UFPR

	Wayne, NJ, USA)	
Avaliações	Eletromiógrafo Trigno Wireless, Delsys, USA;	DEF/UFPR
Avaliações	Estimulador elétrico modelo Neuro IOM – Neurosoft®	DEF/UFPR
Avaliações	Cadeira	DEF/UFPR
Avaliações	Cone	DEF/UFPR
Avaliações	Dinamômetro de preensão manual	DEF/UFPR
Avaliações	Sala de rítmicas	DEF/UFPR
Avaliações	Caneleiras	DEF/UFPR

17. Planos para o Recrutamento do Participante da Pesquisa

Os participantes serão recrutados a partir da divulgação do projeto pelos meios de comunicação da UFPR, mídias sociais e jornais locais. Os interessados poderão entrar em contato com os pesquisadores para obter informações sobre a pesquisa e serão convidados a participar de uma reunião inicial em que serão apresentados os objetivos e procedimentos. Aqueles que aceitarem participar voluntariamente, assinarão o Termo de Consentimento Livre e Esclarecido.

18. Critérios de Inclusão e Exclusão

Serão incluídos no estudo homens e mulheres com idade igual ou superior a 60 anos, que sejam independentes na realização das atividades diárias básicas, sem comprometimento cognitivo determinado por meio do Mini Exame do Estado Mental (MEEM) com pontuação estratificada por escolaridade, que não estejam participando de nenhum programa de treinamento ou exercício físico regular e sistematizado há pelo menos 6 meses e que não se enquadrem em nenhum critério de exclusão.

Não serão incluídos idosos com limitações físicas ou motoras que impossibilitem a realização dos testes funcionais, com histórico recente de derrame, insuficiência cardíaca, que utilizem marcapasso, que não faça controle de condições médicas instáveis (ex:

diabetes mellitus e hipertensão), com doença neurológica, com osteoporose grave com histórico de duas ou mais fraturas, que não andem de forma independente e que façam uso de órtese. Após a seleção dos participantes de acordo com os critérios supracitados, será solicitado aos idosos um atestado médico com a liberação para realização de atividade física, e deste modo, serão excluídos os idosos que apresentem contraindicações absolutas para a participação no protocolo de exercício proposto.

19. Medidas de Proteção ou Minimização de Qualquer Risco Eventual

Durante a realização dos testes ou a participação nas aulas é possível que o participante experimente algum desconforto relacionado a dores musculares ou articulares, que são comuns quando se pratica atividade física, principalmente no início, quando seu corpo ainda não está acostumado com a nova atividade. No entanto, à medida que o corpo se adapta aos exercícios essas dores não devem mais ocorrer. Ao participar de um treinamento físico o participante corre o risco de em algum momento machucar-se ou sentir dores no corpo devido ao esforço. As possíveis dores musculares ou desconfortos serão minimizadas pela realização de período de aquecimento adequado e dosagem individual da carga de exercícios com base nas avaliações iniciais. Para garantir adaptação ao exercício, as cargas serão aumentadas gradativamente a cada 3 semanas de exercícios. A percepção subjetiva de esforço (escala de Borg) será utilizada como forma auxiliar de acompanhamento da adaptação ao programa. Se necessário, o serviço de atendimento médico do convênio com a Plus Santé será acionado.

20. Previsão de Ressarcimento de Gastos aos Participantes da Pesquisa

As despesas necessárias para a realização da pesquisa não de responsabilidade dos participantes, inclusive o deslocamento do participante, sendo que eles não receberão nenhum valor em dinheiro.

21. Referências Bibliográficas

ABE, T.; KUMAGAI, K.; BRECHUE, W. F. Fascicle length of leg muscles is greater in sprinters than distance runners. **Applied Sciences**, v. 1, n. 14, p. 1125–1129, 2000.

AMERICAN COLLEGE OF SPORTS MEDICE. Exercise and physical activity for older adults. **Medicine and Science in Sports and Exercise**, v. 41, n. 7, p. 1510–1530, 2009.

AMERICAN THORACIC SOCIETY. ATS statement: Guidelines for the six-minute walk test. **American Journal of Respiratory and Critical Care Medicine**, v. 166, n. 1, p. 111–117, 2002.

ARRIETA, H. et al. A multicomponent exercise program improves physical function in long-term nursing home residents: A randomized controlled trial. **Experimental Gerontology**, v. 103, n. October 2017, p. 94–100, 2018.

BAUMAN, A. et al. Updating the Evidence for Physical Activity: Summative Reviews of the Epidemiological Evidence, Prevalence, and Interventions to Promote “active Aging”. **Gerontologist**, v. 56, n. April, p. S268–S280, 2016.

BEAUCHET, O. et al. Stops walking when talking: A predictor of falls in older adults? **European Journal of Neurology**, v. 16, n. 7, p. 786–795, 2009.

BERTOLUCCI, P. H. et al. O Mini-Exame do Estado Mental em uma população geral. **Arquivos de Neuro-Psiquiatria**, v. 52, n. 1, p. 1–7, 1994.

BLAZEVICH, A. J.; SHARP, N. C. C. Understanding muscle architectural adaptation: Macro- and micro-level research. **Cells Tissues Organs**, v. 181, n. 1, p. 1–10, 2005.

BRADY, A. O.; STRAIGHT, C. R.; EVANS, E. M. Body composition, muscle capacity, and physical function in older adults: An integrated conceptual model. **Journal of Aging and Physical Activity**, v. 22, n. 3, p. 441–452, 2014.

BYRNE, C. et al. Ageing , Muscle Power and Physical Function : A Systematic Review and Implications for Pragmatic Training Interventions. **Sports Medicine**, 2016.

CADORE, E. L. et al. Multicomponent exercises including muscle power training enhance muscle mass, power output, and functional outcomes in institutionalized frail nonagenarians. **Age**, v. 36, n. 2, p. 773–785, 2014.

CICONELLI, R. et al. Tradução para a língua portuguesa e validação do questionário genérico de avaliação de qualidade de vida SF-36 (Brasil SF-36). **Revista Brasileira de Reumatologia**, v. 39, n. 3, p. 8, 1999.

CLARK, B. C.; MANINI, T. M. Functional consequences of sarcopenia and dynapenia in the elderly. **Curr Opin Clin Nutr Metab Care**, v. 13, n. 3, p. 271–276, 2010.

COSTA, S. N.; VIEIRA, E. R.; BENTO, P. C. B. Effects of Home- and Center-Based Exercise Programs on the Strength , Function , and Gait of Prefrail Older Women : A Randomized Control Trial. **Journal of Aging and Physical Activity**, p. 1–10, 2019.

CRESS, M. E. et al. Best Practices for Physical Activity Programs and Behavior Counseling in Older Adult Populations. **Journal of Aging and Physical Activity**, v. 13, n. 1, p. 61–74, 2005.

DALY, R. M. et al. Effectiveness of dual-task functional power training for preventing falls in older people: study protocol for a cluster randomised controlled trial. **Trials**, v. 16, n. 1, p. 1–15, 2015.

FERNANDES, A. DE A.; MARINS, J. C. B. Test of hand grip strength: a methodological analysis and normative data in athletes. **Fisioter Mov**, v. 24, n. 3, p. 567–578, 2011.

FOLSTEIN, M. F.; FOLSTEIN, S. E.; MCHUGH, P. R. “Mini-mental state”. A practical method for grading the cognitive state of patients for the clinician. **Journal of Psychiatric Research**, v. 12, n. 3, p. 189–198, 1975.

FRAGALA, M. S.; KENNY, A. M.; KUCHEL, G. A. Muscle Quality in Aging: a Multi-Dimensional Approach to Muscle Functioning with Applications for Treatment. **Sports Medicine**, v. 45, n. 5, p. 641–658, 2015.

FRIED, L. P. et al. Frailty in older adults: Evidence for a phenotype. **Journals of Gerontology: Medical Sciences**, v. 56, n. 3, p. M146–M156, 2001.

GILL, T. M. et al. A program to prevent functional decline in physically frail, elderly persons who live at home. **The New England journal of medicine**, v. 347, n. 14, p. 1068–74, 2002.

GOMES, G. DE C. et al. Gait performance of the elderly under dual-task conditions: Review of instruments employed and kinematic parameters. **Revista brasileira de g**, v. 1, n. 1, p. 165–182, 2016.

GUEDES, D. P. **Manual Prático para avaliação em Educação Física**. 1. ed. São Paulo: 1, 2006.

GURALNIK; et al. A Short Physical Performance Battery assessing lower extremity function: association with self-reported. **J Gerontol**, v. 49, n. 2, p. 85–94, 1994.

HENDELMAN et al. Validity of accelerometry for the assessment of moderate intensity physical activity in the field. **Medicine & Science in Sports & Exercise**, v. 32, n. 9, p. 9, 2000.

HERMENS, H. J. et al. Development of recommendations for SEMG sensors and sensor placement procedures. **Journal of Electromyography and Kinesiology**, v. 10, n. 1, p. 361–374, 2000.

IBGE. Censo demográfico 2010. **Instituto Brasileiro de Geografia e Estatística**, 2010.

KNIGHT, C. A.; KAMEN, G. Adaptations in muscular activation of the knee extensor muscles with strength training in young and older adults. **Journal of Electromyography and Kinesiology**, v. 11, n. 6, p. 405–412, 2001.

LACROIX, A. et al. Effects of Supervised vs . Unsupervised Training Programs on Balance and Muscle Strength in Older Adults : A Systematic Review and Meta-Analysis. **Sports Medicine**, v. 1, n. 1, p. 21, 2017.

LEBRÃO, M. L.; DUARTE, Y. A. O. **O projeto SABE no município de São Paulo: uma abordagem inicial**. 1. ed. Brasília: 1, 2003.

LEXELL, J. Strength Training and Muscle Hypertrophy in Older Men and Women. **Geriatr Rehabil**, v. 15, n. 3, p. 41–46, 2000.

LIM, J. P. et al. Inter-muscular adipose tissue is associated with adipose tissue inflammation and poorer functional performance in central adiposity. **Archives of Gerontology and Geriatrics**, v. 81, p. 1–7, 2019.

LOK, N.; LOK, S.; CANBAZ, M. The effect of physical activity on depressive symptoms and quality of life among elderly nursing home residents: Randomized controlled trial. **Archives of Gerontology and Geriatrics**, v. 70, p. 92–98, 2017.

LUSTOSA et al. Tradução e adaptação transcultural do Minnesota Leisure Time Activities Questionnaire em idosos. **Geriatria & Gerontologia**, v. 5, n. 2, p. 57–65, 2011.

MANINI, T. Development of Physical Disability in Older Adults. **Current Aging Sciencee**, v. 4, n. 3, p. 184–191, 2012.

MAYR, S. et al. A short tutorial of GPower. **Tutorials in Quantitative Methods for Psychology**, v. 3, n. 2, p. 51–59, 2007.

MCBEAN, A. L. et al. Standing Balance and Spatiotemporal Aspects of Gait Are Impaired Upon Nocturnal Awakening in Healthy Late Middle-Aged and Older Adults. **Journal of Clinical Sleep Medicine**, v. 12, n. 11, p. 1477–1486, 2016.

MCPHEE, J. S. et al. Physical activity in older age: perspectives for healthy ageing and frailty. **Biogerontology**, v. 17, n. 3, p. 1–14, 2016.

MIRANDA, G.; MENDES, A.; SILVA, A. Population aging in Brazil: corrent and futere

social challenges and consequences. **Revista Brasileira de Geriatria e Gerontologia**, v. 19, n. 3, p. 195–198, 2016.

MITCHELL, W. K. et al. Sarcopenia, dynapenia, and the impact of advancing age on human skeletal muscle size and strength; a quantitative review. **Frontiers in Physiology**, v. 3 JUL, n. July, p. 1–18, 2012.

NARICI, M. V.; MAGANARIS, C. N. Adaptability of elderly human muscles and tendons to increased loading. **Journal of Anatomy**, v. 208, n. 4, p. 433–443, 2006.

NELSON, J. K.; THOMAS, J. R. **Métodos de Pesquisa em Atividade Física**. 6. ed. Brasil: 1, 2012.

PETERS, M. Footedness: Asymmetries in Foot Preference and Skill and Neuropsychological Assessment of Foot Movement. **Psychological Bulletin**, v. 103, n. 2, p. 179–192, 1988.

PODSIADLO, D.; RICHARDSON, S. The Timed ”Up & Go”: A Test of Basic Functional Mobilitv for Frail Elderly Persons. **Journal of American Geriatrics Society**, p. 142–148, 1991.

RADLOFF, L. S. The CES-D Scale : A Self-Report Depression Scale for Research in the General Population. **Applied Psychological Measurement**, v. 1, n. 3, p. 385–401, 1977.

REEVES, N. D.; MAGANARIS, Æ. C. N.; NARICI, M. V. Ultrasonographic assessment of human skeletal muscle size. **European Journal of Applied Physiology**, v. 91, p. 116–118, 2004.

RIKLI, R. E.; JONES. **Sênior Fitness Test Manual**. 5 edition ed. São Paulo: Manole, 2011.

ROAD, R. **Biodex Medical Systems**, 2000.

ROZAND, V. et al. Assessment of Neuromuscular Function Using Percutaneous Electrical Nerve Stimulation. **Journal of Visualized Experiments**, n. 103, p. 1–11, 2015.

RUBENSTEIN, L. Z. Falls in older people: Epidemiology, risk factors and strategies for prevention. **Age and Ageing**, v. 35, n. SUPPL.2, p. 37–41, 2006.

SELVA RAJ, I.; BIRD, S. R.; SHIELD, A. J. Ultrasound Measurements of Skeletal Muscle Architecture Are Associated with Strength and Functional Capacity in Older Adults. **Ultrasound in Medicine and Biology**, v. 43, n. 3, p. 586–594, 2017.

SINGH, M. A. F. Exercise Comes of Age Rationale and Recommendations .pdf. **Journal of Gerontology**, v. 57, n. 5, p. 262–282, 2002.

SPIRDUSO. **Dimensões Físicas do Envelhecimento**. 5. ed. Brasil: 1, 2005.

STATHI, A.; MCKENNA, J.; FOX, K. R. Processes associated with participation and adherence to a 12-month exercise programme for adults aged 70 and older. **Journal of Health Psychology**, v. 15, n. 6, p. 838–847, 2010.

TEIXEIRA, J. **Associação entre os componentes da sobrecarga de treinamento e alterações na função, composição e arquiteratura muscula no atleta master de corrida**. [s.l: s.n.].

TIELAND, M.; TROUWBORST, I.; CLARK, B. C. Skeletal muscle performance and ageing. **Journal of Cachexia, Sarcopenia and Muscle**, v. 9, n. 1, p. 3–19, 2018.

UNITED NATIONS. **World Population Ageing**. [s.l: s.n.].

VALLABHAJOSULA, S. et al. Concurrent Validity of the Zeno Walkway for Measuring Spatiotemporal Gait Parameters in Older Adults. **Journal of Geriatric Physical Therapy**, v. 0, n. 0, p. 1, 2017.

VERAS, R. Envelhecimento populacional contemporâneo: demandas, desafi os e inovações - Population aging today: demands, challenges and innovations. **Revista de Saúde Pública**, v. 43, n. 3, p. 548–554, 2009.

WARE, J. E.; GANDEK, B. Overview of the SF-36 Health Survey and the International Quality of Life Assessment (IQOLA) Project. **J Clin Epidemiol**, v. 51, n. 11, p. 903–912, 1998.

WEI, N.; NG, G. Y. F. The effect of whole body vibration training on quadriceps voluntary activation level of people with age-related muscle loss (sarcopenia): A randomized pilot study. **BMC Geriatrics**, v. 18, n. 1, p. 1–6, 2018.

WU, S.; PARK, K.-S.; MCCORMICK, J. B. Effects of Exercise Training on Fat Loss and Lean Mass Gain in Mexican-American and Korean Premenopausal Women. **International Journal of Endocrinology**, v. 2017, p. 1–7, 2017.

22. Anexos

ANEXO 1 - ANAMNESE

FICHA DE AVALIAÇÃO

Avaliador: ____________________________ CÓDIGO: ________ DATA: ___/____/_____

Data de nascimento: ____ Idade: ________

Endereço: ____

Contato telefônico: ____

Massa corporal: ______________ kg Estatura: __________ m IMC: _______ kg/m²

Circunf. Abdominal: __________ cm Comprimento da perda: D ______ E ________

NOME: ____

Mão que escreve: ____________ Perna dominante: _________ (teste: subir escada)

**Escolaridade:**<br>( ) Analfabeto<br>( ) 1-4 anos<br>( ) 5-8 anos<br>( ) >8 anos<br>( ) Superior incomp.<br>( ) Superior completo<br>( ) Pós-graduação	**Situação conjugal**<br>( ) Casado<br>( ) Divorciado<br>( ) Separado<br>( ) Viúvo<br>( ) Solteiro	**Profissão:**<br>_________<br>**Ocupação**<br>( ) Aposentado com outra ocupação<br>( ) Aposentado sem outra ocupação<br>( ) Trabalhos domésticos<br>( ) Trabalho fora do domicilio	**Renda**<br>( ) Aposentadoria<br>( ) Pensão<br>( ) Mesada dos filhos<br>( ) Aluguel<br>( ) Trabalho<br>Outras_____________
**Local de residência**<br>( ) Casa térrea<br>( ) Casa duplex<br>( ) Apartamento<br>( ) ILP<br>Outros________	**Residência**<br>( ) Sozinho<br>( ) Filhos<br>( ) Outros familiares<br>( ) Cuidadores<br>Outros_______	**Religião**<br>( ) Católica<br>( ) Evangélica<br>( ) Espírita<br>( ) Budista<br>Outra____________	**Etnia**<br>( ) Negra<br>( ) Branca<br>( ) Parda<br>( ) Amarela<br>Outra_______________
VISÃO<br>( ) Visão normal<br>( ) Déficit visual<br>( ) Usa corretores	AUDIÇÃO<br>( ) normal<br>( ) Déficit auditivo	CIRURGIAS<br>( ) Sim<br>( ) Não<br>Qual?____________	Uso de órtese: ____________________<br>Uso de prótese:

<table>
<tr><td></td><td>( )Usa corretores</td><td></td><td>____________________</td></tr>
<tr><td>DOENÇAS
( ) Hipertensão
( ) Diabetes
( ) Osteoporose
( ) Dislipidemia
( ) Art
( ) Artrose
( ) Problema na tireoide
( ) Visão/Cataratas
( ) Deficiência Auditiva
( ) Incontinência urinári</td><td colspan="2">Medicamentos:
Número de medicamentos:
( ) Hormônio: ____________________
( ) Diurético: _____________________
( ) Antidepressivo: _________________
( ) Pressão Arterial: _______________
( ) Anti-inflamatórios: ______________
( ) Analgésicos: ___________________
( ) Cardiovasculares: _______________
( ) Vitaminas: _____________________
( ) Suplementos: __________________
( ) Outros____________________________
__________________________________</td><td>Frequência:
____
____
____
____
____
____
____
____
____
________________</td></tr>
<tr><td>Histórico de cardiopatia na família?
( )Sim ( ) Não
Quem?
_______________</td><td colspan="2">Tabagismo? ( )Sim ( ) Não
Frequência: ________________
Bebidas alcoólicas? ? ( )Sim ( ) Não
Frequência: _______
Atividade física atual? ? ( )Sim ( ) Não</td><td>Histórico de cardiopatia na família?
( )Sim ( ) Não
Quem?
_______________</td></tr>
</table>

Atividade	Tempo de prática	Frequência semanal

ANEXO 2 – MINI EXAME DO ESTADO MENTAL

Avaliador: ____________________ CÓDIGO: ______________ DATA: ___/____/_____

Orientação Temporal Espacial

1. Qual é o dia da semana? .... (1)
   Dia do mês? .... (1)
   Mês? .... (1)
   Ano? .... (1)
   Hora aproximada? .... (1)
2. Onde estamos?
   Local? .... (1)
   Instituição (casa,rua)? .... (1)
   Bairro? .... (1)
   Cidade? .... (1)
   Estado? .... (1)

Registro

1. Mencione 3 palavras levando 1 segundo para cada uma. Peça ao paciente para repetir as 3 palavras que você mencionou. Estabeleça um ponto para casa resposta correta.
   - Vaso, carro, tijolo
   .... (3)

Atenção e cálculo

"Sete" seriado. Dê 1 ponto para cada correto. Interrompa após 5 perguntas. Alternativamente soletre a palavra MUNDO de trás para frente

.... (5)

Lembrança (memória de evocação)

Pergunte o nome das três palavras aprendidas na questão 2. Estabeleça um ponto para cada resposta correta.

.... (3)

Linguagem

5. Aponte para um lápis e um relógio. Faça o paciente dizer o nome desses objetos conforme você os aponta

.... (2)

6. Faça o paciente repetir "nem aqui, nem ali, nem lá".

.... (1)

7. Faça o paciente seguir o comando de 3 estágios "Pegue o papel com a mão direita. Dobre o papel ao meio. Coloque o papel na mesa".

.... (3)

8. Faça o paciente ler e obedecer ao seguinte: FECHE OS OLHOS

.... (1)

9. Faça o paciente escrever uma frase de sua própria autoria. (A frase deve conter um sujeito e um objeto e fazer sentido).
(Ignore erros de ortografia ao marcar o ponto).

.... (1)

10. Copie o desenho abaixo.
Estabeleça um ponto se todos os lados e ângulos forem preservados e se os lados da interseção formarem um quadrilátero

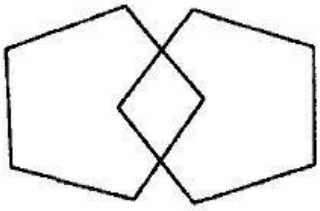

.... (1)

ANEXO 3 – IDENTIFICAÇÃO DO FENÓTIPO FRAGILIDADE

Avaliador: __________________ **CÓDIGO:** ______________ **DATA:** ___/____/_____

Nome: ____
Data de nascimento: ______/______/_______ **Idade**: __________ anos
Estado civil: ( ) solteiro ( ) casado ( ) divorciado ( ) viúvo
Telefone: ___________________________ **Telefone celular:** ______________________

TRIAGEM DO FENÓTIPO FRAGILIDADE

Antropometria
Massa corporal: .... kg **Estatura**: .... m **IMC**: .... kg/m²
Circunf. Abdominal: .... **Compr. Perna**: **D** .... mm **E** .... mm

1. **Perda de peso não intencional**

4,5 Kg ou 5% do peso corporal no último ano ( ) Sim ( ) Não

2. **Preensão Manual – Lado Direito**

.... kgf
.... kgf
.... kgf

Homens: ( ) ≤ 21 kgf
Mulheres:
( ) IMC ≤ 24 FP ≤ 14
( ) IMC 24.1-27 FP ≤ 15
( ) IMC 27.1-31 FP ≤ 17
( ) IMC > 31 FP ≤ 14

3. **Exaustão/Fadiga**

a) Senti que tive que fazer esforço para dar conta das minhas tarefas habituais?

(Zero) Nunca ou Raramente (2) As vezes (3) Maioria das vezes ou sempre.

b) Não consegui levar a diante minhas coisas?

(Zero) Nunca ou Raramente (2) As vezes (3) Maioria das vezes ou sempre.

4. **Velocidade Da Marcha - Teste de 4 metros**

( ) sim ( ) não

.... s
.... s
.... s

Homens:
( ) altura ≤ 169 cm ≥ 5 segundos
( ) altura > 169 cm ≥ 6 segundos
Mulheres:
( ) altura ≤ 153 cm ≥ 6 segundos
( ) altura > 153 cm ≥ 5 segundos

5. Baixa Atividade Física – Minnesota Leisute Time Activity

Gasto energético por semana inferior: Homens: a 383 kcal e Mulheres: 270 kcal

QUESTIONÁRIO MINNESOTA DE ATIVIDADES FÍSICAS, ESPORTE E LAZER

**A ser completado pelo participante**	**Você realizou esta atividade?**		**1ª semana**	**2ª semana**	**Tempo por ocasião**
**Atividade**	**não**	**sim**	**(média de x última semana)**	**(média de x penúltima semana)**	**(minutos)**
**Seção A: Caminhada**					
010 Caminhada recreativa					
020 Caminhada para o trabalho					
030 Uso de escadas quando o elevador está disponível					
040 Caminhada ecológica					
050 Caminhada com mochila					
060 Alpinismo/escalando montanhas					
115 Ciclismo recreativo/por prazer					
125 Dança – salão, quadrilha e/ou discoteca, danças regionais					
135 Dança/ginástica – aeróbia, balé					
140 Hipismo/andando a cavalo					
**Seção B: Exercício de condicionamento**					
150 Exercícios domiciliares					
160 Exercício em clube/em academia					
180 Combinação de caminhada/corrida leve					
200 Corrida					
210 Musculação					
**Seção C: Atividades aquáticas**					
220 Esqui aquático					
235 Velejando em competição					
250 Canoagem ou remo recreativo					
260 Canoagem ou remo em competição					
270 Canoagem em viagem de acampamento					
280 Natação em piscina (pelo menos 15					

metros)					
295 Natação na praia					
310 Mergulho autônomo					
320 Mergulho livre – snorkel					
**Seção D: Atividades de inverno**					
340 Esquiar na montanha					
350 Esquiar no plano					
360 Patinação no gelo ou sobre rodas					
370 Trenó ou tobogã					
**Seção E: Esportes**					
390 Boliche					
400 Voleibol					
410 Tênis de mesa					
420 Tênis individual					
430 Tênis de duplas					
480 Basquete sem jogo (bola ao cesto)					
490 Jogo de basquete					
500 Basquete como juiz					
520 Handebol					
530 Squash					
540 Futebol					
Golf					
070 Dirigir carro de golfe					
080 Caminhada, tirando os tacos do carro					
090 Caminhada carregando os tacos					
**Seção F: Atividades no jardim e na horta**					
550 Cortar a grama dirigindo um carro de cortar grama					
560 Cortar a grama andando atrás do cortador de grama motorizado					
570 Cortar a grama empurrando o cortador de grama manual					
580 Tirando o mato e cultivando o jardim/horta					
590 Afofar, cavando e cultivando a terra no jardim e na horta					
600 Trabalho com ancinho na grama					

610 Remoção de neve/terra com pá					
**Seção G: Atividades de reparos domésticos**					
620 Carpintaria em oficina					
630 Pintura interna de casa ou colocação de papel de parede					
640 Carpintaria do lado de fora da casa					
650 Pintura exterior de casa					
**Seção H: Pesca**					
660 Pesca na margem do rio					
670 Pesca em correnteza com botas					
**Seção I: Outras atividades (descrever)**					

ANEXO 4 - TESTES FUNCIONAIS

Avaliador: ____________________ CÓDIGO: ______________ DATA: ___/____/_____

**Testes Funcionais**	
**Sentar e levantar da cadeira (SPPB)**	**TEMPO: ______:_______**
**Equilíbrio (SPPB)**	**Unipodal: ______________seg**<br>**Semi-tandem: ___________seg**<br>**Tandem: _______________seg**
**Levantar e caminhar cronometrado (TUG)**	**1 - TEMPO: ______:_______**<br>**2 - TEMPO: ______:_______**
**Sentar e alcançar**	____________________ cm
**Alcançar atrás das costas**	____________________ cm
**Flexão de antebraço**	_________________ repetições
**Teste de caminhada de 6 minutos**	_________________________ metros

ANEXO 5 - MEDICAL OUTCOMES STUDY 36 – SF-36

CÓDIGO

<u>SF – 36 PEQUISA EM SAÚDE</u>

Instruções: Esta pesquisa questiona você sobre sua saúde. Estas informações nos manterão informados de como você se sente e quão bem você é capaz de fazer suas atividades de vida diária. Responda cada questão marcando a resposta como indicado. Caso você esteja inseguro em como responder, por favor, tente responder o melhor que puder.

1 - **Em geral você diria que sua saúde é: (Circule uma)**

Excelente 1
Muito Boa 2
Boa 3
Ruim 4
Muito Ruim 5

2 **- Comparada há 1 ano atrás,** como você classificaria sua saúde em geral, agora? **(Circule uma)**

Muito melhor agora do que há um ano atrás 1
Um pouco melhor agora do que há um ano atrás 2
Quase a mesma de um ano atrás 3
Um pouco pior agora do que há um ano atrás 4
Muito pior agora do que há um ano atrás 5

3 - Os seguintes itens são sobre atividades que você poderia fazer atualmente durante um dia comum. **Devido a sua saúde**, você tem dificuldade para fazer essas atividades? Neste caso, quanto? **(circule um número em cada linha)**

**Atividades**	**Sim dificulta muito**	**Sim dificulta um pouco**	**Não. Não dificulta de modo algum**
a - **Atividades vigorosas**, que exigem muito esforço: correr, levantar objetos pesados, participar em esportes árduos.	1	2	3
b – **Atividades moderadas**, tais como: mover uma mesa, passar aspirador de pó, jogar bola, varrer a	1	2	3

casa.			
c - Levantar ou carregar mantimentos	1	2	3
d - Subir **vários** lances de escada	1	2	3
e – Subir **um lance** de escada	1	2	3
f – Curvar-se, ajoelhar-se ou dobrar-se	1	2	3
g – Andar **mais de 1 quilômetro**	1	2	3
h – Andar **vários quarteirões**	1	2	3
i – Andar **um** quarteirão	1	2	3
j – Tomar banho ou vestir-se	1	2	3

4 – Durante **as últimas 4 semanas**,você teve algum dos seguintes problemas com o seu trabalho ou com alguma atividade diária regular, como conseqüência de sua saúde física?

	**Sim**	**Não**
a – Você diminuiu a **quantidade de tempo** que se dedicava ao seu trabalho ou a outras atividades?	1	2
b – Realizou **menos tarefas** do que você gostaria?	1	2
c – Esteve **limitado** no seu trabalho ou em outras atividades?	1	2
d – Teve **dificuldade** de fazer seu trabalho ou outras atividades? (necessitou de um esforço extra?)	1	2

5 – Durante as últimas 4 semanas, você teve algum dos seguintes problemas com o seu trabalho ou outra atividade regular diária,como conseqüência de algum problema emocional (como sentir-se deprimido ou ansioso)?

	**Sim**	**Não**
a – Você diminuiu a **quantidade de tempo** que se dedicava ao seu trabalho ou a outras atividades?	1	2
b – Realizou **menos tarefas** do que você gostaria?	1	2
c – Não trabalhou ou não fez qualquer das atividades com tanto **cuidado** como geralmente faz?	1	2

6 – Durante as **últimas 4 semanas**, de que maneira sua saúde física ou problemas emocionais interferiram nas suas atividades sociais normais, em relação a família, vizinhos, amigos ou em grupo? **(Circule uma)**

De forma nenhuma 1

Ligeiramente 2

Moderadamente 3

Bastante 4

Extremamente 5

7 – Quanta dor no corpo você teve durante **as últimas 4 semanas**?

Nenhuma 1

Muito Leve 2
Leve 3
Moderada 4
Grave 5
Muito Grave 6

8 – Durante **as últimas 4 semanas**, quanto a dor interferiu com o seu trabalho normal (incluindo tanto o trabalho, fora de casa e dentro de casa)?
De maneira alguma 1
Um pouco 2
Moderadamente 3
Bastante 4
Extremamente 5

9 – Estas questões são sobre como você se sente e como tudo tem acontecido com você durante as últimas 4 semanas. Para cada questão, por favor dê uma resposta que mais se aproxime da maneira como você se sente. Em relação as últimas 4 semanas.

	**Todo tempo**	**A maior parte do tempo**	**Uma boa parte do tempo**	**Alguma parte do tempo**	**Uma pequena parte do tempo**	**Nunca**
a - Quanto tempo você tem se sentido cheio de vigor, cheio de vontade, cheio de força?	**1**	**2**	**3**	**4**	**5**	**6**
b – Quanto tempo você tem se sentido uma pessoa muito nervosa?	**1**	**2**	**3**	**4**	**5**	**6**
c - Quanto tempo você tem se sentido tão deprimido que nada pode animá-lo?	**1**	**2**	**3**	**4**	**5**	**6**
d - Quanto tempo você tem se sentido calmo ou tranqüilo?	**1**	**2**	**3**	**4**	**5**	**6**
e - Quanto tempo você tem se sentido com muita energia?	**1**	**2**	**3**	**4**	**5**	**6**
f - Quanto tempo você tem se sentido desanimado e abatido?	**1**	**2**	**3**	**4**	**5**	**6**
g- Quanto tempo você tem se sentido esgotado?	**1**	**2**	**3**	**4**	**5**	**6**
h - Quanto tempo você tem se sentido uma pessoa feliz?	**1**	**2**	**3**	**4**	**5**	**6**

I - Quanto tempo você tem se sentido cansado?	**1**	**2**	**3**	**4**	**5**	**6**

10 – Durante as **últimas 4 semanas**, quanto do seu tempo a **sua saúde física ou problemas emocionais** interferiram com as suas atividades sociais (como visitar amigos, parentes, etc.)?

Todo o tempo 1
A maior parte do tempo 2
Alguma parte do tempo 3
Uma pequena parte do tempo 4
Nenhuma parte do tempo 5

11 – O quanto verdadeiro ou **falso** é cada uma das afirmações

	**Definitivamente verdadeiro**	**A maioria das vezes verdadeira**	**Não sei**	**A maioria das vezes falsa**	**Definitivamente falsa**
a – Eu costumo adoecer um pouco mais facilmente que as outras pessoas	1	2	3	4	5
b – Eu sou tão saudável quanto qualquer pessoa que eu conheço.	1	2	3	4	5
c – Eu acho que a minha saúde vai piorar	1	2	3	4	5
d – Minha saúde é excelente	1	2	3	4	5
